# Supplementary material for: Use of CT, ED presentation and hospitalisations 12 months before and after a diagnosis of cancer in Western Australia: a population-based retrospective cohort study
Source: BMJ Open. 2023 Oct 29;13(10):e071052. doi: 10.1136/bmjopen-2022-071052 (PMC10619095; doi:10.1136/bmjopen-2022-071052)
Supplement: Supplementary data [file bmjopen-2022-071052supp001.pdf]

Appendix

The use of computed tomography, ED presentation and hospitalisations 12-months before and after a diagnosis of cancer in Western Australia: A population-based retrospective cohort study

Author information

Ninh T. Ha[1], PhD, thi.ha@curtin.edu.au

Sviatlana Kamarova[1], PhD, sviatlana.kamarova@curtin.edu.au

David Youens[1], david.youens@curtin.edu.au

Chau Ho[1,] chau.ho@curtin.edu.au

Max Bulsara [2,3], PhD, max.bulsara@nd.edu.au

Jenny Doust[4], BMBS, PhD, j.doust@uq.edu.au

Donald McRobbie[5], PhD, donald.mcrobbie@adelaide.edu.au

Peter O'Leary [1,6,7], PhD, peter.oleary@health.wa.gov.au

Cameron Wright[1,8,9,10], MD, cameron.wright@curtin.edu.au

Richard Trevithick[11], MD, richard.trevithick@health.wa.gov.au

Rachael Moorin,[1,3] PhD, R.Moorin@curtin.edu.au

Affiliations

1. Health Economics and Data Analytics, Curtin School of Population Health, Faculty of Health Sciences, Curtin University, Western Australia;
2. Institute for Health Research, University of Notre Dame, Western Australia;

3. Centre for Health Services Research, School of Population and Global Health, The University of Western Australia;
4. Centre for Longitudinal and Life Course Research, Faculty of Medicine, University of Queensland;
5. School of Physical Sciences, University of Adelaide;
6. Obstetrics and Gynaecology Medical School, Faculty of Health and Medical Sciences, The University of Western Australia, Perth, Western Australia, Australia;
7. PathWest Laboratory Medicine, QE2 Medical Centre, Nedlands, Western Australia, Australia;
8. Fiona Stanley Hospital, 11 Robin Warren Dr, Murdoch, Western Australia, Australia;
9. Division of Internal Medicine, Medical School, Faculty of Health and Medical Sciences, The University of Western Australia;
10. School of Medicine, College of Health and Medicine, University of Tasmania, Hobart, Tasmania;
11. Western Australian Cancer Registry, Clinical Excellence Division, Department of Health, East Perth, Western Australia.

***Corresponding author:**

Ninh Thi Ha

Health Economics & Data Analytics, Curtin School of Population Health, Faculty of Health Sciences, Curtin University

GPO Box U1987, Perth, Western Australia, 6845

Email: Thi.Ha@curtin.edu.au; ninh.ha05@gmail.com

Phone: +61 08 9266 5134

Appendix A. Multivariable negative binomial regression of monthly use of CT scanning, ED presentation, and hospitalisations within 12 Months before and after Cancer Diagnosis over the study period.

	CT scan		Hospitalisations		ED presentations	
	RR	95%CI	RR	95%CI	RR	95%CI
**Month pre& post cancer diagnosis**						
-12	1	(1; 1)	1	(1; 1)	1	(1; 1)
-11	1	(0.92; 1.09)	1.04*	(1.00; 1.08)	1.03	(0.97; 1.10)
-10	1.04	(0.96; 1.14)	1.05**	(1.01; 1.10)	1.04	(0.98; 1.10)
-9	1.04	(0.95; 1.13)	1.07**	(1.02; 1.11)	1.07*	(1.01; 1.14)
-8	1.12**	(1.03; 1.21)	1.07***	(1.03; 1.12)	1.09**	(1.02; 1.15)
-7	1.17***	(1.08; 1.28)	1.16***	(1.11; 1.21)	1.12***	(1.05; 1.19)
-6	1.27***	(1.17; 1.37)	1.14***	(1.09; 1.19)	1.16***	(1.09; 1.23)
-5	1.40***	(1.29; 1.52)	1.19***	(1.14; 1.25)	1.24***	(1.17; 1.31)
-4	1.75***	(1.62; 1.89)	1.32***	(1.27; 1.38)	1.41***	(1.33; 1.50)
-3	2.52***	(2.34; 2.71)	1.49***	(1.43; 1.55)	1.66***	(1.57; 1.76)
-2	4.94***	(4.61; 5.28)	1.91***	(1.82; 1.99)	2.26***	(2.14; 2.39)
-1	20.6***	(19.3; 21.9)	7.57***	(7.22; 7.94)	6.62***	(6.30; 6.95)
1	34.9***	(32.8; 37.2)	18.7***	(17.8; 19.6)	4.74***	(4.51; 4.98)
2	14.4***	(13.5; 15.4)	12.4***	(11.8; 13.0)	3.92***	(3.72; 4.12)
3	11.0***	(10.3; 11.8)	12.7***	(12.1; 13.3)	3.52***	(3.35; 3.71)
4	9.67***	(9.05; 10.3)	11.7***	(11.2; 12.3)	3.19***	(3.03; 3.36)
5	8.79***	(8.22; 9.39)	10.1***	(9.61; 10.6)	2.85***	(2.70; 3.01)
6	8.75***	(8.19; 9.36)	8.75***	(8.33; 9.18)	2.68***	(2.54; 2.83)
7	8.85***	(8.28; 9.46)	7.52***	(7.16; 7.89)	2.41***	(2.28; 2.55)
8	8.26***	(7.73; 8.83)	6.31***	(6.01; 6.63)	2.31***	(2.19; 2.45)
9	7.48***	(6.99; 8.01)	5.19***	(4.95; 5.45)	2.17***	(2.05; 2.30)
10	7.10***	(6.63; 7.59)	4.70***	(4.48; 4.94)	2.16***	(2.04; 2.29)
11	6.99***	(6.53; 7.48)	4.42***	(4.21; 4.65)	2.01***	(1.90; 2.13)
12	7.07***	(6.61; 7.57)	4.23***	(4.03; 4.44)	1.97***	(1.86; 2.09)
**Cancer group**						
Solid cancer	1	(1; 1)	1	(1; 1)	1	(1; 1)
ALH cancer[†]	1.25*	(1.02; 1.53)	0.99	(0.84; 1.17)	1.02	(0.87; 1.20)
**Interaction term of months # cancer groups**						
-12 # solid cancer	1	(1; 1)	1	(1; 1)	1	(1; 1)
-12 # ALH	1	(1; 1)	1	(1; 1)	1	(1; 1)
-11 # solid cancer	1	(1; 1)	1	(1; 1)	1	(1; 1)
-11 # ALH	1.02	(0.77; 1.34)	1.14*	(1.02; 1.29)	1	(0.82; 1.22)
-10 # solid cancer	1	(1; 1)	1	(1; 1)	1	(1; 1)
-10 # ALH	0.85	(0.64; 1.11)	1.04	(0.92; 1.16)	1.05	(0.87; 1.27)
-9 # solid cancer	1	(1; 1)	1	(1; 1)	1	(1; 1)
-9 # ALH	1.14	(0.88; 1.48)	1.09	(0.97; 1.22)	1	(0.83; 1.21)
-8 # solid cancer	1	(1; 1)	1	(1; 1)	1	(1; 1)
-8 # ALH	1.02	(0.78; 1.32)	1.13*	(1.00; 1.27)	1.07	(0.89; 1.28)
-7 # solid cancer	1	(1; 1)	1	(1; 1)	1	(1; 1)
-7 # ALH	0.88	(0.67; 1.14)	1.04	(0.92; 1.18)	1.11	(0.91; 1.35)

	CT scan		Hospitalisations		ED presentations	
	RR	95%CI	RR	95%CI	RR	95%CI
-6 # solid cancer	1	(1; 1)	1	(1; 1)	1	(1; 1)
-6 # ALH	1.08	(0.84; 1.38)	1.20**	(1.06; 1.35)	1.14	(0.95; 1.38)
-5 # solid cancer	1	(1; 1)	1	(1; 1)	1	(1; 1)
-5 # ALH	1.11	(0.87; 1.43)	1.11	(0.98; 1.26)	1.05	(0.87; 1.28)
-4 # solid cancer	1	(1; 1)	1	(1; 1)	1	(1; 1)
-4 # ALH	1.04	(0.82; 1.33)	1.12	(0.98; 1.27)	1.08	(0.90; 1.31)
-3 # solid cancer	1	(1; 1)	1	(1; 1)	1	(1; 1)
-3 # ALH	1.02	(0.81; 1.30)	1.15*	(1.01; 1.31)	1.12	(0.94; 1.33)
-2 # solid cancer	1	(1; 1)	1	(1; 1)	1	(1; 1)
-2 # ALH	1.11	(0.89; 1.38)	1.22**	(1.06; 1.40)	1.21*	(1.02; 1.43)
-1 # solid cancer	1	(1; 1)	1	(1; 1)	1	(1; 1)
-1 # ALH	1.31**	(1.07; 1.61)	1.17*	(1.00; 1.38)	1.63***	(1.39; 1.92)
1 # solid cancer	1	(1; 1)	1	(1; 1)	1	(1; 1)
1 # ALH	0.92	(0.74; 1.13)	1.13	(0.96; 1.34)	1.30**	(1.11; 1.54)
2 # solid cancer	1	(1; 1)	1	(1; 1)	1	(1; 1)
2 # ALH	0.79*	(0.64; 0.97)	1.49***	(1.26; 1.76)	1.34***	(1.13; 1.59)
3 # solid cancer	1	(1; 1)	1	(1; 1)	1	(1; 1)
3 # ALH	0.87	(0.70; 1.08)	1.44***	(1.22; 1.70)	1.35***	(1.14; 1.60)
4 # solid cancer	1	(1; 1)	1	(1; 1)	1	(1; 1)
4 # ALH	0.96	(0.77; 1.19)	1.42***	(1.21; 1.68)	1.31**	(1.11; 1.56)
5 # solid cancer	1	(1; 1)	1	(1; 1)	1	(1; 1)
5 # ALH	0.89	(0.72; 1.11)	1.37***	(1.16; 1.62)	1.23*	(1.04; 1.47)
6 # solid cancer	1	(1; 1)	1	(1; 1)	1	(1; 1)
6 # ALH	0.88	(0.70; 1.09)	1.26**	(1.06; 1.48)	1.19*	(1.00; 1.42)
7 # solid cancer	1	(1; 1)	1	(1; 1)	1	(1; 1)
7 # ALH	0.80*	(0.64; 0.99)	1.21*	(1.02; 1.43)	1.13	(0.95; 1.35)
8 # solid cancer	1	(1; 1)	1	(1; 1)	1	(1; 1)
8 # ALH	0.79*	(0.63; 0.99)	1.27**	(1.07; 1.50)	1.19	(0.99; 1.42)
9 # solid cancer	1	(1; 1)	1	(1; 1)	1	(1; 1)
9 # ALH	0.73**	(0.58; 0.91)	1.39***	(1.17; 1.64)	1.14	(0.95; 1.37)
10 # solid cancer	1	(1; 1)	1	(1; 1)	1	(1; 1)
10 # ALH	0.76*	(0.60; 0.95)	1.43***	(1.21; 1.70)	1.1	(0.92; 1.33)
11 # solid cancer	1	(1; 1)	1	(1; 1)	1	(1; 1)
11 # ALH	0.71**	(0.57; 0.90)	1.49***	(1.26; 1.77)	1.24*	(1.03; 1.49)
12 # solid cancer	1	(1; 1)	1	(1; 1)	1	(1; 1)
12 # ALH	0.75*	(0.59; 0.94)	1.52***	(1.28; 1.80)	1.23*	(1.03; 1.49)
**Year of cancer diagnosis**						
2004	1	(1; 1)	1	(1; 1)	1	(1; 1)
2005	1.05*	(1.00; 1.09)	0.98	(0.93; 1.04)	0.96	(0.89; 1.04)
2006	0.94**	(0.90; 0.98)	0.91**	(0.86; 0.96)	0.94	(0.87; 1.02)
2007	0.96	(0.93; 1.00)	0.98	(0.92; 1.03)	0.93	(0.86; 1.00)
2008	0.97	(0.93; 1.00)	0.97	(0.91; 1.02)	0.94	(0.87; 1.01)
2009	0.92***	(0.88; 0.95)	0.99	(0.94; 1.05)	0.98	(0.91; 1.06)
2010	0.91***	(0.88; 0.95)	1.02	(0.96; 1.07)	1.03	(0.95; 1.11)

	CT scan		Hospitalisations		ED presentations	
	RR	95%CI	RR	95%CI	RR	95%CI
2011	0.94**	(0.90; 0.97)	1.02	(0.97; 1.07)	1.04	(0.96; 1.12)
2012	0.95**	(0.92; 0.99)	1.02	(0.97; 1.08)	1.09*	(1.01; 1.18)
2013	0.96*	(0.93; 1.00)	1.03	(0.97; 1.09)	1.07	(0.99; 1.16)
2014	0.96	(0.93; 1.00)	0.97	(0.92; 1.03)	1.04	(0.96; 1.12)
**Sex**						
Male	1	(1; 1)	1	(1; 1)	1	(1; 1)
Female	1	(0.99; 1.02)	1.13***	(1.11; 1.16)	0.98*	(0.96; 1.00)
**Age- group at diagnosis**						
18-45 years	1	(1; 1)	1	(1; 1)	1	(1; 1)
46-65 years	1.02	(0.99; 1.05)	0.96**	(0.93; 0.99)	0.76***	(0.73; 0.79)
66-75 years	0.96**	(0.93; 0.99)	0.84***	(0.81; 0.87)	0.73***	(0.70; 0.76)
>75 years	0.74***	(0.72; 0.77)	0.66***	(0.63; 0.68)	0.84***	(0.80; 0.87)
**Socio-economic status**‡						
Least disadvantage	1	(1; 1)	1	(1; 1)	1	(1; 1)
Less disadvantage	1.08***	(1.05; 1.11)	1.01	(0.98; 1.05)	1.30***	(1.26; 1.35)
Moderate disadvantage	1.07***	(1.05; 1.10)	1.01	(0.98; 1.04)	1.45***	(1.41; 1.49)
High disadvantage	1.12***	(1.10; 1.15)	1.01	(0.98; 1.05)	1.65***	(1.60; 1.69)
Highest disadvantage	1.12***	(1.08; 1.15)	0.99	(0.94; 1.03)	1.75***	(1.67; 1.84)
Unknown	1.1	(0.97; 1.26)	1.06	(0.81; 1.40)	1.12	(0.94; 1.33)
**Accessibility to services**§						
Major cities	1	(1; 1)	1	(1; 1)	1	(1; 1)
Inner regional	0.87***	(0.85; 0.89)	0.96**	(0.93; 0.99)	1.19***	(1.15; 1.22)
Outer regional	0.84***	(0.81; 0.86)	0.91***	(0.87; 0.94)	1.52***	(1.47; 1.58)
Remote	0.84***	(0.79; 0.91)	0.83***	(0.78; 0.88)	1.69***	(1.58; 1.81)
Very remote	0.87***	(0.83; 0.91)	0.83***	(0.78; 0.89)	2.33***	(2.21; 2.47)
Unknown	0.96	(0.75; 1.23)	0.67**	(0.50; 0.90)	1.03	(0.76; 1.39)
**Comorbidities**¶						
0	1	(1; 1)	1	(1; 1)	1	(1; 1)
1-2	1.87***	(1.75; 2.00)	3.74***	(3.44; 4.06)	1.19***	(1.11; 1.29)
3-5	3.00***	(2.81; 3.21)	6.58***	(6.06; 7.15)	2.23***	(2.07; 2.40)
6+	4.94***	(4.62; 5.27)	11.5***	(10.5; 12.5)	5.54***	(5.15; 5.96)
**Basis of diagnosis**						
Non-pathological	1	(1; 1)	1	(1; 1)	1	(1; 1)
Pathological	0.95*	(0.91; 0.99)	1.08	(0.96; 1.20)	0.66***	(0.63; 0.69)
**Hospital catchment region**						
Other	1	(1; 1)	1	(1; 1)	1	(1; 1)
Bentley and Armadale	1.04***	(1.02; 1.07)	1.01	(0.98; 1.05)	0.99	(0.95; 1.03)

* p <0.05, ** p <0.01, *** p <0.001

CT: Computed tomography, ED: emergency department, IRR: incidence rate ratio· ALH: All haematopoietic cancer.

RRs were also adjusted for interactions between time in months before and after cancer diagnosis and cancer groups; time in years of cancer diagnosis cohort and cancer groups; and Indigenous status. Please note Indigenous status cannot be reported as per restrictions imposed by the human research ethics approval and can only be used for model adjustment purposes. Month post diagnosis = 1 includes the day diagnosed.

Appendix C. Multivariable negative binomial regression of total use of CT scanning, ED presentation, and hospitalisations in a year before diagnosis with cancer over the study period

	CT scan		Hospitalisations		ED presentations	
	RR	95%CI	RR	95%CI	RR	95%CI
**Cancer group**						
Solid cancer	1	(1; 1)	1	(1; 1)	1	(1; 1)
ALH cancer	1.56***	(1.39; 1.75)	1.07	(0.96; 1.19)	1.31***	(1.14; 1.50)
**Year of cancer diagnosis**						
2004	1	(1; 1)	1	(1; 1)	1	(1; 1)
2005	1.01	(0.96; 1.07)	0.98	(0.93; 1.03)	0.97	(0.91; 1.04)
2006	0.93*	(0.88; 0.99)	0.90***	(0.86; 0.95)	0.96	(0.90; 1.02)
2007	0.95	(0.90; 1.01)	0.91***	(0.87; 0.96)	0.93*	(0.88; 1.00)
2008	0.92**	(0.88; 0.98)	0.88***	(0.84; 0.92)	0.93*	(0.87; 0.99)
2009	0.96	(0.91; 1.01)	0.90***	(0.86; 0.94)	0.97	(0.92; 1.04)
2010	0.97	(0.92; 1.02)	0.90***	(0.86; 0.94)	1.02	(0.96; 1.09)
2011	0.94*	(0.89; 0.99)	0.89***	(0.85; 0.93)	1.00	(0.94; 1.06)
2012	0.97	(0.92; 1.03)	0.91***	(0.87; 0.95)	1.10**	(1.04; 1.16)
2013	1.00	(0.95; 1.06)	0.94*	(0.90; 0.99)	1.05	(0.99; 1.11)
2014	1.00	(0.95; 1.06)	0.88***	(0.85; 0.92)	0.97	(0.91; 1.03)
**Interaction term of cancer diagnosis year and cancer type**						
2004 # solid cancer	1	(1; 1)	1	(1; 1)	1	(1; 1)
2004 # ALH	1	(1; 1)	1	(1; 1)	1	(1; 1)
2005 # solid cancer	1	(1; 1)	1	(1; 1)	1	(1; 1)
2005 # ALH	0.80*	(0.68; 0.95)	0.93	(0.80; 1.08)	0.93	(0.77; 1.13)
2006 # solid cancer	1	(1; 1)	1	(1; 1)	1	(1; 1)
2006 # ALH	0.98	(0.83; 1.16)	0.94	(0.81; 1.10)	1.03	(0.85; 1.25)
2007 # solid cancer	1	(1; 1)	1	(1; 1)	1	(1; 1)
2007 # ALH	0.95	(0.81; 1.12)	1.06	(0.91; 1.23)	0.98	(0.81; 1.19)
2008 # solid cancer	1	(1; 1)	1	(1; 1)	1	(1; 1)
2008 # ALH	0.92	(0.78; 1.08)	1.18*	(1.01; 1.36)	0.95	(0.79; 1.15)
2009 # solid cancer	1	(1; 1)	1	(1; 1)	1	(1; 1)
2009 # ALH	1.02	(0.87; 1.19)	0.90	(0.77; 1.04)	0.95	(0.79; 1.14)
2010 # solid cancer	1	(1; 1)	1	(1; 1)	1	(1; 1)
2010 # ALH	0.95	(0.81; 1.11)	1.08	(0.93; 1.24)	0.94	(0.78; 1.12)
2011 # solid cancer	1	(1; 1)	1	(1; 1)	1	(1; 1)
2011 # ALH	0.95	(0.81; 1.10)	1.03	(0.89; 1.18)	0.97	(0.82; 1.16)
2012 # solid cancer	1	(1; 1)	1	(1; 1)	1	(1; 1)
2012 # ALH	0.90	(0.77; 1.05)	0.98	(0.85; 1.13)	0.96	(0.80; 1.14)
2013 # solid cancer	1	(1; 1)	1	(1; 1)	1	(1; 1)
2013 # ALH	0.90	(0.77; 1.05)	1.06	(0.92; 1.21)	1.02	(0.86; 1.22)
2014 # solid cancer	1	(1; 1)	1	(1; 1)	1	(1; 1)
2014 # ALH	0.94	(0.81; 1.09)	0.97	(0.85; 1.12)	1.07	(0.90; 1.27)
**Sex**						
Male	1	(1; 1)	1	(1; 1)	1	(1; 1)
Female	0.94***	(0.92; 0.96)	0.78***	(0.77; 0.79)	0.92***	(0.90; 0.94)

	**CT scan**		**Hospitalisations**		**ED presentations**	
	**RR**	**95%CI**	**RR**	**95%CI**	**RR**	**95%CI**
**Age- group at diagnosis**						
18-45 years	1	(1; 1)	1	(1; 1)	1	(1; 1)
46-65 years	1.00	(0.96; 1.04)	1.05**	(1.01; 1.09)	0.70***	(0.68; 0.73)
66-75 years	1.07**	(1.02; 1.11)	1.25***	(1.21; 1.30)	0.68***	(0.65; 0.71)
>75 years	0.94**	(0.91; 0.98)	1.38***	(1.34; 1.44)	0.86***	(0.82; 0.89)
**Socio-economic status**						
Least disadvantage	1	(1; 1)	1	(1; 1)	1	(1; 1)
Less disadvantage	1.07***	(1.03; 1.10)	1.03*	(1.01; 1.06)	1.28***	(1.23; 1.33)
Moderate disadvantage	1.09***	(1.06; 1.12)	1.04**	(1.01; 1.07)	1.43***	(1.38; 1.48)
High disadvantage	1.15***	(1.11; 1.18)	1.04***	(1.02; 1.07)	1.60***	(1.55; 1.66)
Highest disadvantage	1.15***	(1.10; 1.19)	1.01	(0.98; 1.04)	1.72***	(1.65; 1.79)
Unknown	1.22*	(1.01; 1.47)	1.53***	(1.31; 1.77)	0.91	(0.72; 1.14)
**Accessibility to services**						
Major cities	1	(1; 1)	1	(1; 1)	1	(1; 1)
Inner regional	0.84***	(0.82; 0.87)	0.97*	(0.95; 1.00)	1.12***	(1.09; 1.16)
Outer regional	0.81***	(0.77; 0.84)	0.92***	(0.89; 0.96)	1.46***	(1.40; 1.52)
Remote	0.75***	(0.69; 0.83)	0.84***	(0.77; 0.91)	1.69***	(1.55; 1.84)
Very remote	0.78***	(0.74; 0.84)	0.85***	(0.81; 0.90)	2.49***	(2.35; 2.63)
Unknown	0.86	(0.62; 1.19)	0.41***	(0.30; 0.58)	1.10	(0.74; 1.64)
**Number of comorbidities**						
0	1	(1; 1)	1	(1; 1)	1	(1; 1)
1-2	2.28***	(2.08; 2.49)	180.9***	(90.4; 362.2)	1.20***	(1.10; 1.31)
3-5	3.93***	(3.60; 4.29)	430.7***	(215.2; 861.7)	2.62***	(2.41; 2.85)
6+	6.76***	(6.20; 7.37)	1026.5***	(513.0; 2053.8)	6.74***	(6.21; 7.32)
**Basis of diagnosis**						
Non-pathological	1	(1; 1)	1	(1; 1)	1	(1; 1)
Pathological	0.96	(0.91; 1.01)	0.95*	(0.91; 0.99)	0.65***	(0.62; 0.68)
**Hospital catchment region**						
Other	1	(1; 1)	1	(1; 1)	1	(1; 1)
Bentley and Armadale	1.03*	(1.00; 1.06)	0.99	(0.97; 1.02)	0.99	(0.96; 1.02)

* p <0.05, ** p <0.01, *** p <0.001

CT: Computed tomography, ED: emergency department, RR: incidence rate ratio, ALH: All haematopoietic cancer.

RRs were also adjusted for interactions between time in months before and after cancer diagnosis and cancer groups; time in years of cancer diagnosis cohort and cancer groups; and Indigenous status. Please note Indigenous status cannot be reported as per restrictions imposed by the human research ethics approval and can only be used for model adjustment purposes. Month post diagnosis = 1 includes the day diagnosed..

Appendix D. Multivariable negative binomial regression of total use of CT scanning, ED presentation, and hospitalisations in a year after diagnosis with cancer over the study period

	CT scan		Hospitalisations		ED presentations	
	RR	95%CI	RR	95%CI	RR	95%CI
**Cancer group**						
Solid cancer	1	(1; 1)	1	(1; 1)	1	(1; 1)
ALH cancer	1.70***	(1.53; 1.89)	1.43***	(1.31; 1.55)	1.31***	(1.15; 1.49)
**Year of cancer diagnosis**						
2004	1	(1; 1)	1	(1; 1)	1	(1; 1)
2005	1.09***	(1.04; 1.15)	0.98	(0.95; 1.02)	0.97	(0.91; 1.03)
2006	0.97	(0.93; 1.01)	0.92***	(0.89; 0.96)	0.95	(0.90; 1.01)
2007	1.03	(0.99; 1.08)	0.99	(0.96; 1.03)	0.95	(0.89; 1.01)
2008	1.07**	(1.02; 1.12)	1.00	(0.96; 1.03)	0.99	(0.94; 1.05)
2009	0.99	(0.95; 1.04)	1.04*	(1.00; 1.08)	1.02	(0.97; 1.08)
2010	0.99	(0.95; 1.04)	1.07***	(1.03; 1.10)	1.08**	(1.02; 1.14)
2011	1.05*	(1.01; 1.10)	1.10***	(1.06; 1.14)	1.12***	(1.06; 1.19)
2012	1.04	(1.00; 1.09)	1.08***	(1.04; 1.11)	1.16***	(1.10; 1.22)
2013	1.05*	(1.00; 1.10)	1.07***	(1.03; 1.11)	1.14***	(1.08; 1.21)
2014	1.06**	(1.01; 1.11)	1.02	(0.99; 1.06)	1.14***	(1.08; 1.20)
**Interaction term of cancer diagnosis year and cancer type**						
2004 # solid cancer	1	(1; 1)	1	(1; 1)	1	(1; 1)
2004 # ALH	1	(1; 1)	1	(1; 1)	1	(1; 1)
2005 # solid cancer	1	(1; 1)	1	(1; 1)	1	(1; 1)
2005 # ALH	0.84*	(0.72; 0.97)	1.01	(0.90; 1.14)	1.04	(0.87; 1.25)
2006 # solid cancer	1	(1; 1)	1	(1; 1)	1	(1; 1)
2006 # ALH	0.83*	(0.72; 0.96)	0.96	(0.86; 1.09)	0.93	(0.78; 1.12)
2007 # solid cancer	1	(1; 1)	1	(1; 1)	1	(1; 1)
2007 # ALH	0.70***	(0.60; 0.81)	1.01	(0.90; 1.14)	1.06	(0.89; 1.28)
2008 # solid cancer	1	(1; 1)	1	(1; 1)	1	(1; 1)
2008 # ALH	0.65***	(0.56; 0.75)	1.06	(0.94; 1.19)	0.99	(0.82; 1.18)
2009 # solid cancer	1	(1; 1)	1	(1; 1)	1	(1; 1)
2009 # ALH	0.59***	(0.51; 0.68)	0.98	(0.87; 1.10)	1.07	(0.90; 1.28)
2010 # solid cancer	1	(1; 1)	1	(1; 1)	1	(1; 1)
2010 # ALH	0.48***	(0.42; 0.56)	0.94	(0.84; 1.05)	1.09	(0.92; 1.29)
2011 # solid cancer	1	(1; 1)	1	(1; 1)	1	(1; 1)
2011 # ALH	0.51***	(0.44; 0.58)	0.88*	(0.79; 0.98)	0.99	(0.84; 1.17)
2012 # solid cancer	1	(1; 1)	1	(1; 1)	1	(1; 1)
2012 # ALH	0.56***	(0.48; 0.64)	0.97	(0.87; 1.08)	0.92	(0.78; 1.09)
2013 # solid cancer	1	(1; 1)	1	(1; 1)	1	(1; 1)
2013 # ALH	0.55***	(0.48; 0.63)	0.93	(0.83; 1.04)	1.03	(0.87; 1.21)
2014 # solid cancer	1	(1; 1)	1	(1; 1)	1	(1; 1)
2014 # ALH	0.51***	(0.45; 0.59)	1.04	(0.93; 1.16)	1.03	(0.87; 1.21)
**Sex**						
Male	1	(1; 1)	1	(1; 1)	1	(1; 1)

	CT scan		Hospitalisations		ED presentations	
	**RR**	**95%CI**	**RR**	**95%CI**	**RR**	**95%CI**
Female	1.04***	(1.03; 1.06)	1.24***	(1.22; 1.26)	1.03**	(1.01; 1.06)
**Age- group at diagnosis**						
18-45 years	1	(1; 1)	1	(1; 1)	1	(1; 1)
46-65 years	0.98	(0.95; 1.01)	0.94***	(0.91; 0.96)	0.77***	(0.74; 0.80)
66-75 years	0.85***	(0.83; 0.88)	0.75***	(0.73; 0.76)	0.73***	(0.70; 0.76)
>75 years	0.59***	(0.57; 0.61)	0.47***	(0.46; 0.49)	0.74***	(0.71; 0.77)
**Socio-economic status**						
Least disadvantage	1	(1; 1)	1	(1; 1)	1	(1; 1)
Less disadvantage	1.07***	(1.04; 1.10)	1.00	(0.98; 1.03)	1.32***	(1.28; 1.37)
Moderate disadvantage	1.05***	(1.02; 1.08)	1.00	(0.98; 1.03)	1.49***	(1.45; 1.54)
High disadvantage	1.08***	(1.05; 1.11)	1.00	(0.98; 1.02)	1.69***	(1.63; 1.74)
Highest disadvantage	1.07***	(1.04; 1.11)	0.97*	(0.94; 0.99)	1.76***	(1.69; 1.83)
Unknown	1.04	(0.89; 1.22)	0.93	(0.83; 1.06)	1.32**	(1.09; 1.60)
**Accessibility to services**						
Major cities	1	(1; 1)	1	(1; 1)	1	(1; 1)
Inner regional	0.87***	(0.85; 0.90)	0.95***	(0.93; 0.97)	1.23***	(1.19; 1.27)
Outer regional	0.85***	(0.82; 0.88)	0.89***	(0.87; 0.92)	1.59***	(1.53; 1.65)
Remote	0.87***	(0.80; 0.94)	0.83***	(0.78; 0.88)	1.74***	(1.60; 1.88)
Very remote	0.92**	(0.88; 0.97)	0.83***	(0.79; 0.86)	2.33***	(2.20; 2.46)
Unknown	1.03	(0.80; 1.34)	0.77*	(0.62; 0.95)	0.95	(0.68; 1.33)
**Number of comorbidities**						
0	1	(1; 1)	1	(1; 1)	1	(1; 1)
1-2	1.81***	(1.72; 1.91)	3.54***	(3.39; 3.70)	1.18***	(1.10; 1.26)
3-5	2.77***	(2.63; 2.92)	5.96***	(5.71; 6.23)	2.01***	(1.89; 2.15)
6+	4.03***	(3.82; 4.24)	8.29***	(7.94; 8.66)	4.55***	(4.26; 4.86)
**Basis of diagnosis**						
Non-pathological	1	(1; 1)	1	(1; 1)	1	(1; 1)
Pathological	1.29***	(1.22; 1.35)	1.84***	(1.77; 1.92)	0.89***	(0.84; 0.94)
**Hospital catchment region**						
Other	1	(1; 1)	1	(1; 1)	1	(1; 1)
Bentley and Armadale	1.03*	(1.00; 1.06)	1.00	(0.98; 1.02)	0.97	(0.94; 1.00)

* *p* <0.05, ** *p* <0.01, *** *p* <0.001

CT: Computed tomography, ED: emergency department, RR: incidence rate ratio, ALH: All haematopoietic cancer.

RRs were also adjusted for interactions between time in months before and after cancer diagnosis and cancer groups; time in years of cancer diagnosis cohort and cancer groups; and Indigenous status. Please note Indigenous status cannot be reported as per restrictions imposed by the human research ethics approval and can only be used for model adjustment purposes. Month post diagnosis = 1 includes the day diagnosed
