# Supplementary material for: Use of CT, ED presentation and hospitalisations 12 months before and after a diagnosis of cancer in Western Australia: a population-based retrospective cohort study
Source: BMJ Open. 2023 Oct 29;13(10):e071052. doi: 10.1136/bmjopen-2022-071052 (PMC10619095; doi:10.1136/bmjopen-2022-071052)
Supplement: Supplementary data [file bmjopen-2022-071052supp002.pdf]

BMJ Publishing Group Limited (BMJ) disclaims all liability and responsibility arising from any reliance placed on this supplemental material which has been supplied by the author(s)

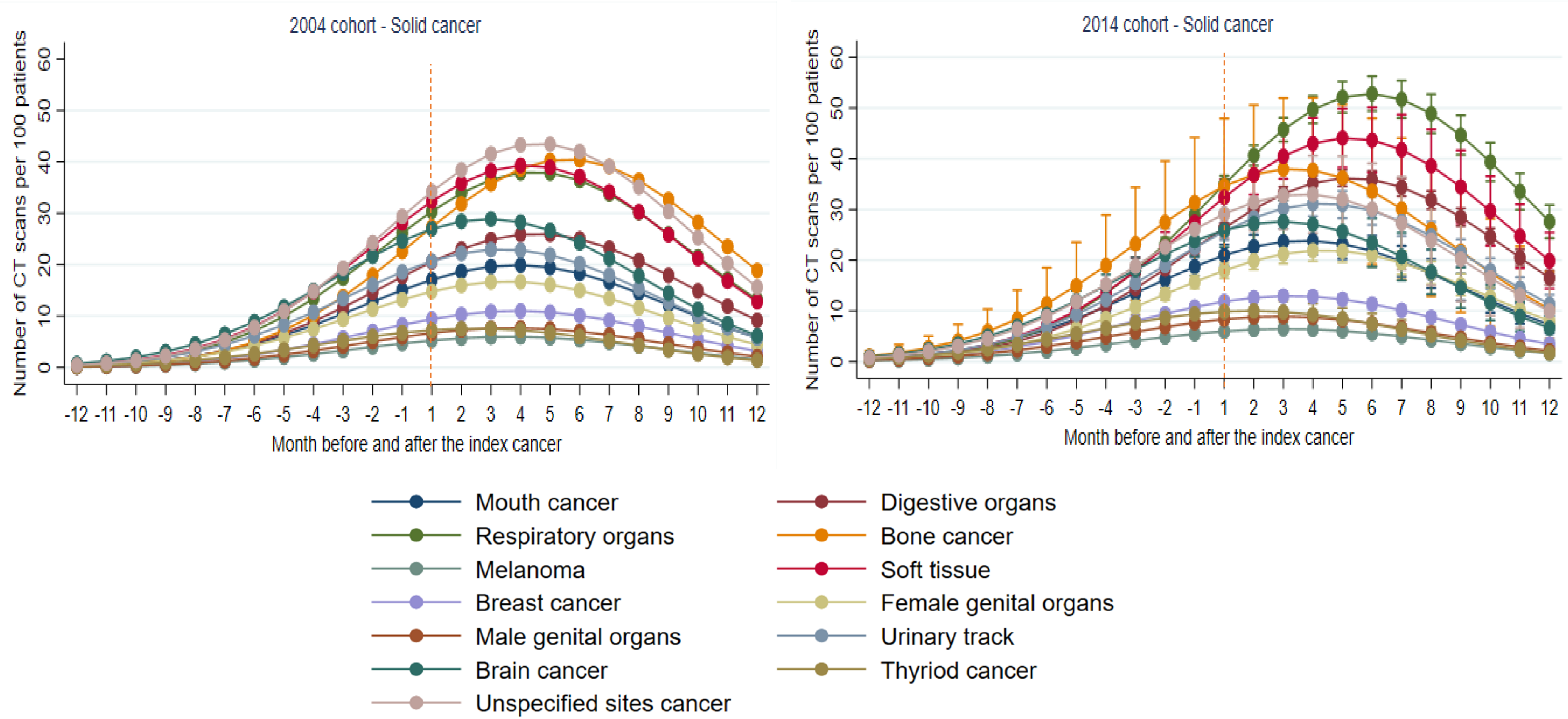

Appendix B1A. Trend in CT use around the cancer diagnosis window for solid cancer (12 months pre and post the index diagnosis)

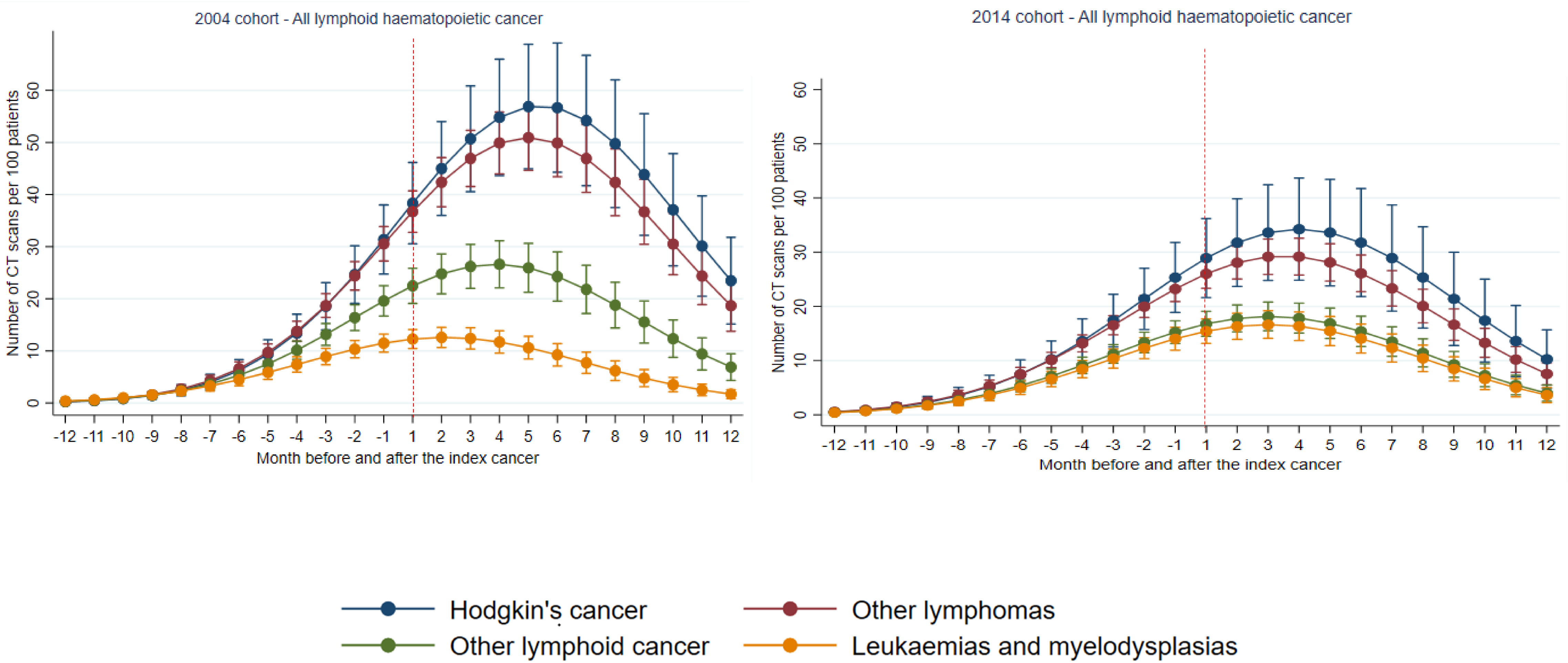

Appendix B1B. Trend in CT use around the cancer diagnosis window for ALH cancer (12 months pre and post the index diagnosis)

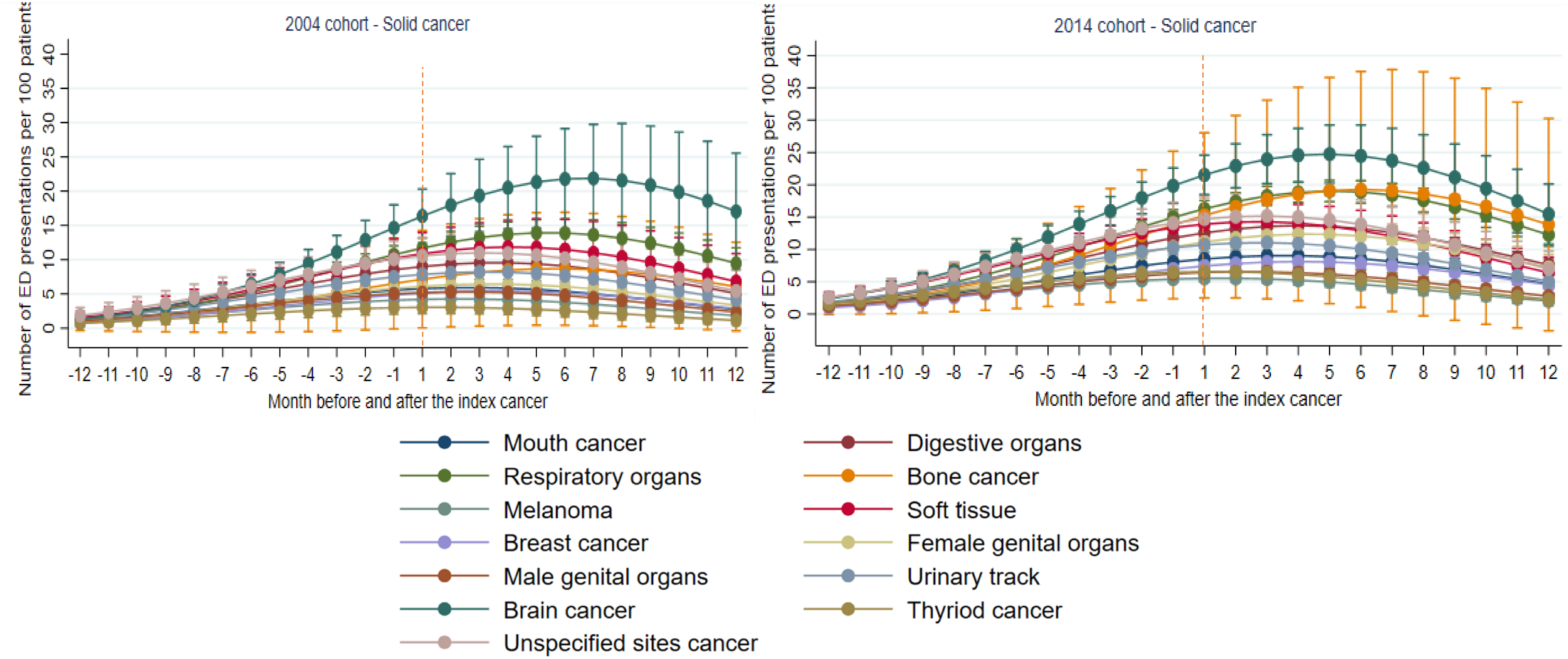

Appendix B2A. Trend in ED presentations around the cancer diagnosis window for solid cancer (12 months pre and post the index diagnosis)

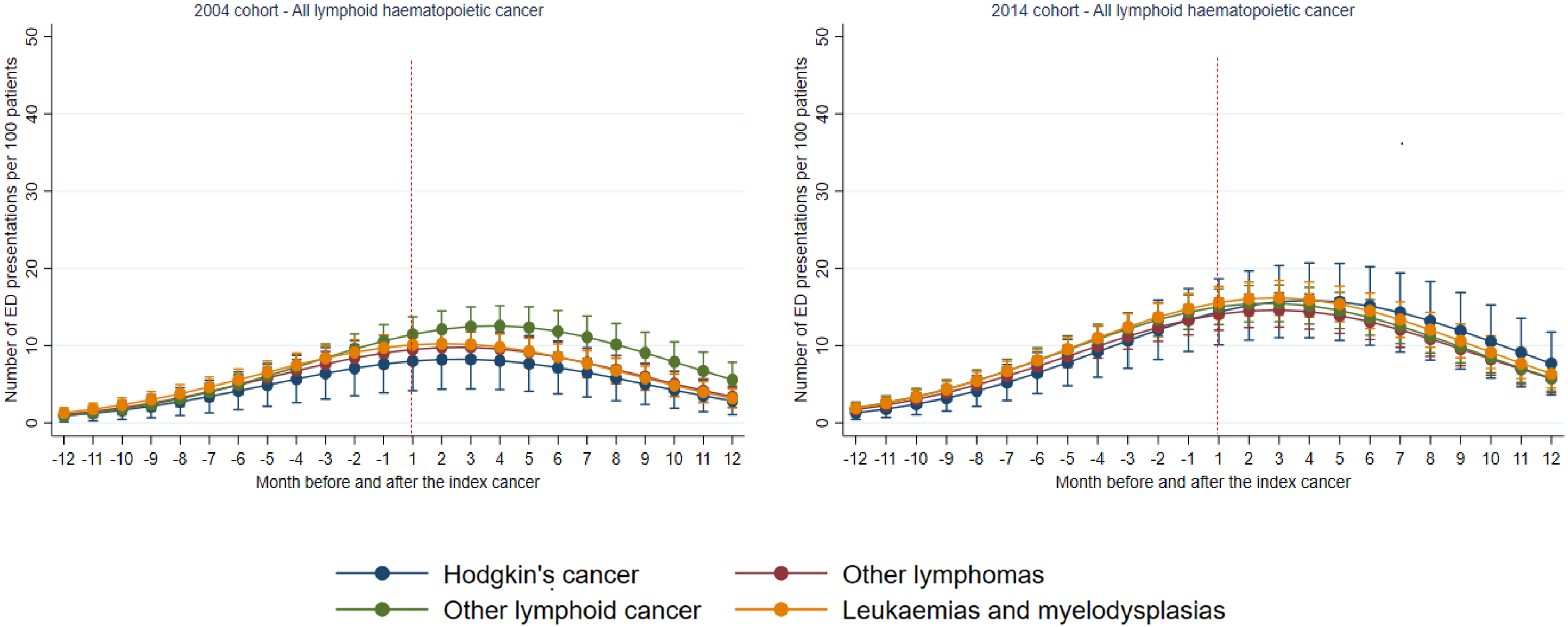

Appendix B2B. Trend in ED presentations around the cancer diagnosis window for ALH cancer (12 months pre and post the index diagnosis)

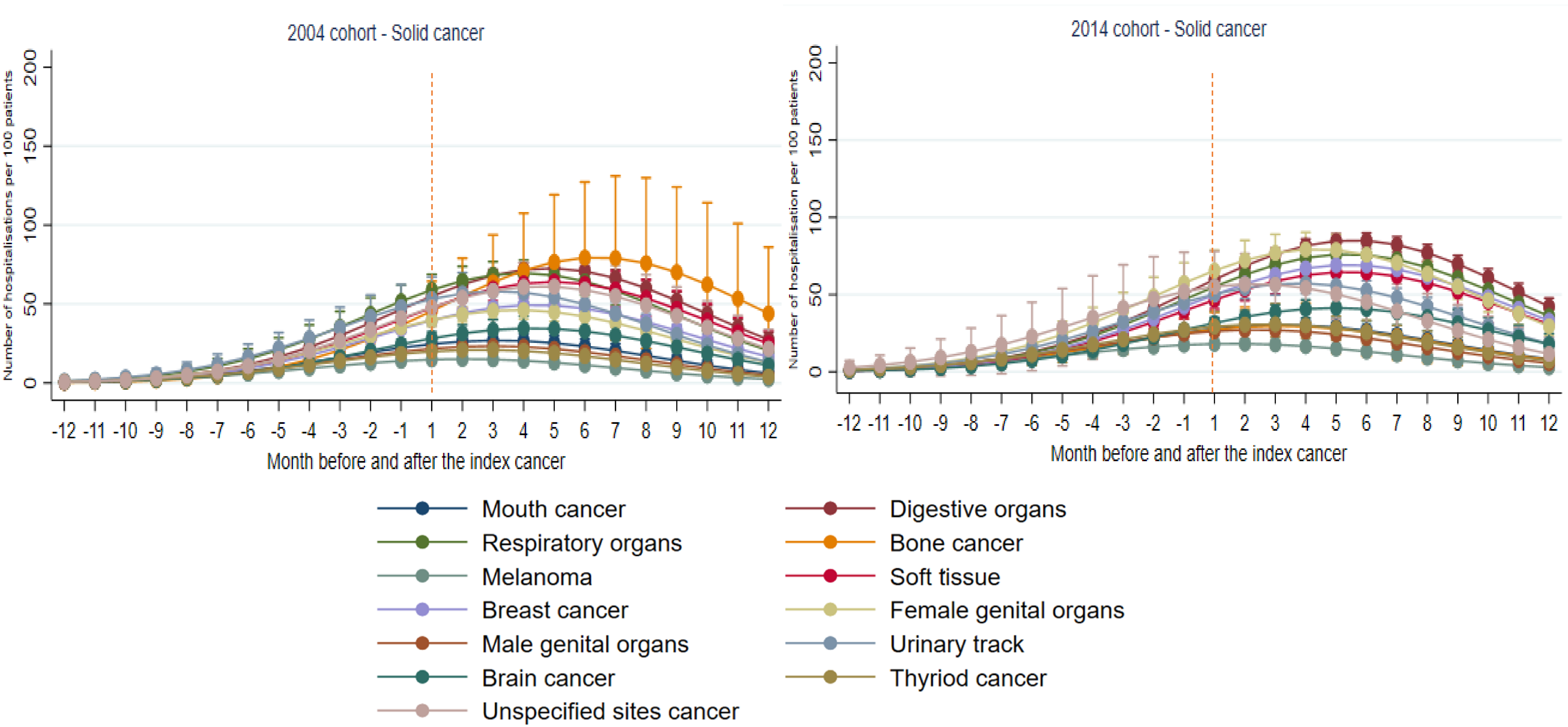

Appendix B3A. Trend in hospitalisation around the cancer diagnosis window for solid cancer (12 months pre and post the index diagnosis)

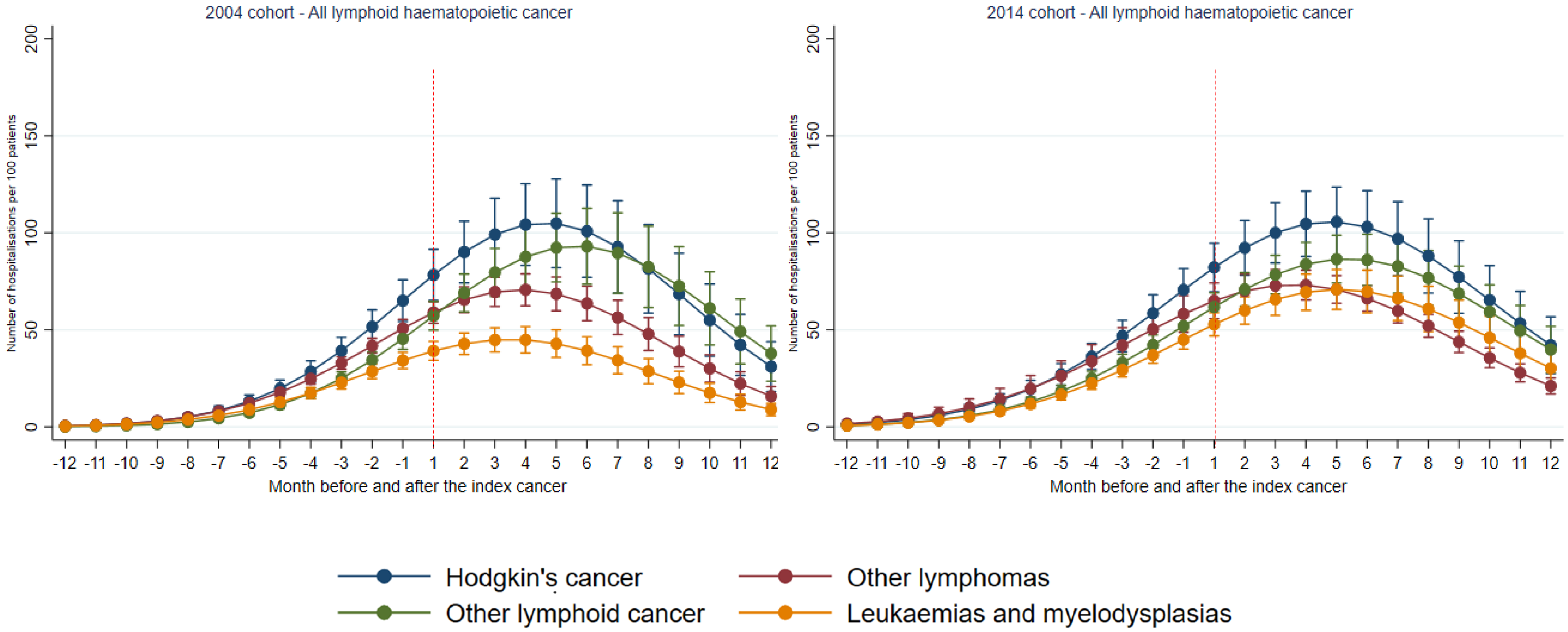

Appendix B3B. Trend in hospitalisation around the cancer diagnosis window for ALH cancer (12 months pre and post the index diagnosis)
