# Supplementary material for: Use of CT, ED presentation and hospitalisations 12 months before and after a diagnosis of cancer in Western Australia: a population-based retrospective cohort study
Source: BMJ Open. 2023 Oct 29;13(10):e071052. doi: 10.1136/bmjopen-2022-071052 (PMC10619095; doi:10.1136/bmjopen-2022-071052)
Supplement: Supplementary data [file bmjopen-2022-071052supp003.pdf]

BMJ Publishing Group Limited (BMJ) disclaims all liability and responsibility arising from any reliance placed on this supplemental material which has been supplied by the author(s)

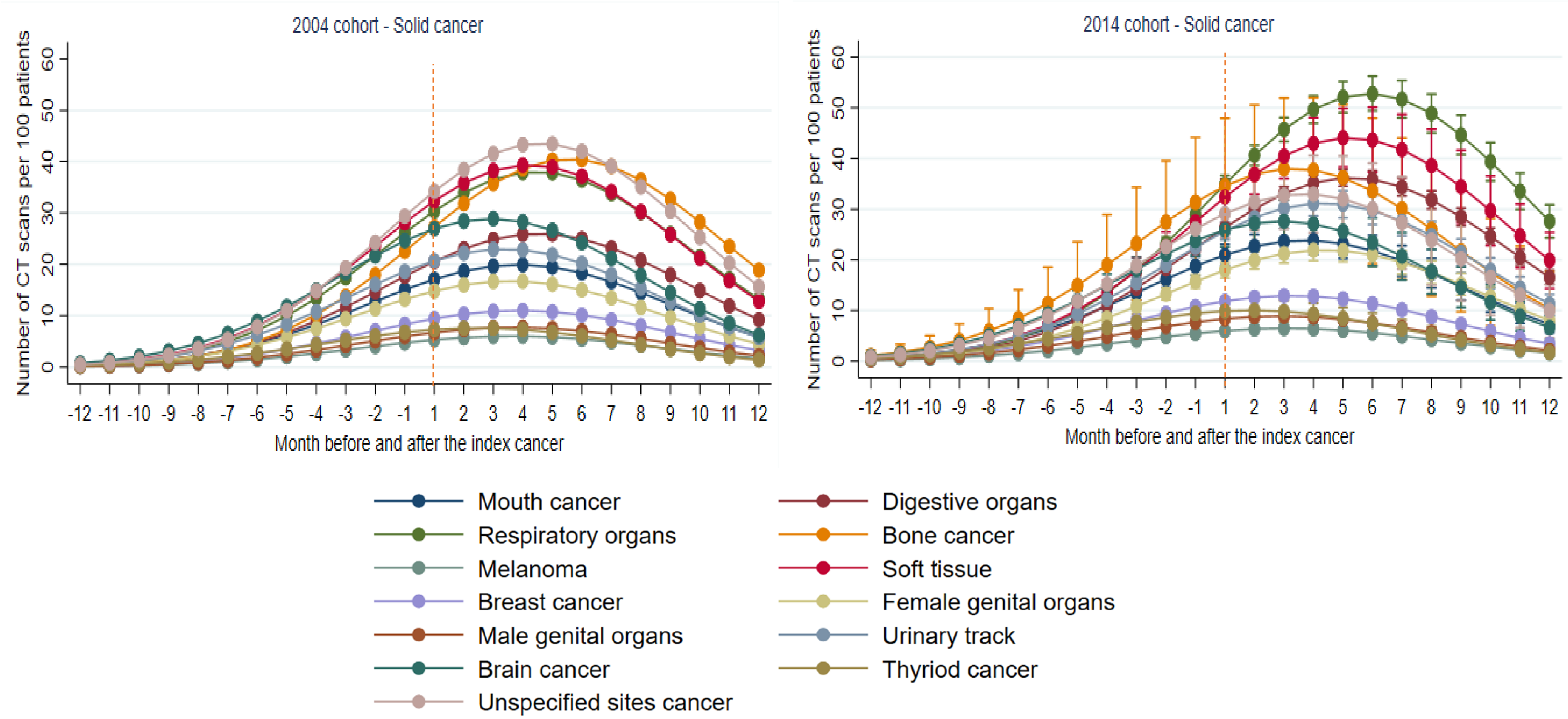

Appendix D1A. Trend in CT use around the cancer diagnosis window for solid cancer (12 months pre and post the index diagnosis)

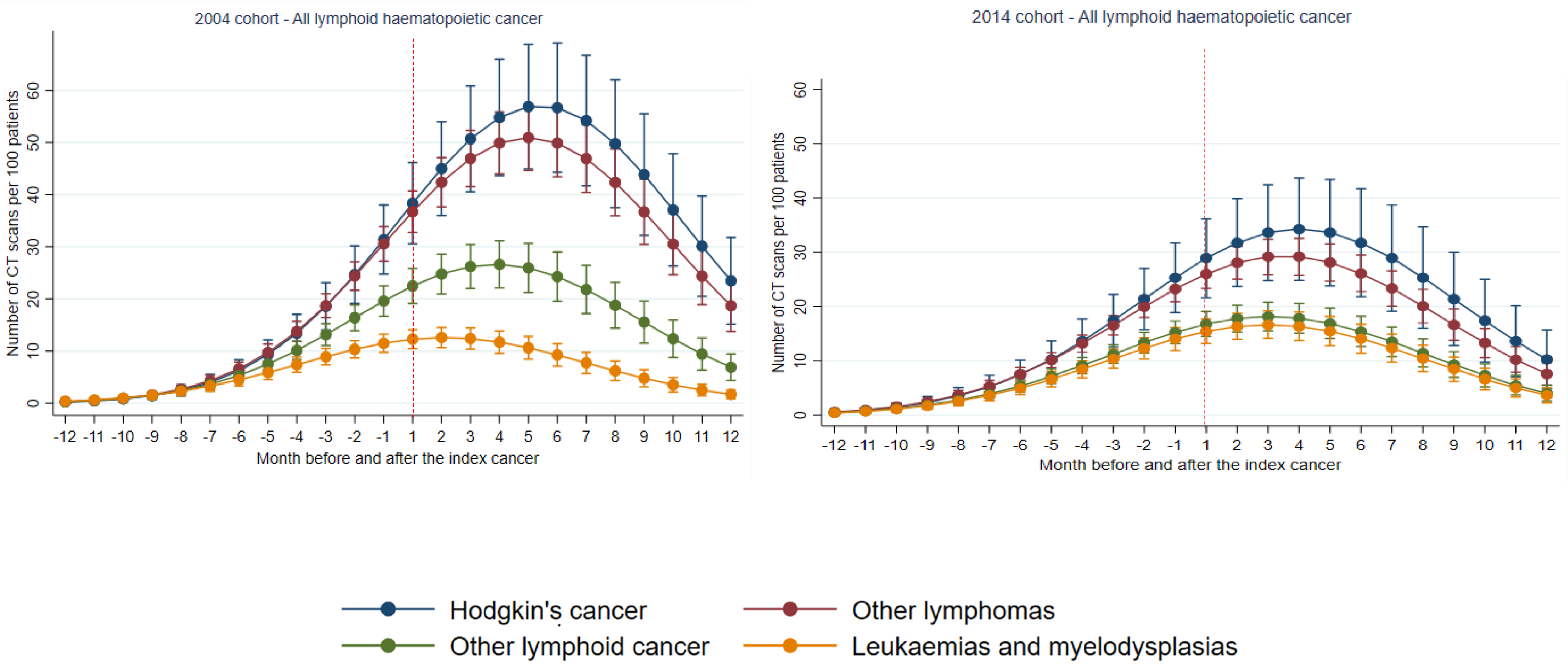

Appendix D1B. Trend in CT use around the cancer diagnosis window for ALH cancer (12 months pre and post the index diagnosis)

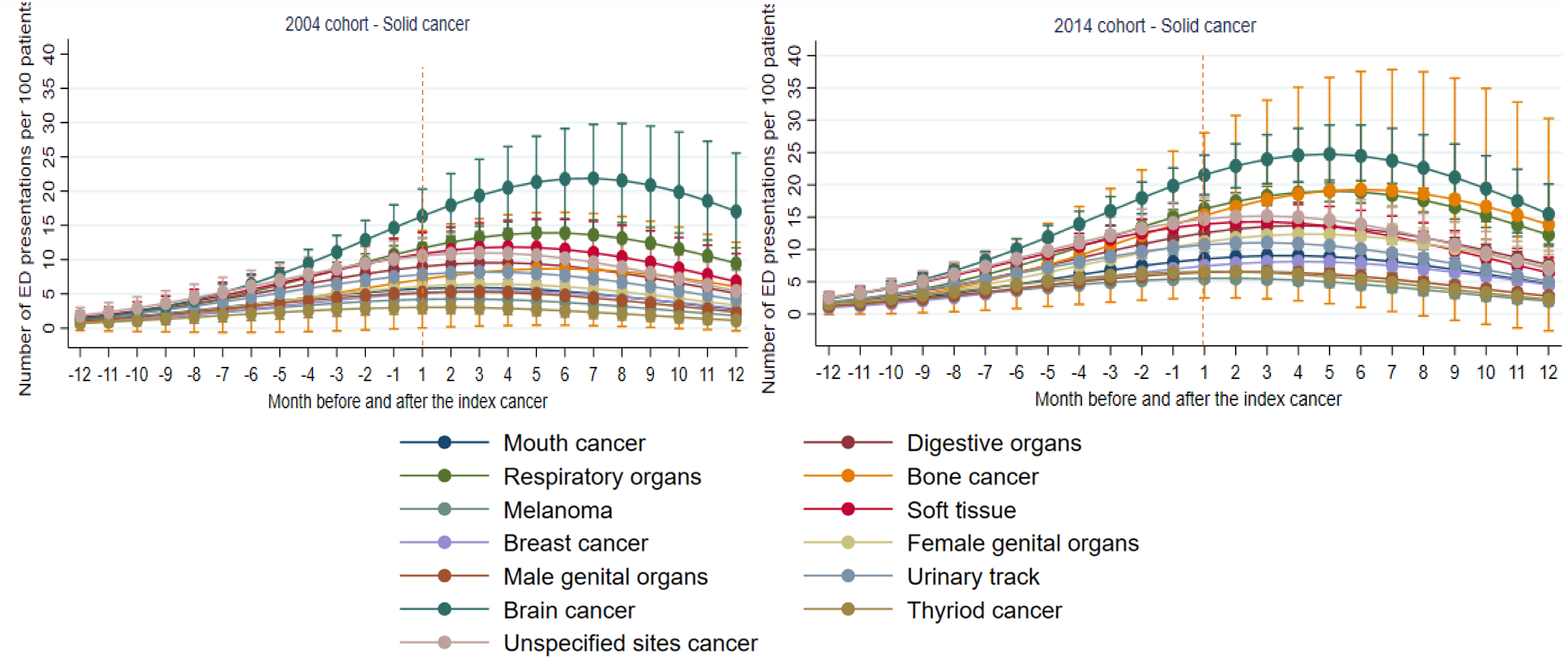

Appendix D2A. Trend in ED presentations around the cancer diagnosis window for solid cancer (12 months pre and post the index diagnosis)

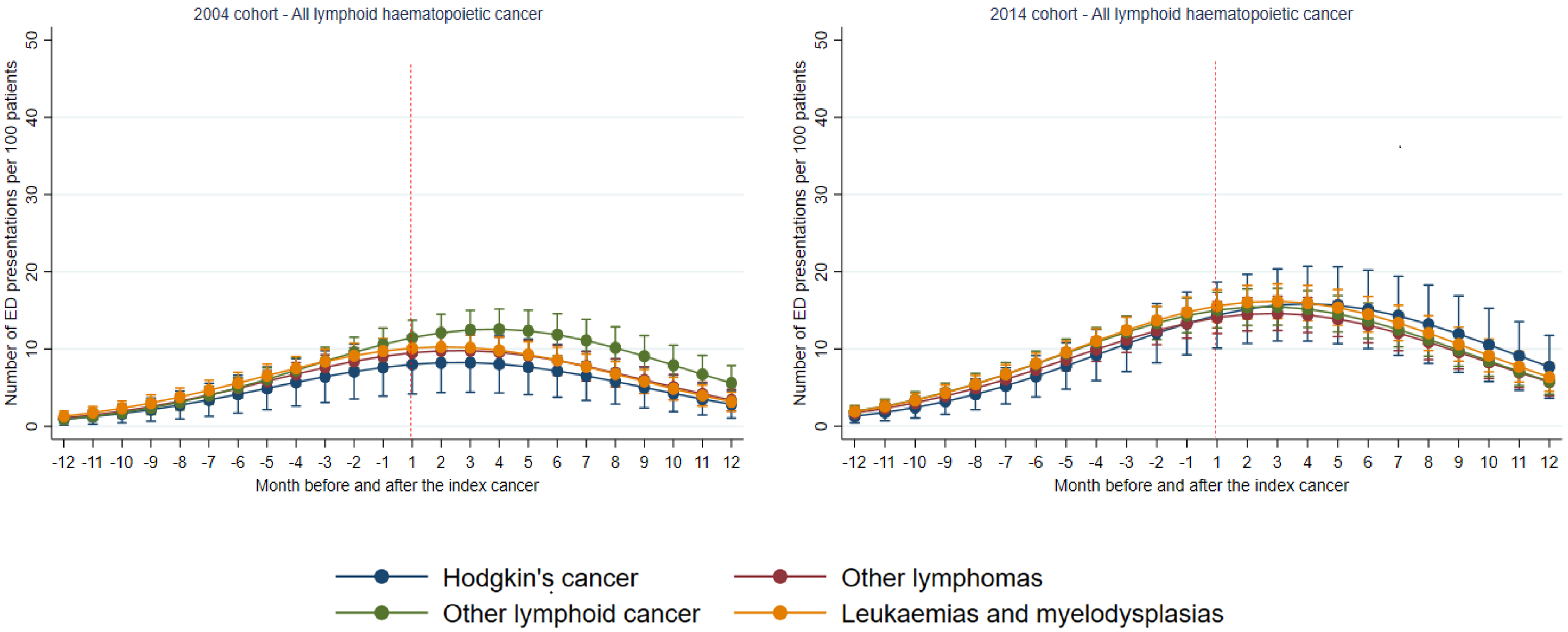

Appendix D2B. Trend in ED presentations around the cancer diagnosis window for ALH cancer (12 months pre and post the index diagnosis)

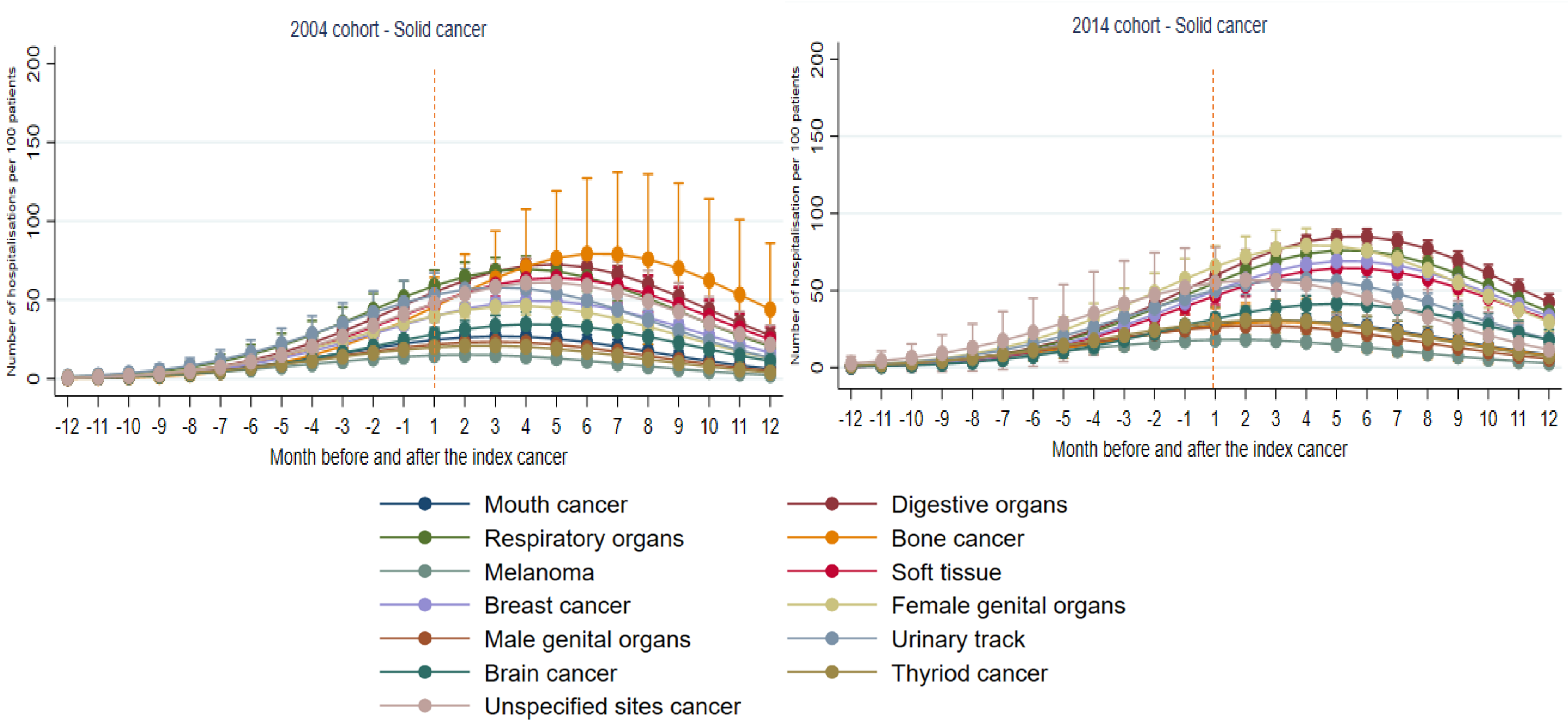

Appendix D3A. Trend in hospitalisation around the cancer diagnosis window for solid cancer (12 months pre and post the index diagnosis)

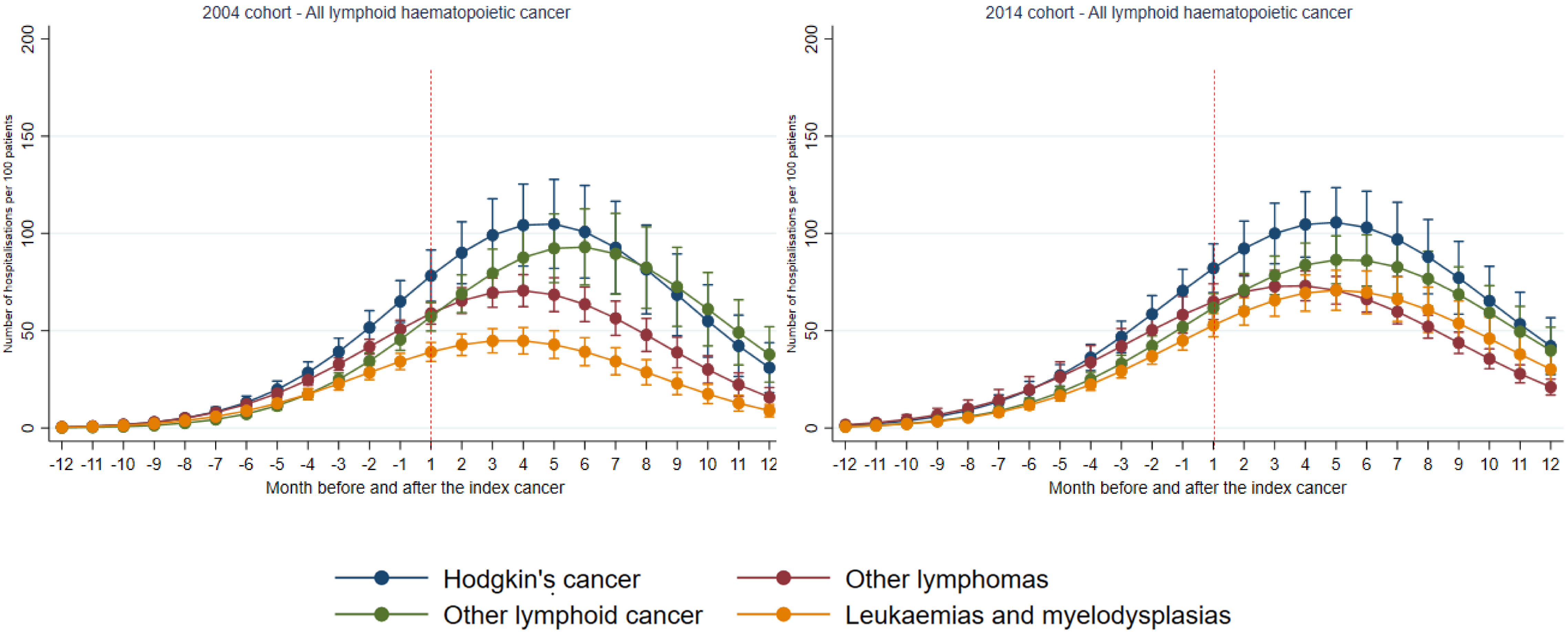

Appendix D3B. Trend in hospitalisation around the cancer diagnosis window for ALH cancer (12 months pre and post the index diagnosis)
