# Supplementary material for: Use of CT, ED presentation and hospitalisations 12 months before and after a diagnosis of cancer in Western Australia: a population-based retrospective cohort study
Source: BMJ Open. 2023 Oct 29;13(10):e071052. doi: 10.1136/bmjopen-2022-071052 (PMC10619095; doi:10.1136/bmjopen-2022-071052)
Supplement: Author's manuscript [file bmjopen-2022-071052.draft_revisions.pdf]

BMJ Open is committed to open peer review. As part of this commitment we make the peer review history of every article we publish publicly available.

When an article is published we post the peer reviewers' comments and the authors' responses online. We also post the versions of the paper that were used during peer review. These are the versions that the peer review comments apply to.

The versions of the paper that follow are the versions that were submitted during the peer review process. They are not the versions of record or the final published versions. They should not be cited or distributed as the published version of this manuscript.

BMJ Open is an open access journal and the full, final, typeset and author-corrected version of record of the manuscript is available on our site with no access controls, subscription charges or pay-per-view fees (http://bmjopen.bmj.com).

If you have any questions on BMJ Open's open peer review process please email info.bmjopen@bmj.com

BMJ Open

The use of computed tomography, ED presentation and hospitalisations 12-months before and after a diagnosis of cancer in Western Australia: A population-based retrospective cohort study

Journal:	*BMJ Open*
Manuscript ID	bmjopen-2022-071052
Article Type:	Original research
Date Submitted by the Author:	13-Dec-2022
Complete List of Authors:	Ha, Ninh; Curtin University Bentley Campus,<br>Kamarova, Sviatlana; Curtin University, School of Population Health<br>Youens, David; Curtin University, School of Population Health<br>Ho, Chau; Curtin University Bentley Campus,<br>Bulsara, Max; University of Notre Dame, Biostatistics<br>Doust, Jenny; The University of Queensland Faculty of Medicine and Biomedical Sciences<br>Mcrobbie, Donald; University of Adelaide , Medical Physics<br>O'Leary, Peter ; Curtin University Bentley Campus, School of Public Health; The University of Western Australia, Obstetrics and Gynaecology Medical School, Faculty of Health and Medical Sciences<br>Wright, Cameron; University of Tasmania, ; School of Medicine<br>Trevithick, Richard ; 11. Western Australian Cancer Registry, Clinical Excellence Division, Department of Health<br>Moorin, Rachael; Curtin University, ; University of Western Australia,
Keywords:	Computed tomography < RADIOLOGY & IMAGING, Health policy < HEALTH SERVICES ADMINISTRATION & MANAGEMENT, Quality in health care < HEALTH SERVICES ADMINISTRATION & MANAGEMENT, Public health < INFECTIOUS DISEASES, Epidemiology < ONCOLOGY

SCHOLARONE™
Manuscripts

I, the Submitting Author has the right to grant and does grant on behalf of all authors of the Work (as defined in the below author licence), an exclusive licence and/or a non-exclusive licence for contributions from authors who are: i) UK Crown employees; ii) where BMJ has agreed a CC-BY licence shall apply, and/or iii) in accordance with the terms applicable for US Federal Government officers or employees acting as part of their official duties; on a worldwide, perpetual, irrevocable, royalty-free basis to BMJ Publishing Group Ltd ("BMJ") its licensees and where the relevant Journal is co-owned by BMJ to the co-owners of the Journal, to publish the Work in this journal and any other BMJ products and to exploit all rights, as set out in our licence.

The Submitting Author accepts and understands that any supply made under these terms is made by BMJ to the Submitting Author unless you are acting as an employee on behalf of your employer or a postgraduate student of an affiliated institution which is paying any applicable article publishing charge ("APC") for Open Access articles. Where the Submitting Author wishes to make the Work available on an Open Access basis (and intends to pay the relevant APC), the terms of reuse of such Open Access shall be governed by a Creative Commons licence – details of these licences and which Creative Commons licence will apply to this Work are set out in our licence referred to above.

Other than as permitted in any relevant BMJ Author's Self Archiving Policies, I confirm this Work has not been accepted for publication elsewhere, is not being considered for publication elsewhere and does not duplicate material already published. I confirm all authors consent to publication of this Work and authorise the granting of this licence.

For peer review only

The use of computed tomography, ED presentation and hospitalisations 12-months before and after a diagnosis of cancer in Western Australia: A population-based retrospective cohort study

Author information

Ninh T. Ha[1], PhD, thi.ha@curtin.edu.au

Sviatlana Kamarova[1], PhD, sviatlana.kamarova@curtin.edu.au

David Youens[1], david.youens@curtin.edu.au

Chau Ho[1,] chau.ho@curtin.edu.au

Max Bulsara [2,3], PhD, max.bulsara@nd.edu.au

Jenny Doust[4], BMBS, PhD, j.doust@uq.edu.au

Donald McRobbie[5], PhD, donald.mcrobbie@adelaide.edu.au

Peter O'Leary [1,6,7], PhD, peter.oleary@health.wa.gov.au

Cameron Wright[1,8,9,10], MD, cameron.wright@curtin.edu.au

Richard Trevithick[11], MD, richard.trevithick@health.wa.gov.au

Rachael Moorin,[1,3] PhD, R.Moorin@curtin.edu.au

Affiliations

1. Health Economics and Data Analytics, Curtin School of Population Health, Faculty of Health Sciences, Curtin University, Western Australia;
2. Institute for Health Research, University of Notre Dame, Western Australia;
3. Centre for Health Services Research, School of Population and Global Health, The University of Western Australia;

For peer review only

4. Centre for Longitudinal and Life Course Research, Faculty of Medicine, University of Queensland;

5. School of Physical Sciences, University of Adelaide;

6. Obstetrics and Gynaecology Medical School, Faculty of Health and Medical Sciences, The University of Western Australia, Perth, Western Australia, Australia;

7. PathWest Laboratory Medicine, QE2 Medical Centre, Nedlands, Western Australia, Australia;

8. Fiona Stanley Hospital, 11 Robin Warren Dr, Murdoch, Western Australia, Australia;

9. Division of Internal Medicine, Medical School, Faculty of Health and Medical Sciences, The University of Western Australia;

10. School of Medicine, College of Health and Medicine, University of Tasmania, Hobart, Tasmania;

11. Western Australian Cancer Registry, Clinical Excellence Division, Department of Health, East Perth, Western Australia.

***Corresponding author:**

Ninh Thi Ha

Health Economics & Data Analytics, Curtin School of Population Health, Faculty of Health Sciences, Curtin University

GPO Box U1987, Perth, Western Australia, 6845

Email: Thi.Ha@curtin.edu.au; ninh.ha05@gmail.com

Phone: +61 08 9266 5134

For peer review only

Abstract (300 max)

Objective: To examine the use of CT, ED-presentation and hospitalisation and in 12-month before and after a diagnosis of cancer.

Design: Population-based retrospective cohort study.

Setting: West Australian linked administrative records at individual level.

Participants: 104,009 adults newly diagnosed with cancer in 2004-2014

Main outcome measures: CT use, ED presentations, hospitalisations.

Methods: Descriptive analyses were used to summarise the study population characteristics at the beginning of the study (2004), the end of the study (2014) and overall, across the study period (2004-2014). Negative binomial regression was performed to calculate the monthly rate of CT scans, ED presentations, and hospitalisation for each of 12-months before and after a cancer diagnosis.

Results: As compared to the rates in the 12th month before diagnosis, the rate of CT scans started to increase from two months before diagnosis with an increase in both ED presentations and hospitalisation from one month before the diagnosis. These rates peaked in the month of diagnosis for CT scans (477 [95%CI 471-482] per 1000 patients), and for hospitalisations (910 [95%CI 902-919] per 1000 patients), and the month prior to diagnosis for ED (181 [95%CI 178-184] per 1000 patients) then rapidly reduced after diagnosis but remained high for the next 12 months. While the patterns of the health services used were similar between 2004 and 2014, the rate of the health services used during after diagnosis was higher in 2014 vs. 2004 except for CT use in patients with lymphohematopoietic cancer with a significant reduction.

For peer review only

Conclusion: Our results showed an increase in demand for health services from two months before diagnosis of cancer. Increasing use of health services during and post cancer diagnosis may warrant further investigation to identify factors driving this change.

Keywords: computed tomography, ED presentations, hospitalisation, cancer, health services

Strengths and limitations of the study:

- The present study examines (i) the patterns of hospital and ED services in conjunction with (ii) both pre and post diagnostic periods of CT use, which has not been evaluated to date in adult cancer patients.
- The cohort makes use of whole of population data which removes selection bias and minimises loss to follow up, while allowing for grounded and representative conclusions for the target population based on higher statistical precision.
- The present data does not include some important clinical data such as indications for CT scanning and stage of cancer at diagnosis. Some CT scans from secondary hospitals may be missing.

For peer review only

Background

Computed tomography (CT) is useful for screening for cancer; diagnosis of the presence of a tumour and staging of cancer; guiding where to perform a biopsy procedure, where to apply local treatment (e.g. radiofrequency ablation, implantation of radioactive seeds or cryotherapy); planning surgery, directing treatment strategies and assessing treatment response (1-4). Examples of the utility of CT include the National CT colonography trial, where CT colonography provided similar accuracy in the rate of identifying both large colorectal polyps and colorectal tumours in patients with an average risk of colorectal cancer as compared to the gold standard colonoscopy (1). Similarly in the National Lung Screening trial, compared to chest X-ray, screening by CT reduced the risk of mortality from lung cancer in heavy smokers by 20% (2). However, compared to standard X-ray (and non-ionising radiation modalities such as ultrasound and magnetic resonance imaging), CT scanning imparts a relatively high radiation exposure that has raised concerns about the risk of cancer, particularly in children, young adults and women (5-8). In addition to the radiation burden increased use of CT scanning has also been associated with increases in incidental findings and over-diagnosis (9, 10). There is therefore a trade-off between the benefits and risk of CT scanning which has led to calls to evaluate its use to ensure maintenance of high value care.

CT scanning has been steadily increasingly in most developed countries, particularly in cancer care (11-16). Studies in the UK (17) and Denmark (18) reported a significant increase in CT scanning from three months before a cancer diagnosis. A study by Purdie *et al* in New South Wales, Australia observed a similar pattern (19). However, this study included only a small number of participants (n=894) and was limited to the period before a cancer diagnosis. While patterns of visits/consultation by GPs or specialists before and after a cancer diagnosis have been examined previously (20, 21), patterns of ED presentations and hospitalisations in Australia remained unclear. Thus, in the present study, we examined changes in the use of CT,

For peer review only

ED presentations and hospitalisations in the 12-months before and after a diagnosis of cancer for adults diagnosed with cancer between 2004 and 2014 in Western Australia (WA) using linked administrative data.

Methods

Study design

This population-based retrospective cohort study follows the REporting of studies Conducted using Observational Routinely-collected health Data (RECORD) statement (22). Approval was granted by the Western Australia (WA) Department of Health Human Research Ethics Committee (HREC) (2011/97) and the AIHW HREC (EO2018/4/485), under a waiver of consent.

Data sources and linkage

This study is part of a larger project using person-level linked administrative data pertaining to a cohort comprising all Western Australians aged 18+ who had a separation (discharge) from any WA hospital for any non-pregnancy-related condition, or who presented for any reason to any public hospital emergency department (ED), or who had a CT scan undertaken in WA by a public or private provider between 1 January 2003 and 31 December 2015.

Using State-based data from the WA Data Linkage System (23) and Medicare Benefits Schedule (MBS) data provided by the AIHW, cross-jurisdictional (i.e. State – Commonwealth) privacy-preserving record linkage was undertaken by the Curtin University Centre for Data Linkage (24). The following person-level linked data were available:

1) WA Cancer registry (WACR) data containing all incident cancers in the cohort (irrespective of age at diagnosis) from 1972 onwards, including diagnosis date, basis of diagnosis, site code, morphology code, behaviour type, cancer type code using the International Classification of

For peer review only

Diseases, 10th revision, Australian Modification (25) and demographics (e.g., age at diagnosis, sex, and postcode).

2) WA Hospital morbidity data collection (HMDC) records from 2003-2015 for all discharges from any WA hospital (public or private) for all conditions excluding pregnancy. It included dates and mode of admission and discharge, hospital type (tertiary or secondary), diagnosis (principal and co-diagnoses) and procedure codes.

3) WA Emergency Department Data Collection (EDDC) records 2003-2015 for all WA public hospital ED presentations. It included presentation date and time, presentation type (emergency or planned), triage code, diagnosis code and major diagnostic group.

4) WA Picture Archiving and Communication System (PACS) records for all CT scans (excluding position emission tomography (PET)/CT) 2003-2015 undertaken in all WA public tertiary hospitals and selected public secondary hospitals (limited to hospitals who do not contract out radiology services) as an out-patient, ED patient or admitted patient. It included the scan date, the type of CT examination (e.g., non-contrast Head CT etc.), and referral source.

5) MBS data capturing all CT scans subsidised by the Federal Australian Government (i.e., those performed outside of hospital or in hospital for private patients), including the date and scan type, between 2003 – 2015.

6) WA Death Registrations records from 2003-2015 including the age and date of death.

Study population

The study population was limited to patients aged 18+ years who were diagnosed with a first invasive cancer between 1 January 2004 and 31 December 2014. We excluded individuals who had a diagnosis of cancer after death or those who died within 30 days of a diagnosis of cancer in the present analysis.

For peer review only

Patient and Public Involvement

Patients were not directly involved in the study design or conduct. Our consumer representative (Mr John Stubbs) was involved in the design of the grant used to fund this research and is an ongoing member of the research team providing ongoing input to analysis of the data, interpretation of the results and development of publications. The Western Australian Data Linkage Branch and the data custodians of the WA Emergency Department Data Collection and the Picture Archiving Communications System data provided data for this project

Outcome measurement

The primary outcome was number of CT scans performed within 12 months prior and 12 months post cancer diagnosis. Multiple CT scans within the same day of the same anatomical area were counted as a single CT event (26). Similarly, we measured the number of ED presentations and hospitalisations within 12-months prior to and post cancer diagnosis. Numbers of CT scans, hospitalisations and ED presentations were captured monthly.

Covariates

Cancer types were grouped as either solid or all lymphohaematopoietic (ALH) to examine the effect of this broad classification on outcomes of interest. Demographic characteristics included sex, age group at cancer diagnosis, and Indigenous status (captured using Data Linkage WA's Derived Indigenous Flag) (27). Accessibility to services was measured using the accessibility/remoteness of index of Australia (ARIA) (28) based on postcode at the diagnosis date. Socio-economic status was classified using the socio-economic indexes for areas – index of relative socio-economic disadvantage (SEIFA-IRSD) based on postcode, for the census closest to diagnosis date categorised as quintiles (29). Secondary hospitals in the suburbs of Bentley and Armadale did not contribute data on CT scans to PACS, as they contract

For peer review only

out their radiology services to private providers, although data for CT scans from out-of-hospital and those attending tertiary hospital who live in these suburbs were provided. Hospital catchment area was stratified as 'Bentley and Armadale' and 'others' to adjust for any potential bias in the regression model. Comorbidity was determined using the Multipurpose Australian Comorbidity Scoring System (MACSS) (30)) capturing the number of 17 MACSS conditions recorded on any hospitalisation across the five years prior to cancer diagnosis. Referral sources of CT scans were grouped as ED physician, inpatient and out-of-hospital. Cancer diagnosis was also classified as either pathology or non-pathology-based diagnosis.

Statistical Analysis

Descriptive analyses were used to summarise the study population characteristics at the beginning of the study (2004), the end of the study (2014) and across the whole study period (2004-2014). After assessing the data for suitability (over-dispersion) and model fit (based on Akaike and Bayesian information criterion) multivariable negative binomial regression was performed to calculate the monthly rates of CT scanning, ED presentations, and hospitalisation 12 months prior to and 12 months post cancer diagnosis adjusted for all observed socio-demographic and clinical characteristics. Multivariable negative binomial regression was also used to examine trends in total CT scanning use, ED presentations and hospitalisations in the year prior to and after diagnosis over the study period (2004 to 2014) adjusted for all observed characteristics. Analyses used Stata SE version 15 (31).

Results

Characteristics of the study population

A total of 104,009 individuals had a first diagnosis of cancer from 2004 to 2014 (Table 1). In Table 1, the majority of the population were male (56.2%), aged 46-65 years (40.5%), classified as living in an area of least socio-economic disadvantage (31.2%), lived in major

For peer review only

cities (68.8%), and had at least one comorbidity (95.3%). In terms of cancer diagnosis, the most common type was solid cancer (91.2%) in which cancer of the male genital organs, digestive organs, melanoma and breast shared high proportions of 18.6%, 17.5%, 15.7% and 13.7% correspondingly. Up to 97% of cancer diagnoses were based on pathological methods. Both demographic and clinical characteristics were similar between the beginning (2004) and end of the study (2014).

Use of CT scanning 12-months before and after cancer diagnosis and trends over the study period

The adjusted rate of CT scanning 12 months before and after cancer diagnosis is presented in table 2. At 12 months prior to cancer diagnosis, the adjusted rate of CT scanning was 14 CT scans per 1000 patients (95%CI 13-15), and increased to 35 CT scans per 1000 patients (95%CI 33-36) at three months before diagnosis. The rate steeply increased to 294 CT scans per 1000 patients (95%CI 290-298) in the month before diagnosis and reached the highest rate of 477 CT scans per 1000 patient (95%CI 471-482) in the month of diagnosis. After cancer diagnosis, the rate of CT scanning dramatically reduced but remained elevated compared with the rate pre diagnosis (Further details of regression model output is presented in appendix A).

Figure 1 demonstrates the pattern of CT scanning 12 months before and after cancer diagnosis by two main cancer groups for two time points 2004 and 2014 for simplicity. While overall the rate of CT scanning is higher in patients diagnosed with ALH cancer than solid cancer, the patterns of CT use observed across the period 12 months pre and post cancer diagnosis were similar for both groups. When comparing the magnitude of CT rates in 2004 with those in 2014, higher rates were observed from 1 month prior to diagnosis onwards in solid cancers, whereas CT scanning rates reduced in ALH cancer.

For peer review only

Figure 2- panel A presents trends in total CT scans used in 12 months before and after diagnosis by cancer groups over the study period from 2004 to 2014. For 12 months before diagnosis, the rate of CT used in ALH cancer was consistently higher than those of solid cancers diagnosed over the entire study period. However, the rate of scans 12 months after diagnosis of ALH cancer significantly reduced between 2004 and 2009, following which the difference in the magnitude of the rate of CT scanning across the two cancer types disappeared.

Further details in Appendix A shows that patients with a higher number of comorbidities were more likely to have CT scanning with an RR 4.94 (95%CI 4.62-5.27) for those with at least six comorbidities vs those without any comorbidities. The rate of CT scanning stratified by gender, age, socio-economic, accessibility to services, hospital catchment regions, cancer diagnosis methods was slightly different as compared to the reference group in each category.

ED presentations and hospitalisation 12-month before and after cancer diagnosis and their trends over the study period

Similarly, to the pattern observed for CT scanning, as shown in Table 2 when adjusted for year of diagnosis, the rate of hospitalisation was highest in the month of diagnosis at 910 per 1,000 patients (95% CI 902-919) whereas for ED presentations the peak was in the month prior to diagnosis at 181 per 1,000 patients (95% CI 178-184). While the rate dropped in the months after diagnosis for both ED presentations and hospitalisations, it remained consistently higher than the rate before diagnosis. (Further details of regression model output is presented in appendix A).

As shown in Figure 1- panel B and C, no significant difference was observed in the rate of ED presentations or hospitalisation 12 months before cancer diagnosis in 2004 compared with 2014. However, significantly increased rates of both ED presentations and hospitalisations

For peer review only

were observed in 2014 compared with those in 2004 for the month of diagnosis and thereafter. Similar patterns were observed for both cancer groups.

In Figure 2-panel B and C, over the entire study period the rate of both hospitalisation and ED presentations for those with ALH cancer were consistently higher than those with solid cancer both before and after diagnosis with little change in rate across the year of diagnosis.

Further details in appendix A show that patients with cancer diagnosis based on pathological methods were 34% less likely to have an ED presentation compared to those with cancer diagnosis based on non-pathological methods (RR 0.66 [95%CI 0.63-0.69]) whereas no significant difference was reported for hospitalisation rate. Increasing number of comorbidities was associated with substantially higher rates of both ED presentations and hospitalisations in which the RR between those with none vs those with at least six comorbidities was highest at 5.54 (95%CI 5.15-5.96) for ED presentations and 11.5 (95%CI 10.5-12.5) for hospitalisations. The rate of ED presentations also significantly increased with higher social disadvantage and with living in regional or remote areas. Small or no differences in hospitalisation and ED presentation rates were recorded in subgroups stratified by sex, age, cancer groups and hospital catchment region.

Discussion

Our study found that the rate of CT scanning started to increase from two months before diagnosis while ED presentations and hospitalisation started to increase from one month before diagnosis. These rates peaked in the month of diagnosis then rapidly reduced within the first month after diagnosis but remained high for the next 12 months. Our results were consistent with those reported in previous studies that showed an initial increase in the use of CT scanning or ‘diagnostic investigation’ (e.g., x-ray, ultrasound, CT scan, MRI scan, angiography, endoscopies and biopsies) two months prior to cancer diagnosis (19) (21). Other studies have

For peer review only

investigated use of CT scanning in specific cancer types with a UK study finding that rates of CT use started to increase five months before a diagnosis of bladder and kidney cancer (17), and a Danish study finding an increase CT use associated with abdominal cancers diagnosed from 2014 to 2018 (18). In comparison our study evaluated changes in use of CT scanning associated with cancer diagnosis more globally and compared across a longer time period (2004-2014). The 10 year time frame means that our study period would have included more substantial changes in health care policy, cancer treatments and imaging technology that may have contributed to changes in CT (32). While it was out of the scope of this study to evaluate the incremental impact of these changes, it is worth noting the extensive changes that have occurred over this time period that have cumulatively led to the changes in CT scanning observed.

Our study is consistent with a study in NSW, Australia that reported a similar pattern on the increase in rate of ED presentation and hospitalisation before diagnosis that dramatically reduced after diagnosis, although the NSW study was limited to admissions from ED only (20). The observed increase in the use of CT scanning, ED presentations and hospitalisations in our study suggests a 'diagnostic time window' of two months whereas the increasing rate of general practice consultations have previously been found to start earlier, from three to twelve months before the diagnosis (21, 33, 34). This time disparity between patients who ultimately are diagnosed with cancer initially presenting to their doctors and CT use could reflect issues with access to CT or indicate that CT is predominantly used for staging and guiding treatment direction post initial diagnosis. It also reflects that there may be a more complex diagnostic process with many steps prior to CT use. Since our study was limited to patients with a diagnosis of cancer, we cannot comment on the use of CT for those who were ultimately found not to have cancer but where CT used to rule this out.

For peer review only

Over the whole 10 year study period, patients with an ALH cancer were more likely to have CT scans than those with a solid cancer. This may be explained by the principal role of CT scans in staging patients with ALH cancer, particularly lymphomas (35-37) while solid cancers can be diagnosed by different imaging (e.g. MRI, mammography, ultrasound, etc.), blood tests for biomarkers and biopsies that varies by cancer specific types. However, this hypothesis is not supported by our comparison of changes in CT scanning in 2004 compared with 2014, where we found that the rate of CT scanning reduced for patients with ALH cancer and increased for those with solid cancers.

Also, we observed a higher rate of ED presentation in cancer patients with higher disadvantage socioeconomic status and higher number of comorbidities that was in line with results from previous studies and reports (38-40). In contrast to our study, a NSW report recorded a lower rate of ED presentations in those living regional/remote areas as compared to those living in major cities (40). The differences may be attributable to major factors that were not adjusted in our analysis such as location of hospitals, number of GP visits, stage at cancer diagnosis and other residual confounding.

Strengths and limitations

This study included whole WA population incident cancer data linked to administrative records of health service use, which allows us to make grounded and representative conclusions for the target population based on higher statistical precision, avoidance of selection bias and negligible loss to follow up. Yet the study has several limitations. Firstly, lack of information on the indications for CT scans prevented us from determining if the CT scans were referred for cancer diagnosis or for other health issues. Therefore, our findings have been reported in terms of use of CT within 12 months before and after a diagnosis of cancer and not in terms resulting from the cancer diagnosis per se. Secondly, CT scans undertaken at two secondary

For peer review only

hospitals who contract out their radiology services are not included in the data, hence, the rate of CT use may be underestimated. However, since only a small proportion of the population reside in the two suburbs in question, and we adjusted for place of residence in the analysis (which was found to be non-significant), the missing data are likely to have minimal impact on the result.

Conclusion

Our results showed an increase in demand for health services from three months before diagnosis of cancer. While the pattern of use before and after diagnosis remained unchanged for CT, ED and hospitalisations over the study period, differences in the magnitude of use were observed. From diagnosis onwards, the magnitude of the rate of ED presentations and hospitalisations was higher in 2014 compared to the baseline year of 2004 for both cancer types. In a comparison following diagnosis the rate of CT use was significantly higher in 2014 compared to the baseline year 2004 for solid cancer, however it was lower in ALH cancers. The persisting high use of CT during post cancer diagnosis and increased CT use observed in solid cancers may warrant a further investigation of factors driving this change.

For peer review only

Funding

This study was funded with an Australian National Health and Medical Research Council grant, project grant APP1144573. The study funders had no role in the study design, conduct, manuscript writing or decision to submit for publication.

Competing interests

The authors have no conflicts of interest to declare. The institutions of RM, NH, POL, DY, CW, MB and DM received grant funding from the National Medical Research Council of Australia for investigator-initiated research. The funding agreement ensured author independence in designing the study, interpreting the data, writing, and publishing the report.

Author contributions

NTH, SK, MB, JD, DM, DY, PO, RT, & RM contributed to the study design and concept. RM, DY, and NTH contributed to the acquisition of data. RM, SK, DY, CH, MB, JD, DM, PO, CW, DM2, TC and NTH contributed to the analysis and interpretation of the data. NTH, CH, DY, SK, and RM contributed to drafting of the manuscript. RM, JD, DM, PO, DY, CW, DM2, RT and NTH contributed to critical revision of the manuscript for important intellectual content. RM and NTH contributed to statistical expertise. RM, MB, JD, DM, PO secured funding for the study.

Data sharing statement

The data that support the findings of this study are available from the relevant data custodians of the study datasets. Restrictions by the data custodians mean that the data are not publicly available or able to be provided by the authors. Researchers wishing to access the datasets used in this study should refer to the Western Australian Data Linkage Unit and the Australian Institute of Health and Welfare.

For peer review only

Ethics approval and consent to participate

Ethics approval was provided by the Curtin University Human Research Ethics Committee (HRE2017-0822), the Australian Institute of Health and Welfare Human Research Ethics Committee (EO2018/4/485), and the Western Australian Department of Health Human Research Ethics Committee (2011/97).

Patient consent

Not applicable

Acknowledgment

We thank the National Health and Medical Research Council for supporting this work. We acknowledge the Western Australian Data Linkage Unit and custodians of the Hospital Morbidity Data Collection, the WA Cancer Registry, the Emergency Department Data Collection, the WA Death registry and the WA Picture Archiving and Communications System, as well as the individuals whose data enabled this study.

For peer review only

References

1. Johnson CD, Chen M-H, Toledano AY, Heiken JP, Dachman A, Kuo MD, et al. Accuracy of CT colonography for detection of large adenomas and cancers. New England Journal of Medicine. 2008;359(12):1207-17.
2. Team NLSTR. Reduced lung-cancer mortality with low-dose computed tomographic screening. New England Journal of Medicine. 2011;365(5):395-409.
3. Malietzis G, Aziz O, Bagnall N, Johns N, Fearon K, Jenkins J. The role of body composition evaluation by computerized tomography in determining colorectal cancer treatment outcomes: a systematic review. European Journal of Surgical Oncology (EJSO). 2015;41(2):186-96.
4. DeWitt J, Devereaux BM, Lehman GA, Sherman S, Imperiale TF. Comparison of endoscopic ultrasound and computed tomography for the preoperative evaluation of pancreatic cancer: a systematic review. Clinical Gastroenterology and Hepatology. 2006;4(6):717-25.
5. De González AB, Mahesh M, Kim K-P, Bhargavan M, Lewis R, Mettler F, et al. Projected cancer risks from computed tomographic scans performed in the United States in 2007. Archives of internal medicine. 2009;169(22):2071-7.
6. Smith-Bindman R, Lipson J, Marcus R, Kim K-P, Mahesh M, Gould R, et al. Radiation dose associated with common computed tomography examinations and the associated lifetime attributable risk of cancer. Archives of internal medicine. 2009;169(22):2078-86.
7. Pearce MS, Salotti JA, Little MP, McHugh K, Lee C, Kim KP, et al. Radiation exposure from CT scans in childhood and subsequent risk of leukaemia and brain tumours: a retrospective cohort study. The Lancet. 2012;380(9840):499-505.
8. Council NR. Health risks from exposure to low levels of ionizing radiation: BEIR VII phase 2. 2006.
9. McGinnis JM, Stuckhardt L, Saunders R, Smith M. Best care at lower cost: the path to continuously learning health care in America. 2013.
10. Smith-Bindman R, Kwan ML, Marlow EC, Theis MK, Bolch W, Cheng SY, et al. Trends in use of medical imaging in US health care systems and in Ontario, Canada, 2000-2016. Jama. 2019;322(9):843-56.
11. Hess EP, Haas LR, Shah ND, Stroebel RJ, Denham CR, Swensen SJ. Trends in computed tomography utilization rates. Journal of patient safety. 2014;10(1):52-8.
12. Smith-Bindman R, Miglioretti DL, Larson EB. Rising use of diagnostic medical imaging in a large integrated health system. Health affairs. 2008;27(6):1491-502.
13. Pearce MS, Salotti JA, McHugh K, Metcalf W, Kim KP, Craft AW, et al. CT scans in young people in Northern England: trends and patterns 1993–2002. Pediatric radiology. 2011;41(7):832-8.
14. Smith-Bindman R, Miglioretti DL, Johnson E, Lee C, Feigelson HS, Flynn M, et al. Use of diagnostic imaging studies and associated radiation exposure for patients enrolled in large integrated health care systems, 1996-2010. Jama. 2012;307(22):2400-9.
15. Dinan MA, Curtis LH, Hammill BG, Patz EF, Abernethy AP, Shea AM, et al. Changes in the use and costs of diagnostic imaging among Medicare beneficiaries with cancer, 1999-2006. Jama. 2010;303(16):1625-31.
16. Maxwell S, Ha NT, Bulsara MK, Doust J, Mcrobbie D, O'Leary P, et al. Increasing use of CT requested by emergency department physicians in tertiary hospitals in Western Australia 2003–2015: an analysis of linked administrative data. BMJ open. 2021;11(3):e043315.
17. Zhou Y, Abel GA, Hamilton W, Singh H, Walter FM, Lyratzopoulos G. Imaging activity possibly signalling missed diagnostic opportunities in bladder and kidney cancer: a longitudinal data-linkage study using primary care electronic health records. Cancer Epidemiology. 2020;66:101703.
18. Jessen NH, Jensen H, Falborg AZ, Glerup H, Gronbaek H, Vedsted P. Abdominal investigations in the year preceding a diagnosis of abdominal cancer: a register-based cohort study in Denmark. Cancer epidemiology. 2021;72:101926.

For peer review only

19. Purdie S, Creighton N, White KM, Baker D, Ewald D, Lee CK, et al. Pathways to diagnosis of non-small cell lung cancer: a descriptive cohort study. NPJ primary care respiratory medicine. 2019;29(1):1-6.
20. Morrell S, Young J, Roder D. The burden of cancer on primary and secondary health care services before and after cancer diagnosis in New South Wales, Australia. BMC health services research. 2019;19(1):1-8.
21. Christensen KG, Fenger-Grøn M, Flarup KR, Vedsted P. Use of general practice, diagnostic investigations and hospital services before and after cancer diagnosis-a population-based nationwide registry study of 127,000 incident adult cancer patients. BMC health services research. 2012;12(1):1-8.
22. Benchimol EI, Smeeth L, Guttmann A, Harron K, Moher D, Petersen I, et al. The REporting of studies Conducted using Observational Routinely-collected health Data (RECORD) Statement. PLOS Medicine. 2015;12(10):e1001885.
23. Holman CD, Bass AJ, Rouse IL, Hobbs MS. Population-based linkage of health records in Western Australia: development of a health services research linked database. Australian and New Zealand journal of public health. 1999;23(5):453-9.
24. Randall SM, Ferrante AM, Boyd JH, Bauer JK, Semmens JB. Privacy-preserving record linkage on large real world datasets. J Biomed Inform. 2014;50:205-12.
25. World Health Organization. ICD-10 : international statistical classification of diseases and related health problems : tenth revision. 2nd ed ed. Geneva: World Health Organization; 2004.
26. Smith-Bindman R, Miglioretti DL, Larson EB. Rising use of diagnostic medical imaging in a large integrated health system. Health Aff (Millwood). 2008;27(6):1491-502.
27. Christensen D, Davis G, Draper G, Mitrou F, McKeown S, Lawrence D, et al. Evidence for the use of an algorithm in resolving inconsistent and missing Indigenous status in administrative data collections. Australian Journal of Social Issues. 2014;49(4):423-43.
28. Australian Bureau of Statistics. 1270.0.55.005 - Australian Statistical Geography Standard (ASGS): Volume 5 - Remoteness Structure, July 2016 2018 [updated 16/03/2018. Available from: https://www.abs.gov.au/ausstats/abs@.nsf/mf/1270.0.55.005?OpenDocument.
29. Australian Bureau of Statistics. Technical Paper - Socio-economic Indexes for Areas (SEIFA) ABS catalogue number: 2033.0.55.001. Commonwealth of Australia; 2016.
30. Holman CD, Preen DB, Baynham NJ, Finn JC, Semmens JB. A multipurpose comorbidity scoring system performed better than the Charlson index. Journal of clinical epidemiology. 2005;58(10):1006-14.
31. StataCorp. Stata Statistical Software: Release 15. College Station, TX: StataCorp LLC; 2017.
32. Wright CM, Bulsara MK, Norman R, Moorin RE. Increase in computed tomography in Australia driven mainly by practice change: a decomposition analysis. Health Policy. 2017;121(7):823-9.
33. Hansen PL, Hjertholm P, Vedsted P. Increased diagnostic activity in general practice during the year preceding colorectal cancer diagnosis. International Journal of Cancer. 2015;137(3):615-24.
34. Guldbrandt LM, Møller H, Jakobsen E, Vedsted P. General practice consultations, diagnostic investigations, and prescriptions in the year preceding a lung cancer diagnosis. Cancer medicine. 2017;6(1):79-88.
35. Armitage JO, Gascoyne RD, Lunning MA, Cavalli F. Non-hodgkin lymphoma. The lancet. 2017;390(10091):298-310.
36. Connors JM. State-of-the-art therapeutics: Hodgkin's lymphoma. Journal of clinical oncology. 2005;23(26):6400-8.
37. Vinnicombe SJ, Reznek RH. Computerised tomography in the staging of Hodgkin's disease and non-Hodgkin's lymphoma. European journal of nuclear medicine and molecular imaging. 2003;30(1):S42-S55.
38. Wong TH, Lau ZY, Ong WS, Tan KB, Wong YJ, Farid M, et al. Cancer patients as frequent attenders in emergency departments: a national cohort study. Cancer medicine. 2018;7(9):4434-46.

For peer review only

39. Unwin M, Crisp E, Stankovich J, McCann D, Kinsman L. Socioeconomic disadvantage as a driver of non-urgent emergency department presentations: a retrospective data analysis. PLoS One. 2020;15(4):e0231429.
40. Bureau of Health Information. The Insights Series: Emergency department utilisation by people with cancer

Sydney (NSW); BHI; 2014.

For peer review only

List of tables, Figures and Appendix

Tables

Table 1. Characteristics of the Study Population

Table 2. Adjusted rate of CT scanning, ED presentations and Hospitalisations in 12 months before and after cancer diagnosis

Figures

Figure 1. Adjusted rate of CT scanning, ED presentations and hospitalisation in months before and after diagnosis by cancer groups in 2004 and 2014

Figure 2. Adjusted rate of total CT scans, ED presentations and hospitalisations in a year before and after cancer diagnosis over study period by cancer groups

Appendices

Appendix A. Multivariable negative binomial regression of monthly use of CT scanning, ED presentation, and hospitalisations within 12 Months before and after Cancer Diagnosis over the study period.

Appendix B. Multivariable negative binomial regression of total use of CT scanning, ED presentation, and hospitalisations in a year before diagnosis with cancer over the study period

Appendix C. Multivariable negative binomial regression of total use of CT scanning, ED presentation, and hospitalisations in a year after diagnosis with cancer over the study period

For peer review only

For peer review only

1 **Table 1. Characteristics of the Study Population**

Characteristics		Year of cancer diagnosis 2004 n=8,081		2014 n = 10,884		2004-2014 (overall) n=104,009	
		n	%	*n*	%	*n*	%
**Sex**	Male	4,468	55.29%	5,977	54.92%	58,411	56.16%
	Female	3,613	44.71%	4,907	45.08%	45,598	43.84%
**Age group at diagnosis**	18-45 years	909	11.25%	1,106	10.16%	10,827	10.41%
	46-65 years	3,243	40.13%	4,283	39.35%	42,104	40.48%
	66-75 years	2,008	24.85%	2,980	27.38%	26,142	25.13%
	>75 years	1,921	23.77%	2,515	23.11%	24,936	23.97%
**Socio-economic status**†	Least disadvantage	2,419	29.93%	2,939	27.00%	32,464	31.21%
	Less disadvantage	1,224	15.15%	1,673	15.37%	15,295	14.71%
	Moderate disadvantage	1,939	23.99%	1,972	18.12%	21,954	21.11%
	High disadvantage	1,664	20.59%	2,941	27.02%	23,913	22.99%
	Highest disadvantage	764	9.45%	1,351	12.41%	9,893	9.51%
	Unknown	71	0.88%	8	0.07%	490	0.47%
**Accessibility to services**‡	Major cities	5,286	65.41%	7,902	72.60%	71,574	68.82%
	Inner regional	1,684	20.84%	1473	13.55%	17,646	16.97%
	Outer regional	751	9.29%	964	8.86%	9,623	9.25%
	Remote	98	1.21%	244	2.24%	1,454	1.40%
	Very remote	250	3.09%	#	#	3,535	3.40%
	Unknown#	12	0.15%	#	#	177	0.17%
**Bases of diagnosis**	Non-pathological	249	3.12%	360	3.35%	3,413	3.32%
	Pathological	7,738	96.88%	10,398	96.65%	99,536	96.68%
**Hospital catchment area**	Other	6,742	83.43%	9,142	83.99%	87,384	84.02%
	Bentley and Armadale	1,339	16.57%	1,742	16.01%	16,625	15.98%
**Number of comorbidities**§	None	746	9.23%	383	3.52%	4,854	4.67%
	1-2	2,502	30.96%	1,856	17.05%	22,136	21.28%

For peer review only

Characteristics		Year of cancer diagnosis: 2004 n=8,081		2014 n = 10,884		2004-2014 (overall) n=104,009	
		n	%	*n*	%	*n*	%
	3-5	2,773	34.32%	3,830	35.19%	37,740	36.29%
	6+	2,060	25.49%	4,815	44.24%	39,279	37.77%
**Solid cancer**	Cancer of the male genital organs	1,436	17.77%	1,830	16.81%	19,353	18.61%
	Cancer of the digestive organs	1,438	17.79%	1,753	16.11%	18,222	17.52%
	Melanoma	1,280	15.84%	1,891	17.37%	16,292	15.66%
	Breast cancer	1,087	13.45%	1,630	14.98%	14,262	13.71%
	Cancer of the respiratory organs	679	8.40%	875	8.04%	8,381	8.06%
	Cancer of the urinary tract	322	3.98%	511	4.69%	4,541	4.37%
	Cancer of the female genital organs	348	4.31%	438	4.02%	4,130	3.97%
	Mouth and pharynx cancer	229	2.83%	322	2.96%	3,049	2.93%
	Thyroid cancer	148	1.83%	238	2.19%	2,147	2.06%
	Brain cancer	111	1.37%	158	1.45%	1,523	1.46%
	Cancer of the soft tissues	114	1.41%	153	1.41%	1,459	1.40%
	Cancer of unspecified sites	121	1.50%	112	1.03%	1,318	1.27%
	Bone cancer	9	0.11%	12	0.11%	157	0.15%
	All Solid	7,322	90.61%	9,923	91.17%	94,834	91.18%
**All haematopoietic cancer**§	Leukaemias and myelodysplasias	327	4.05%	330	3.03%	3,676	3.53%
	Other lymphomas	203	2.51%	292	2.68%	2,742	2.64%
	Other lymphoid cancers	187	2.31%	289	2.66%	2,280	2.19%
	Hodgkin's lymphoma	42	0.52%	50	0.46%	477	0.46%
	All lymphoid and haematopoietic cancer	759	9.39%	961	8.83%	9,175	8.82%

†Socio-economic status measured by SEIFA-IRSD: Socio-Economic Indexes for Areas, Index of Relative Socio-Economic Disadvantage.
‡Accessibility to services was measured using ARIA: Accessibility and Remoteness Index of Australia.
§ Comorbidity: Number of comorbid conditions reported in hospitalisation data in the 5 years prior to the date of cancer diagnosis
¶ED presentations: Emergency department presentations

For peer review only

[#]Where small cells are suppressed, random adjustments have been made to other cells within different levels of the same variable to prevent calculation of suppressed cell counts

Note. Indigenous status cannot be reported as per restrictions imposed by the human research ethics approval and can only be used for model adjustment purposes. 12 months after the diagnosis is inclusive of the date of the diagnosis.

For peer review only

Table 2. Adjusted rate of CT scanning, ED presentations and Hospitalisations in 12 months before and after cancer diagnosis

Month before and after cancer diagnosis	CT scanning		Hospitalisations		ED presentations	
	Rate/1000 patients	95%CI	Rate/1000 patients	95%CI	Rate/1000 patients	95%CI
-12	14	(13; 15)	48	(46; 50)	26	(25; 27)
-11	14	(13; 15)	51	(48; 53)	27	(25; 28)
-10	14	(13; 15)	51	(49; 53)	27	(26; 28)
-9	15	(14; 15)	52	(49; 54)	28	(26; 29)
-8	15	(15; 16)	52	(50; 55)	28	(27; 29)
-7	16	(15; 17)	56	(53; 58)	29	(28; 30)
-6	18	(17; 19)	56	(53; 58)	30	(29; 31)
-5	20	(19; 21)	58	(56; 61)	32	(31; 33)
-4	24	(23; 25)	64	(62; 67)	37	(35; 38)
-3	35	(33; 36)	73	(70; 75)	43	(42; 45)
-2	69	(67; 71)	94	(91; 96)	59	(58; 61)
-1	294	(290; 298)	371	(367; 375)	181	(178; 184)
1	477	(471; 482)	910	(902; 919)	125	(123; 128)
2	194	(190; 197)	624	(617; 631)	104	(102; 106)
3	150	(146; 153)	636	(629; 644)	94	(91; 96)
4	133	(130; 136)	587	(580; 595)	84	(82; 87)
5	120	(117; 123)	502	(496; 509)	75	(73; 77)
6	119	(116; 122)	431	(425; 438)	70	(68; 72)
7	119	(116; 122)	369	(363; 375)	63	(61; 65)
8	111	(108; 114)	312	(306; 318)	61	(59; 63)
9	100	(97; 103)	259	(254; 264)	57	(55; 58)
10	95	(92; 98)	236	(231; 241)	56	(54; 58)
11	93	(90; 96)	223	(218; 228)	53	(51; 55)
12	95	(92; 97)	214	(209; 219)	52	(50; 54)

CT: Computed tomography, ED: emergency department, CI: confidence interval.

Notes: full model with adjusted covariates is presented in appendix A.

For peer review only

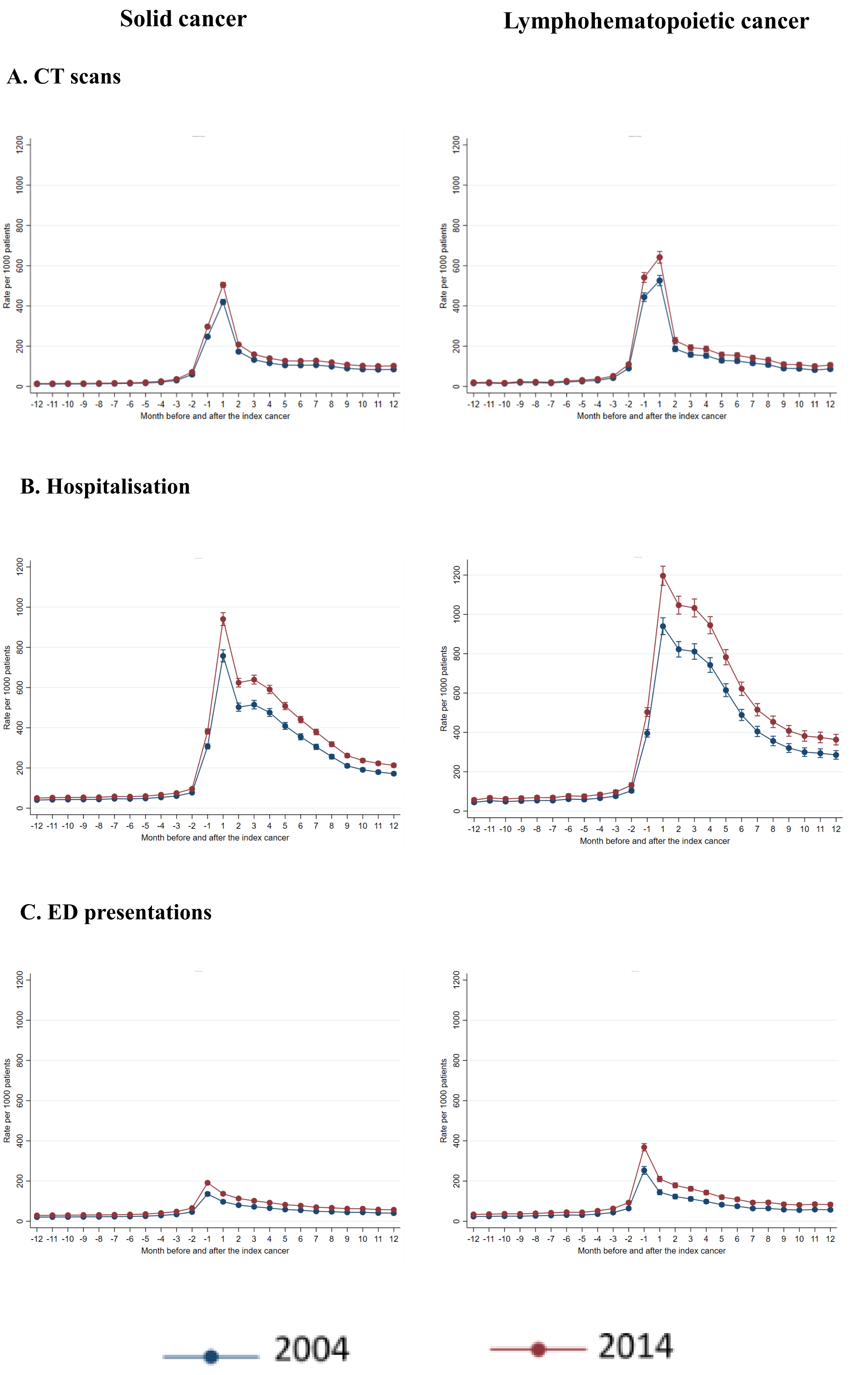

Figure 1. Adjusted rate of CT scanning, hospitalisation and ED presentations in months before and after diagnosis by cancer groups in 2004 and 2014

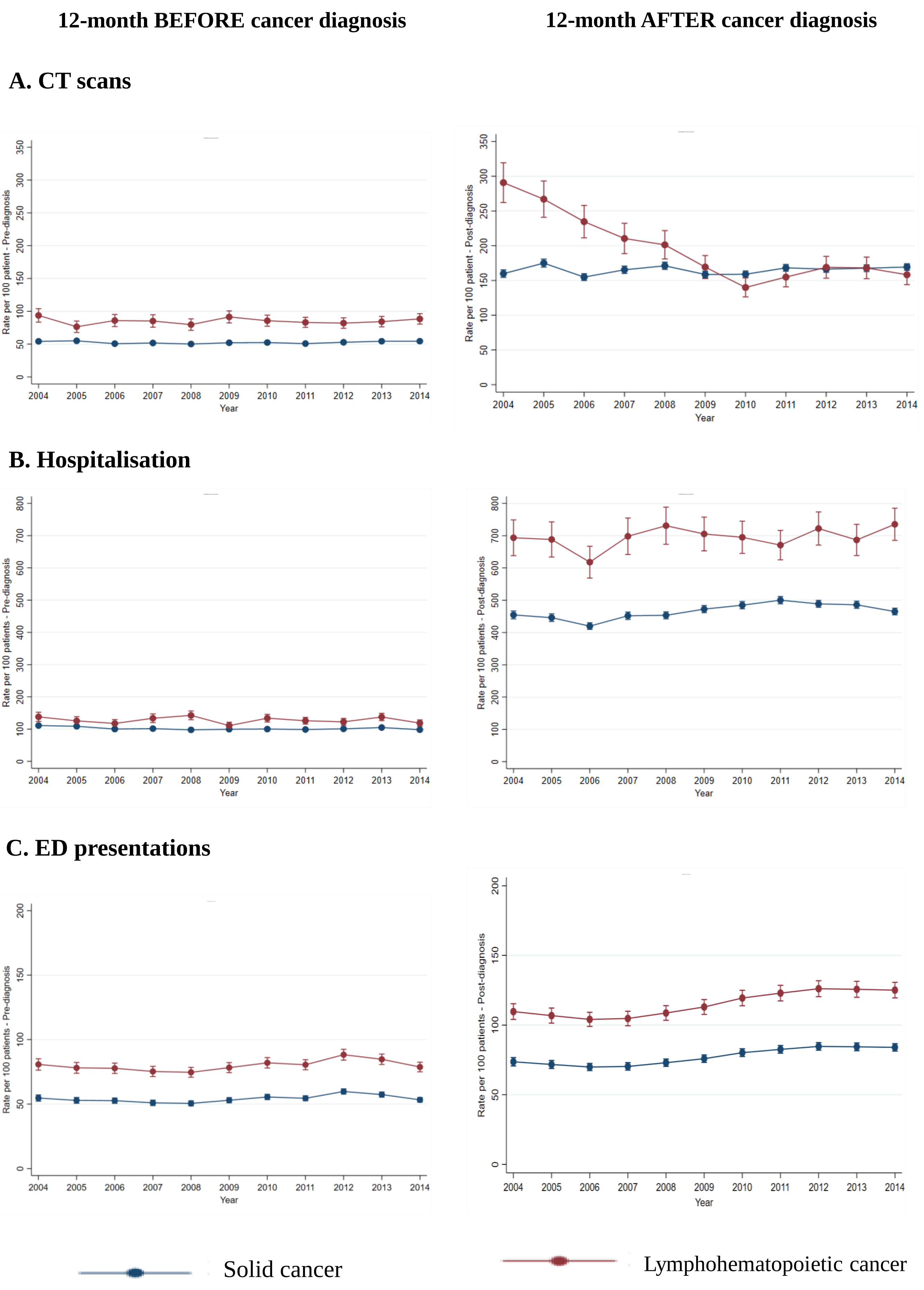

Figure 2. Adjusted rate of total CT scans, hospitalisations and ED presentations in a year before and after cancer diagnosis over study period by cancer groups

Appendix

The use of computed tomography, ED presentation and hospitalisations 12-months before and after a diagnosis of cancer in Western Australia: A population-based retrospective cohort study

Author information

Ninh T. Ha[1], PhD, thi.ha@curtin.edu.au

Sviatlana Kamarova[1], PhD, sviatlana.kamarova@curtin.edu.au

David Youens[1], david.youens@curtin.edu.au

Chau Ho[1,] chau.ho@curtin.edu.au

Max Bulsara [2,3], PhD, max.bulsara@nd.edu.au

Jenny Doust[4], BMBS, PhD, j.doust@uq.edu.au

Donald McRobbie[5], PhD, donald.mcrobbie@adelaide.edu.au

Peter O'Leary [1,6,7], PhD, peter.oleary@health.wa.gov.au

Cameron Wright[1,8,9,10], MD, cameron.wright@curtin.edu.au

Richard Trevithick[11], MD, richard.trevithick@health.wa.gov.au

Rachael Moorin,[1,3] PhD, R.Moorin@curtin.edu.au

Affiliations

1. Health Economics and Data Analytics, Curtin School of Population Health, Faculty of Health Sciences, Curtin University, Western Australia;
2. Institute for Health Research, University of Notre Dame, Western Australia;

For peer review only

3. Centre for Health Services Research, School of Population and Global Health, The University of Western Australia;

4. Centre for Longitudinal and Life Course Research, Faculty of Medicine, University of Queensland;

5. School of Physical Sciences, University of Adelaide;

6. Obstetrics and Gynaecology Medical School, Faculty of Health and Medical Sciences, The University of Western Australia, Perth, Western Australia, Australia;

7. PathWest Laboratory Medicine, QE2 Medical Centre, Nedlands, Western Australia, Australia;

8. Fiona Stanley Hospital, 11 Robin Warren Dr, Murdoch, Western Australia, Australia;

9. Division of Internal Medicine, Medical School, Faculty of Health and Medical Sciences, The University of Western Australia;

10. School of Medicine, College of Health and Medicine, University of Tasmania, Hobart, Tasmania;

11. Western Australian Cancer Registry, Clinical Excellence Division, Department of Health, East Perth, Western Australia.

***Corresponding author:**

Ninh Thi Ha

Health Economics & Data Analytics, Curtin School of Population Health, Faculty of Health Sciences, Curtin University

GPO Box U1987, Perth, Western Australia, 6845

Email: Thi.Ha@curtin.edu.au; ninh.ha05@gmail.com

For peer review only

Phone: +61 08 9266 5134

For peer review only

Appendix A. Multivariable negative binomial regression of monthly use of CT scanning, ED presentation, and hospitalisations within 12 Months before and after Cancer Diagnosis over the study period.

	CT scan		Hospitalisations		ED presentations	
	RR	95%CI	RR	95%CI	RR	95%CI
**Month pre& post cancer diagnosis**						
-12	1	(1; 1)	1	(1; 1)	1	(1; 1)
-11	1	(0.92; 1.09)	1.04*	(1.00; 1.08)	1.03	(0.97; 1.10)
-10	1.04	(0.96; 1.14)	1.05**	(1.01; 1.10)	1.04	(0.98; 1.10)
-9	1.04	(0.95; 1.13)	1.07**	(1.02; 1.11)	1.07*	(1.01; 1.14)
-8	1.12**	(1.03; 1.21)	1.07***	(1.03; 1.12)	1.09**	(1.02; 1.15)
-7	1.17***	(1.08; 1.28)	1.16***	(1.11; 1.21)	1.12***	(1.05; 1.19)
-6	1.27***	(1.17; 1.37)	1.14***	(1.09; 1.19)	1.16***	(1.09; 1.23)
-5	1.40***	(1.29; 1.52)	1.19***	(1.14; 1.25)	1.24***	(1.17; 1.31)
-4	1.75***	(1.62; 1.89)	1.32***	(1.27; 1.38)	1.41***	(1.33; 1.50)
-3	2.52***	(2.34; 2.71)	1.49***	(1.43; 1.55)	1.66***	(1.57; 1.76)
-2	4.94***	(4.61; 5.28)	1.91***	(1.82; 1.99)	2.26***	(2.14; 2.39)
-1	20.6***	(19.3; 21.9)	7.57***	(7.22; 7.94)	6.62***	(6.30; 6.95)
1	34.9***	(32.8; 37.2)	18.7***	(17.8; 19.6)	4.74***	(4.51; 4.98)
2	14.4***	(13.5; 15.4)	12.4***	(11.8; 13.0)	3.92***	(3.72; 4.12)
3	11.0***	(10.3; 11.8)	12.7***	(12.1; 13.3)	3.52***	(3.35; 3.71)
4	9.67***	(9.05; 10.3)	11.7***	(11.2; 12.3)	3.19***	(3.03; 3.36)
5	8.79***	(8.22; 9.39)	10.1***	(9.61; 10.6)	2.85***	(2.70; 3.01)
6	8.75***	(8.19; 9.36)	8.75***	(8.33; 9.18)	2.68***	(2.54; 2.83)
7	8.85***	(8.28; 9.46)	7.52***	(7.16; 7.89)	2.41***	(2.28; 2.55)
8	8.26***	(7.73; 8.83)	6.31***	(6.01; 6.63)	2.31***	(2.19; 2.45)
9	7.48***	(6.99; 8.01)	5.19***	(4.95; 5.45)	2.17***	(2.05; 2.30)
10	7.10***	(6.63; 7.59)	4.70***	(4.48; 4.94)	2.16***	(2.04; 2.29)
11	6.99***	(6.53; 7.48)	4.42***	(4.21; 4.65)	2.01***	(1.90; 2.13)
12	7.07***	(6.61; 7.57)	4.23***	(4.03; 4.44)	1.97***	(1.86; 2.09)
**Cancer group**						
Solid cancer	1	(1; 1)	1	(1; 1)	1	(1; 1)
ALH cancer†	1.25*	(1.02; 1.53)	0.99	(0.84; 1.17)	1.02	(0.87; 1.20)
**Interaction term of months # cancer groups**						
-12 # solid cancer	1	(1; 1)	1	(1; 1)	1	(1; 1)
-12 # ALH	1	(1; 1)	1	(1; 1)	1	(1; 1)
-11 # solid cancer	1	(1; 1)	1	(1; 1)	1	(1; 1)
-11 # ALH	1.02	(0.77; 1.34)	1.14*	(1.02; 1.29)	1	(0.82; 1.22)
-10 # solid cancer	1	(1; 1)	1	(1; 1)	1	(1; 1)
-10 # ALH	0.85	(0.64; 1.11)	1.04	(0.92; 1.16)	1.05	(0.87; 1.27)
-9 # solid cancer	1	(1; 1)	1	(1; 1)	1	(1; 1)
-9 # ALH	1.14	(0.88; 1.48)	1.09	(0.97; 1.22)	1	(0.83; 1.21)
-8 # solid cancer	1	(1; 1)	1	(1; 1)	1	(1; 1)
-8 # ALH	1.02	(0.78; 1.32)	1.13*	(1.00; 1.27)	1.07	(0.89; 1.28)

For peer review only

	CT scan		Hospitalisations		ED presentations	
	RR	95%CI	RR	95%CI	RR	95%CI
-7 # solid cancer	1	(1; 1)	1	(1; 1)	1	(1; 1)
-7 # ALH	0.88	(0.67; 1.14)	1.04	(0.92; 1.18)	1.11	(0.91; 1.35)
-6 # solid cancer	1	(1; 1)	1	(1; 1)	1	(1; 1)
-6 # ALH	1.08	(0.84; 1.38)	1.20**	(1.06; 1.35)	1.14	(0.95; 1.38)
-5 # solid cancer	1	(1; 1)	1	(1; 1)	1	(1; 1)
-5 # ALH	1.11	(0.87; 1.43)	1.11	(0.98; 1.26)	1.05	(0.87; 1.28)
-4 # solid cancer	1	(1; 1)	1	(1; 1)	1	(1; 1)
-4 # ALH	1.04	(0.82; 1.33)	1.12	(0.98; 1.27)	1.08	(0.90; 1.31)
-3 # solid cancer	1	(1; 1)	1	(1; 1)	1	(1; 1)
-3 # ALH	1.02	(0.81; 1.30)	1.15*	(1.01; 1.31)	1.12	(0.94; 1.33)
-2 # solid cancer	1	(1; 1)	1	(1; 1)	1	(1; 1)
-2 # ALH	1.11	(0.89; 1.38)	1.22**	(1.06; 1.40)	1.21*	(1.02; 1.43)
-1 # solid cancer	1	(1; 1)	1	(1; 1)	1	(1; 1)
-1 # ALH	1.31**	(1.07; 1.61)	1.17*	(1.00; 1.38)	1.63***	(1.39; 1.92)
1 # solid cancer	1	(1; 1)	1	(1; 1)	1	(1; 1)
1 # ALH	0.92	(0.74; 1.13)	1.13	(0.96; 1.34)	1.30**	(1.11; 1.54)
2 # solid cancer	1	(1; 1)	1	(1; 1)	1	(1; 1)
2 # ALH	0.79*	(0.64; 0.97)	1.49***	(1.26; 1.76)	1.34***	(1.13; 1.59)
3 # solid cancer	1	(1; 1)	1	(1; 1)	1	(1; 1)
3 # ALH	0.87	(0.70; 1.08)	1.44***	(1.22; 1.70)	1.35***	(1.14; 1.60)
4 # solid cancer	1	(1; 1)	1	(1; 1)	1	(1; 1)
4 # ALH	0.96	(0.77; 1.19)	1.42***	(1.21; 1.68)	1.31**	(1.11; 1.56)
5 # solid cancer	1	(1; 1)	1	(1; 1)	1	(1; 1)
5 # ALH	0.89	(0.72; 1.11)	1.37***	(1.16; 1.62)	1.23*	(1.04; 1.47)
6 # solid cancer	1	(1; 1)	1	(1; 1)	1	(1; 1)
6 # ALH	0.88	(0.70; 1.09)	1.26**	(1.06; 1.48)	1.19*	(1.00; 1.42)
7 # solid cancer	1	(1; 1)	1	(1; 1)	1	(1; 1)
7 # ALH	0.80*	(0.64; 0.99)	1.21*	(1.02; 1.43)	1.13	(0.95; 1.35)
8 # solid cancer	1	(1; 1)	1	(1; 1)	1	(1; 1)
8 # ALH	0.79*	(0.63; 0.99)	1.27**	(1.07; 1.50)	1.19	(0.99; 1.42)
9 # solid cancer	1	(1; 1)	1	(1; 1)	1	(1; 1)
9 # ALH	0.73**	(0.58; 0.91)	1.39***	(1.17; 1.64)	1.14	(0.95; 1.37)
10 # solid cancer	1	(1; 1)	1	(1; 1)	1	(1; 1)
10 # ALH	0.76*	(0.60; 0.95)	1.43***	(1.21; 1.70)	1.1	(0.92; 1.33)
11 # solid cancer	1	(1; 1)	1	(1; 1)	1	(1; 1)
11 # ALH	0.71**	(0.57; 0.90)	1.49***	(1.26; 1.77)	1.24*	(1.03; 1.49)
12 # solid cancer	1	(1; 1)	1	(1; 1)	1	(1; 1)
12 # ALH	0.75*	(0.59; 0.94)	1.52***	(1.28; 1.80)	1.23*	(1.03; 1.49)
**Year of cancer diagnosis**						
2004	1	(1; 1)	1	(1; 1)	1	(1; 1)
2005	1.05*	(1.00; 1.09)	0.98	(0.93; 1.04)	0.96	(0.89; 1.04)
2006	0.94**	(0.90; 0.98)	0.91**	(0.86; 0.96)	0.94	(0.87; 1.02)
2007	0.96	(0.93; 1.00)	0.98	(0.92; 1.03)	0.93	(0.86; 1.00)
2008	0.97	(0.93; 1.00)	0.97	(0.91; 1.02)	0.94	(0.87; 1.01)

For peer review only

	CT scan		Hospitalisations		ED presentations	
	**RR**	**95%CI**	**RR**	**95%CI**	**RR**	**95%CI**
2009	0.92***	(0.88; 0.95)	0.99	(0.94; 1.05)	0.98	(0.91; 1.06)
2010	0.91***	(0.88; 0.95)	1.02	(0.96; 1.07)	1.03	(0.95; 1.11)
2011	0.94**	(0.90; 0.97)	1.02	(0.97; 1.07)	1.04	(0.96; 1.12)
2012	0.95**	(0.92; 0.99)	1.02	(0.97; 1.08)	1.09*	(1.01; 1.18)
2013	0.96*	(0.93; 1.00)	1.03	(0.97; 1.09)	1.07	(0.99; 1.16)
2014	0.96	(0.93; 1.00)	0.97	(0.92; 1.03)	1.04	(0.96; 1.12)
**Sex**						
Male	1	(1; 1)	1	(1; 1)	1	(1; 1)
Female	1	(0.99; 1.02)	1.13***	(1.11; 1.16)	0.98*	(0.96; 1.00)
**Age- group at diagnosis**						
18-45 years	1	(1; 1)	1	(1; 1)	1	(1; 1)
46-65 years	1.02	(0.99; 1.05)	0.96**	(0.93; 0.99)	0.76***	(0.73; 0.79)
66-75 years	0.96**	(0.93; 0.99)	0.84***	(0.81; 0.87)	0.73***	(0.70; 0.76)
>75 years	0.74***	(0.72; 0.77)	0.66***	(0.63; 0.68)	0.84***	(0.80; 0.87)
**Socio-economic status**‡						
Least disadvantage	1	(1; 1)	1	(1; 1)	1	(1; 1)
Less disadvantage	1.08***	(1.05; 1.11)	1.01	(0.98; 1.05)	1.30***	(1.26; 1.35)
Moderate disadvantage	1.07***	(1.05; 1.10)	1.01	(0.98; 1.04)	1.45***	(1.41; 1.49)
High disadvantage	1.12***	(1.10; 1.15)	1.01	(0.98; 1.05)	1.65***	(1.60; 1.69)
Highest disadvantage	1.12***	(1.08; 1.15)	0.99	(0.94; 1.03)	1.75***	(1.67; 1.84)
Unknown	1.1	(0.97; 1.26)	1.06	(0.81; 1.40)	1.12	(0.94; 1.33)
**Accessibility to services**§						
Major cities	1	(1; 1)	1	(1; 1)	1	(1; 1)
Inner regional	0.87***	(0.85; 0.89)	0.96**	(0.93; 0.99)	1.19***	(1.15; 1.22)
Outer regional	0.84***	(0.81; 0.86)	0.91***	(0.87; 0.94)	1.52***	(1.47; 1.58)
Remote	0.84***	(0.79; 0.91)	0.83***	(0.78; 0.88)	1.69***	(1.58; 1.81)
Very remote	0.87***	(0.83; 0.91)	0.83***	(0.78; 0.89)	2.33***	(2.21; 2.47)
Unknown	0.96	(0.75; 1.23)	0.67**	(0.50; 0.90)	1.03	(0.76; 1.39)
**Comorbidities**¶						
0	1	(1; 1)	1	(1; 1)	1	(1; 1)
1-2	1.87***	(1.75; 2.00)	3.74***	(3.44; 4.06)	1.19***	(1.11; 1.29)
3-5	3.00***	(2.81; 3.21)	6.58***	(6.06; 7.15)	2.23***	(2.07; 2.40)
6+	4.94***	(4.62; 5.27)	11.5***	(10.5; 12.5)	5.54***	(5.15; 5.96)
**Basis of diagnosis**						
Non-pathological	1	(1; 1)	1	(1; 1)	1	(1; 1)
Pathological	0.95*	(0.91; 0.99)	1.08	(0.96; 1.20)	0.66***	(0.63; 0.69)
**Hospital catchment region**						
Other	1	(1; 1)	1	(1; 1)	1	(1; 1)
Bentley and Armadale	1.04***	(1.02; 1.07)	1.01	(0.98; 1.05)	0.99	(0.95; 1.03)

* p <0.05, ** p <0.01, *** p <0.001

For peer review only

CT: Computed tomography, ED: emergency department, IRR: incidence rate ratio, ALH: All haematopoietic cancer.
RRs were also adjusted for interactions between time in months before and after cancer diagnosis and cancer groups; time in years of cancer diagnosis cohort and cancer groups; and Indigenous status. Please note Indigenous status cannot be reported as per restrictions imposed by the human research ethics approval and can only be used for model adjustment purposes. Month post diagnosis = 1 includes the day diagnosed.

For peer review only

Appendix B. Multivariable negative binomial regression of total use of CT scanning, ED presentation, and hospitalisations in a year before diagnosis with cancer over the study period

	CT scan		Hospitalisations		ED presentations	
	RR	95%CI	RR	95%CI	RR	95%CI
**Cancer group**						
Solid cancer	1	(1; 1)	1	(1; 1)	1	(1; 1)
ALH cancer	1.56***	(1.39; 1.75)	1.07	(0.96; 1.19)	1.31***	(1.14; 1.50)
**Year of cancer diagnosis**						
2004	1	(1; 1)	1	(1; 1)	1	(1; 1)
2005	1.01	(0.96; 1.07)	0.98	(0.93; 1.03)	0.97	(0.91; 1.04)
2006	0.93*	(0.88; 0.99)	0.90***	(0.86; 0.95)	0.96	(0.90; 1.02)
2007	0.95	(0.90; 1.01)	0.91***	(0.87; 0.96)	0.93*	(0.88; 1.00)
2008	0.92**	(0.88; 0.98)	0.88***	(0.84; 0.92)	0.93*	(0.87; 0.99)
2009	0.96	(0.91; 1.01)	0.90***	(0.86; 0.94)	0.97	(0.92; 1.04)
2010	0.97	(0.92; 1.02)	0.90***	(0.86; 0.94)	1.02	(0.96; 1.09)
2011	0.94*	(0.89; 0.99)	0.89***	(0.85; 0.93)	1.00	(0.94; 1.06)
2012	0.97	(0.92; 1.03)	0.91***	(0.87; 0.95)	1.10**	(1.04; 1.16)
2013	1.00	(0.95; 1.06)	0.94*	(0.90; 0.99)	1.05	(0.99; 1.11)
2014	1.00	(0.95; 1.06)	0.88***	(0.85; 0.92)	0.97	(0.91; 1.03)
**Interaction term of cancer diagnosis year and cancer type**						
2004 # solid cancer	1	(1; 1)	1	(1; 1)	1	(1; 1)
2004 # ALH	1	(1; 1)	1	(1; 1)	1	(1; 1)
2005 # solid cancer	1	(1; 1)	1	(1; 1)	1	(1; 1)
2005 # ALH	0.80*	(0.68; 0.95)	0.93	(0.80; 1.08)	0.93	(0.77; 1.13)
2006 # solid cancer	1	(1; 1)	1	(1; 1)	1	(1; 1)
2006 # ALH	0.98	(0.83; 1.16)	0.94	(0.81; 1.10)	1.03	(0.85; 1.25)
2007 # solid cancer	1	(1; 1)	1	(1; 1)	1	(1; 1)
2007 # ALH	0.95	(0.81; 1.12)	1.06	(0.91; 1.23)	0.98	(0.81; 1.19)
2008 # solid cancer	1	(1; 1)	1	(1; 1)	1	(1; 1)
2008 # ALH	0.92	(0.78; 1.08)	1.18*	(1.01; 1.36)	0.95	(0.79; 1.15)
2009 # solid cancer	1	(1; 1)	1	(1; 1)	1	(1; 1)
2009 # ALH	1.02	(0.87; 1.19)	0.90	(0.77; 1.04)	0.95	(0.79; 1.14)
2010 # solid cancer	1	(1; 1)	1	(1; 1)	1	(1; 1)
2010 # ALH	0.95	(0.81; 1.11)	1.08	(0.93; 1.24)	0.94	(0.78; 1.12)
2011 # solid cancer	1	(1; 1)	1	(1; 1)	1	(1; 1)
2011 # ALH	0.95	(0.81; 1.10)	1.03	(0.89; 1.18)	0.97	(0.82; 1.16)
2012 # solid cancer	1	(1; 1)	1	(1; 1)	1	(1; 1)
2012 # ALH	0.90	(0.77; 1.05)	0.98	(0.85; 1.13)	0.96	(0.80; 1.14)
2013 # solid cancer	1	(1; 1)	1	(1; 1)	1	(1; 1)
2013 # ALH	0.90	(0.77; 1.05)	1.06	(0.92; 1.21)	1.02	(0.86; 1.22)
2014 # solid cancer	1	(1; 1)	1	(1; 1)	1	(1; 1)

For peer review only

	CT scan		Hospitalisations		ED presentations	
	RR	95%CI	RR	95%CI	RR	95%CI
2014 # ALH	0.94	(0.81; 1.09)	0.97	(0.85; 1.12)	1.07	(0.90; 1.27)
**Sex**						
Male	1	(1; 1)	1	(1; 1)	1	(1; 1)
Female	0.94***	(0.92; 0.96)	0.78***	(0.77; 0.79)	0.92***	(0.90; 0.94)
**Age- group at diagnosis**						
18-45 years	1	(1; 1)	1	(1; 1)	1	(1; 1)
46-65 years	1.00	(0.96; 1.04)	1.05**	(1.01; 1.09)	0.70***	(0.68; 0.73)
66-75 years	1.07**	(1.02; 1.11)	1.25***	(1.21; 1.30)	0.68***	(0.65; 0.71)
>75 years	0.94**	(0.91; 0.98)	1.38***	(1.34; 1.44)	0.86***	(0.82; 0.89)
**Socio-economic status**						
Least disadvantage	1	(1; 1)	1	(1; 1)	1	(1; 1)
Less disadvantage	1.07***	(1.03; 1.10)	1.03*	(1.01; 1.06)	1.28***	(1.23; 1.33)
Moderate disadvantage	1.09***	(1.06; 1.12)	1.04**	(1.01; 1.07)	1.43***	(1.38; 1.48)
High disadvantage	1.15***	(1.11; 1.18)	1.04***	(1.02; 1.07)	1.60***	(1.55; 1.66)
Highest disadvantage	1.15***	(1.10; 1.19)	1.01	(0.98; 1.04)	1.72***	(1.65; 1.79)
Unknown	1.22*	(1.01; 1.47)	1.53***	(1.31; 1.77)	0.91	(0.72; 1.14)
**Accessibility to services**						
Major cities	1	(1; 1)	1	(1; 1)	1	(1; 1)
Inner regional	0.84***	(0.82; 0.87)	0.97*	(0.95; 1.00)	1.12***	(1.09; 1.16)
Outer regional	0.81***	(0.77; 0.84)	0.92***	(0.89; 0.96)	1.46***	(1.40; 1.52)
Remote	0.75***	(0.69; 0.83)	0.84***	(0.77; 0.91)	1.69***	(1.55; 1.84)
Very remote	0.78***	(0.74; 0.84)	0.85***	(0.81; 0.90)	2.49***	(2.35; 2.63)
Unknown	0.86	(0.62; 1.19)	0.41***	(0.30; 0.58)	1.10	(0.74; 1.64)
**Number of comorbidities**						
0	1	(1; 1)	1	(1; 1)	1	(1; 1)
1-2	2.28***	(2.08; 2.49)	180.9***	(90.4; 362.2)	1.20***	(1.10; 1.31)
3-5	3.93***	(3.60; 4.29)	430.7***	(215.2; 861.7)	2.62***	(2.41; 2.85)
6+	6.76***	(6.20; 7.37)	1026.5***	(513.0; 2053.8)	6.74***	(6.21; 7.32)
**Basis of diagnosis**						
Non-pathological	1	(1; 1)	1	(1; 1)	1	(1; 1)
Pathological	0.96	(0.91; 1.01)	0.95*	(0.91; 0.99)	0.65***	(0.62; 0.68)
**Hospital catchment region**						
Other	1	(1; 1)	1	(1; 1)	1	(1; 1)
Bentley and Armadale	1.03*	(1.00; 1.06)	0.99	(0.97; 1.02)	0.99	(0.96; 1.02)

* *p* <0.05, ** *p* <0.01, *** *p* <0.001

For peer review only

CT: Computed tomography, ED: emergency department, RR: incidence rate ratio, ALH: All haematopoietic cancer.
RRs were also adjusted for interactions between time in months before and after cancer diagnosis and cancer groups; time in years of cancer diagnosis cohort and cancer groups; and Indigenous status. Please note Indigenous status cannot be reported as per restrictions imposed by the human research ethics approval and can only be used for model adjustment purposes. Month post diagnosis = 1 includes the day diagnosed..

For peer review only

Appendix C. Multivariable negative binomial regression of total use of CT scanning, ED presentation, and hospitalisations in a year after diagnosis with cancer over the study period

	CT scan		Hospitalisations		ED presentations	
	RR	95%CI	RR	95%CI	RR	95%CI
**Cancer group**						
Solid cancer	1	(1; 1)	1	(1; 1)	1	(1; 1)
ALH cancer	1.70***	(1.53; 1.89)	1.43***	(1.31; 1.55)	1.31***	(1.15; 1.49)
**Year of cancer diagnosis**						
2004	1	(1; 1)	1	(1; 1)	1	(1; 1)
2005	1.09***	(1.04; 1.15)	0.98	(0.95; 1.02)	0.97	(0.91; 1.03)
2006	0.97	(0.93; 1.01)	0.92***	(0.89; 0.96)	0.95	(0.90; 1.01)
2007	1.03	(0.99; 1.08)	0.99	(0.96; 1.03)	0.95	(0.89; 1.01)
2008	1.07**	(1.02; 1.12)	1.00	(0.96; 1.03)	0.99	(0.94; 1.05)
2009	0.99	(0.95; 1.04)	1.04*	(1.00; 1.08)	1.02	(0.97; 1.08)
2010	0.99	(0.95; 1.04)	1.07***	(1.03; 1.10)	1.08**	(1.02; 1.14)
2011	1.05*	(1.01; 1.10)	1.10***	(1.06; 1.14)	1.12***	(1.06; 1.19)
2012	1.04	(1.00; 1.09)	1.08***	(1.04; 1.11)	1.16***	(1.10; 1.22)
2013	1.05*	(1.00; 1.10)	1.07***	(1.03; 1.11)	1.14***	(1.08; 1.21)
2014	1.06**	(1.01; 1.11)	1.02	(0.99; 1.06)	1.14***	(1.08; 1.20)
**Interaction term of cancer diagnosis year and cancer type**						
2004 # solid cancer	1	(1; 1)	1	(1; 1)	1	(1; 1)
2004 # ALH	1	(1; 1)	1	(1; 1)	1	(1; 1)
2005 # solid cancer	1	(1; 1)	1	(1; 1)	1	(1; 1)
2005 # ALH	0.84*	(0.72; 0.97)	1.01	(0.90; 1.14)	1.04	(0.87; 1.25)
2006 # solid cancer	1	(1; 1)	1	(1; 1)	1	(1; 1)
2006 # ALH	0.83*	(0.72; 0.96)	0.96	(0.86; 1.09)	0.93	(0.78; 1.12)
2007 # solid cancer	1	(1; 1)	1	(1; 1)	1	(1; 1)
2007 # ALH	0.70***	(0.60; 0.81)	1.01	(0.90; 1.14)	1.06	(0.89; 1.28)
2008 # solid cancer	1	(1; 1)	1	(1; 1)	1	(1; 1)
2008 # ALH	0.65***	(0.56; 0.75)	1.06	(0.94; 1.19)	0.99	(0.82; 1.18)
2009 # solid cancer	1	(1; 1)	1	(1; 1)	1	(1; 1)
2009 # ALH	0.59***	(0.51; 0.68)	0.98	(0.87; 1.10)	1.07	(0.90; 1.28)
2010 # solid cancer	1	(1; 1)	1	(1; 1)	1	(1; 1)
2010 # ALH	0.48***	(0.42; 0.56)	0.94	(0.84; 1.05)	1.09	(0.92; 1.29)
2011 # solid cancer	1	(1; 1)	1	(1; 1)	1	(1; 1)
2011 # ALH	0.51***	(0.44; 0.58)	0.88*	(0.79; 0.98)	0.99	(0.84; 1.17)
2012 # solid cancer	1	(1; 1)	1	(1; 1)	1	(1; 1)
2012 # ALH	0.56***	(0.48; 0.64)	0.97	(0.87; 1.08)	0.92	(0.78; 1.09)
2013 # solid cancer	1	(1; 1)	1	(1; 1)	1	(1; 1)
2013 # ALH	0.55***	(0.48; 0.63)	0.93	(0.83; 1.04)	1.03	(0.87; 1.21)

For peer review only

	CT scan		Hospitalisations		ED presentations	
	RR	95%CI	RR	95%CI	RR	95%CI
2014 # solid cancer	1	(1; 1)	1	(1; 1)	1	(1; 1)
2014 # ALH	0.51***	(0.45; 0.59)	1.04	(0.93; 1.16)	1.03	(0.87; 1.21)
**Sex**						
Male	1	(1; 1)	1	(1; 1)	1	(1; 1)
Female	1.04***	(1.03; 1.06)	1.24***	(1.22; 1.26)	1.03**	(1.01; 1.06)
**Age- group at diagnosis**						
18-45 years	1	(1; 1)	1	(1; 1)	1	(1; 1)
46-65 years	0.98	(0.95; 1.01)	0.94***	(0.91; 0.96)	0.77***	(0.74; 0.80)
66-75 years	0.85***	(0.83; 0.88)	0.75***	(0.73; 0.76)	0.73***	(0.70; 0.76)
>75 years	0.59***	(0.57; 0.61)	0.47***	(0.46; 0.49)	0.74***	(0.71; 0.77)
**Socio-economic status**						
Least disadvantage	1	(1; 1)	1	(1; 1)	1	(1; 1)
Less disadvantage	1.07***	(1.04; 1.10)	1.00	(0.98; 1.03)	1.32***	(1.28; 1.37)
Moderate disadvantage	1.05***	(1.02; 1.08)	1.00	(0.98; 1.03)	1.49***	(1.45; 1.54)
High disadvantage	1.08***	(1.05; 1.11)	1.00	(0.98; 1.02)	1.69***	(1.63; 1.74)
Highest disadvantage	1.07***	(1.04; 1.11)	0.97*	(0.94; 0.99)	1.76***	(1.69; 1.83)
Unknown	1.04	(0.89; 1.22)	0.93	(0.83; 1.06)	1.32**	(1.09; 1.60)
**Accessibility to services**						
Major cities	1	(1; 1)	1	(1; 1)	1	(1; 1)
Inner regional	0.87***	(0.85; 0.90)	0.95***	(0.93; 0.97)	1.23***	(1.19; 1.27)
Outer regional	0.85***	(0.82; 0.88)	0.89***	(0.87; 0.92)	1.59***	(1.53; 1.65)
Remote	0.87***	(0.80; 0.94)	0.83***	(0.78; 0.88)	1.74***	(1.60; 1.88)
Very remote	0.92**	(0.88; 0.97)	0.83***	(0.79; 0.86)	2.33***	(2.20; 2.46)
Unknown	1.03	(0.80; 1.34)	0.77*	(0.62; 0.95)	0.95	(0.68; 1.33)
**Number of comorbidities**						
0	1	(1; 1)	1	(1; 1)	1	(1; 1)
1-2	1.81***	(1.72; 1.91)	3.54***	(3.39; 3.70)	1.18***	(1.10; 1.26)
3-5	2.77***	(2.63; 2.92)	5.96***	(5.71; 6.23)	2.01***	(1.89; 2.15)
6+	4.03***	(3.82; 4.24)	8.29***	(7.94; 8.66)	4.55***	(4.26; 4.86)
**Basis of diagnosis**						
Non-pathological	1	(1; 1)	1	(1; 1)	1	(1; 1)
Pathological	1.29***	(1.22; 1.35)	1.84***	(1.77; 1.92)	0.89***	(0.84; 0.94)
**Hospital catchment region**						
Other	1	(1; 1)	1	(1; 1)	1	(1; 1)
Bentley and Armadale	1.03*	(1.00; 1.06)	1.00	(0.98; 1.02)	0.97	(0.94; 1.00)

* $p < 0.05$, ** $p < 0.01$, *** $p < 0.001$

CT: Computed tomography, ED: emergency department, RR: incidence rate ratio, ALH: All haematopoietic cancer.

RRs were also adjusted for interactions between time in months before and after cancer diagnosis and cancer groups; time in years of cancer diagnosis cohort and cancer groups; and Indigenous status. Please note Indigenous status cannot be reported as per restrictions imposed by the human research ethics approval and can only be used for model adjustment purposes. Month post diagnosis = 1 includes the day diagnosed

For peer review only

The RECORD statement – checklist of items, extended from the STROBE statement, that should be reported in observational studies using routinely collected health data.

	Item No.	STROBE items	Location in manuscript where items are reported	RECORD items	Location in manuscript where items are reported
**Title and abstract**					
	1	(a) Indicate the study's design with a commonly used term in the title or the abstract (b) Provide in the abstract an informative and balanced summary of what was done and what was found		RECORD 1.1: The type of data used should be specified in the title or abstract. When possible, the name of the databases used should be included.<br><br>RECORD 1.2: If applicable, the geographic region and timeframe within which the study took place should be reported in the title or abstract.<br><br>RECORD 1.3: If linkage between databases was conducted for the study, this should be clearly stated in the title or abstract.	Title, abstract (pp. 1, 3-4)
**Introduction**					
Background rationale	2	Explain the scientific background and rationale for the investigation being reported			Throughout background (pp. 5-6)
Objectives	3	State specific objectives, including any prespecified hypotheses			Background, the sentence starting "The present study, therefore, aims …" (p.6)
**Methods**					
Study Design	4	Present key elements of study design early in the paper			Methods, the paragraph starting with "This population-based retrospective

For peer review only

					cohort study …" (p. 6)
Setting	5	Describe the setting, locations, and relevant dates, including periods of recruitment, exposure, follow-up, and data collection			Methods section and subheading "Data source" (p. 6)
Participants	6	*(a) Cohort study* - Give the eligibility criteria, and the sources and methods of selection of participants. Describe methods of follow-up<br>*Case-control study* - Give the eligibility criteria, and the sources and methods of case ascertainment and control selection. Give the rationale for the choice of cases and controls<br>*Cross-sectional study* - Give the eligibility criteria, and the sources and methods of selection of participants<br><br>*(b) Cohort study* - For matched studies, give matching criteria and number of exposed and unexposed<br>*Case-control study* - For matched studies, give matching criteria and the number of controls per case		RECORD 6.1: The methods of study population selection (such as codes or algorithms used to identify subjects) should be listed in detail. If this is not possible, an explanation should be provided.<br><br>RECORD 6.2: Any validation studies of the codes or algorithms used to select the population should be referenced. If validation was conducted for this study and not published elsewhere, detailed methods and results should be provided.<br><br>RECORD 6.3: If the study involved linkage of databases, consider use of a flow diagram or other graphical display to demonstrate the data linkage process, including the number of individuals with linked data at each stage.	Methods subheading "Study population" (p.7)<br><br>No validation study to report<br><br>Described in the "Method" section. (pp.6-7)
Variables	7	Clearly define all outcomes, exposures, predictors, potential confounders, and effect modifiers. Give diagnostic criteria, if applicable.		RECORD 7.1: A complete list of codes and algorithms used to classify exposures, outcomes, confounders, and effect modifiers should be provided. If these cannot be reported, an explanation should be provided.	Methods subheadings "Use of CT", "Hospitalisations and ED Presentations" and "Covariates" (p. 8).

For peer review only

Data sources/ measurement	8	For each variable of interest, give sources of data and details of methods of assessment (measurement). Describe comparability of assessment methods if there is more than one group			Methods subheadings "Use of CT", "Hospitalisations and ED Presentations", and "Covariates" (p. 8).
Bias	9	Describe any efforts to address potential sources of bias			Methods subheadings "Covariates" and "Analysis" (pp. 9)
Study size	10	Explain how the study size was arrived at			Study population (page 7)
Quantitative variables	11	Explain how quantitative variables were handled in the analyses. If applicable, describe which groupings were chosen, and why			Methods subheading "Use of CT", "Hospitalisations and ED Presentations", "Covariates" (p. 8) and Table 1.
Statistical methods	12	(a) Describe all statistical methods, including those used to control for confounding<br>(b) Describe any methods used to examine subgroups and interactions<br>(c) Explain how missing data were addressed<br>(d) *Cohort study* - If applicable, explain how loss to follow-up was addressed<br>*Case-control study* - If applicable, explain how matching of cases and controls was addressed			Methods subheading "Analysis" (p.9)

For peer review only

		Cross-sectional study - If applicable, describe analytical methods taking account of sampling strategy<br>(e) Describe any sensitivity analyses			
Data access and cleaning methods		..		RECORD 12.1: Authors should describe the extent to which the investigators had access to the database population used to create the study population.<br><br>RECORD 12.2: Authors should provide information on the data cleaning methods used in the study.	
Linkage		..		RECORD 12.3: State whether the study included person-level, institutional-level, or other data linkage across two or more databases. The methods of linkage and methods of linkage quality evaluation should be provided.	Linkage described in "Methods" section (p.6)
**Results**					
Participants	13	(a) Report the numbers of individuals at each stage of the study (*e.g.*, numbers potentially eligible, examined for eligibility, confirmed eligible, included in the study, completing follow-up, and analysed)<br>(b) Give reasons for non-participation at each stage.<br>(c) Consider use of a flow diagram		RECORD 13.1: Describe in detail the selection of the persons included in the study (*i.e.,* study population selection) including filtering based on data quality, data availability and linkage. The selection of included persons can be described in the text and/or by means of the study flow diagram.	Results (p.9)
Descriptive data	14	(a) Give characteristics of study participants (*e.g.*, demographic, clinical, social) and information			Participants described in results section (p.9) and Table 1.

		on exposures and potential confounders<br>(b) Indicate the number of participants with missing data for each variable of interest<br>(c) *Cohort study* - summarise follow-up time (*e.g.*, average and total amount)			
Outcome data	15	*Cohort study* - Report numbers of outcome events or summary measures over time<br>*Case-control study* - Report numbers in each exposure category, or summary measures of exposure<br>*Cross-sectional study* - Report numbers of outcome events or summary measures			Tables 1.
Main results	16	(a) Give unadjusted estimates and, if applicable, confounder-adjusted estimates and their precision (e.g., 95% confidence interval). Make clear which confounders were adjusted for and why they were included<br>(b) Report category boundaries when continuous variables were categorized<br>(c) If relevant, consider translating estimates of relative risk into absolute risk for a meaningful time period			Figures 1-2, Table 2 and Appendices A-C.
Other analyses	17	Report other analyses done—e.g., analyses of subgroups and interactions, and sensitivity analyses			Appendix B-C
**Discussion**					

For peer review only

Key results	18	Summarise key results with reference to study objectives			Throughout discussion and the main conclusions are in section "Conclusion" (pp.15)
Limitations	19	Discuss limitations of the study, taking into account sources of potential bias or imprecision. Discuss both direction and magnitude of any potential bias		RECORD 19.1: Discuss the implications of using data that were not created or collected to answer the specific research question(s). Include discussion of misclassification bias, unmeasured confounding, missing data, and changing eligibility over time, as they pertain to the study being reported.	Discussed in a sub-section "Strengths and limitations" (p.14)
Interpretation	20	Give a cautious overall interpretation of results considering objectives, limitations, multiplicity of analyses, results from similar studies, and other relevant evidence			Throughout discussion
Generalisability	21	Discuss the generalisability (external validity) of the study results		all, or who presented to any public hospital emergency department (ED), or who had a CT scan undertaken in WA by a public or private provider between 1 January 2003 and 31 December 2015	In Method, we state that contains Western Australian data for instance "…Western Australians aged 18+ who had a separation (discharge) from any WA hospital for any non-pregnancy-related condition …" (p. 6), and throughout

For peer review only

					Discussion (p. 12-13) with more specific statements in limitation sub-section (pp. 14).
**Other Information**					
Funding	22	Give the source of funding and the role of the funders for the present study and, if applicable, for the original study on which the present article is based			Funding heading (p.16)
Accessibility of protocol, raw data, and programming code		..		RECORD 22.1: Authors should provide information on how to access any supplemental information such as the study protocol, raw data, or programming code.	The data that support the findings of this study are available from the relevant data custodians of the study datasets. Restrictions by the data custodians mean that the data are not publicly available or able to be provided by the authors. Researchers wishing to access the datasets used in this study should refer to the Western Australian Data Linkage Unit and the Australian Institute of Health

For peer review only

					and Welfare. (p. 16).

*Reference: Benchimol EI, Smeeth L, Guttmann A, Harron K, Moher D, Petersen I, Sørensen HT, von Elm E, Langan SM, the RECORD Working Committee. The REporting of studies Conducted using Observational Routinely-collected health Data (RECORD) Statement. *PLoS Medicine* 2015; in press.

*Checklist is protected under Creative Commons Attribution (CC BY) license.

For peer review only

BMJ Open

The use of computed tomography, ED presentation and hospitalisations 12-months before and after a diagnosis of cancer in Western Australia: A population-based retrospective cohort study

Journal:	*BMJ Open*
Manuscript ID	bmjopen-2022-071052.R1
Article Type:	Original research
Date Submitted by the Author:	23-Jun-2023
Complete List of Authors:	Ha, Ninh; Curtin University Bentley Campus,<br>Kamarova, Sviatlana; Curtin University, School of Population Health<br>Youens, David; Curtin University, School of Population Health<br>Ho, Chau; Curtin University Bentley Campus,<br>Bulsara, Max; University of Notre Dame, Biostatistics<br>Doust, Jenny; The University of Queensland Faculty of Medicine and Biomedical Sciences<br>Mcrobbie, Donald; University of Adelaide , Medical Physics<br>O'Leary, Peter ; Curtin University Bentley Campus, School of Public Health; The University of Western Australia, Obstetrics and Gynaecology Medical School, Faculty of Health and Medical Sciences<br>Wright, Cameron; University of Tasmania, ; School of Medicine<br>Trevithick, Richard ; 11. Western Australian Cancer Registry, Clinical Excellence Division, Department of Health<br>Moorin, Rachael; Curtin University, ; University of Western Australia,
**Primary Subject Heading**:	Health services research
Secondary Subject Heading:	Health policy, Radiology and imaging, Public health
Keywords:	Computed tomography < RADIOLOGY & IMAGING, Health policy < HEALTH SERVICES ADMINISTRATION & MANAGEMENT, Quality in health care < HEALTH SERVICES ADMINISTRATION & MANAGEMENT, Public health < INFECTIOUS DISEASES, Epidemiology < ONCOLOGY

SCHOLARONE™
Manuscripts

BMJ

I, the Submitting Author has the right to grant and does grant on behalf of all authors of the Work (as defined in the below author licence), an exclusive licence and/or a non-exclusive licence for contributions from authors who are: i) UK Crown employees; ii) where BMJ has agreed a CC-BY licence shall apply, and/or iii) in accordance with the terms applicable for US Federal Government officers or employees acting as part of their official duties; on a worldwide, perpetual, irrevocable, royalty-free basis to BMJ Publishing Group Ltd ("BMJ") its licensees and where the relevant Journal is co-owned by BMJ to the co-owners of the Journal, to publish the Work in this journal and any other BMJ products and to exploit all rights, as set out in our licence.

The Submitting Author accepts and understands that any supply made under these terms is made by BMJ to the Submitting Author unless you are acting as an employee on behalf of your employer or a postgraduate student of an affiliated institution which is paying any applicable article publishing charge ("APC") for Open Access articles. Where the Submitting Author wishes to make the Work available on an Open Access basis (and intends to pay the relevant APC), the terms of reuse of such Open Access shall be governed by a Creative Commons licence – details of these licences and which Creative Commons licence will apply to this Work are set out in our licence referred to above.

Other than as permitted in any relevant BMJ Author's Self Archiving Policies, I confirm this Work has not been accepted for publication elsewhere, is not being considered for publication elsewhere and does not duplicate material already published. I confirm all authors consent to publication of this Work and authorise the granting of this licence.

For peer review only

The use of computed tomography, ED presentation and hospitalisations 12-months before and after a diagnosis of cancer in Western Australia: A population-based retrospective cohort study

Author information

Ninh T. Ha[1], PhD, thi.ha@curtin.edu.au

Sviatlana Kamarova[1, 2, 3], PhD, sviatlana.kamarova@sydney.edu.au

David Youens[1,5], david.youens@curtin.edu.au

Chau Ho[1,] chau.ho@curtin.edu.au

Max Bulsara [4,5], PhD, max.bulsara@nd.edu.au

Jenny Doust[6], BMBS, PhD, j.doust@uq.edu.au

Donald McRobbie[7], PhD, donald.mcrobbie@adelaide.edu.au

Peter O'Leary [1,8,9], PhD, peter.oleary@health.wa.gov.au

Cameron Wright[1,10,11,12], MD, cameron.wright@curtin.edu.au

Richard Trevithick[13], MD, richard.trevithick@health.wa.gov.au

Rachael Moorin,[1,5] PhD, R.Moorin@curtin.edu.au

Affiliations

1. Health Economics and Data Analytics, Curtin School of Population Health, Faculty of Health Sciences, Curtin University, Western Australia;
2. Sydney School of Health Sciences, University of Sydney, New South Wales;
3. Nepean Blue Mountains Local Health District, New South Wales Health, New South Wales, Australia;
4. Institute for Health Research, University of Notre Dame, Western Australia;

For peer review only

5. Centre for Health Services Research, School of Population and Global Health, The University of Western Australia;

6. Australian Women and Girls' Health Research (AWaGHR) Centre, Faculty of Medicine, University of Queensland;

7. School of Physical Sciences, University of Adelaide;

8. Obstetrics and Gynaecology Medical School, Faculty of Health and Medical Sciences, The University of Western Australia, Perth, Western Australia, Australia;

9. PathWest Laboratory Medicine, QE2 Medical Centre, Nedlands, Western Australia, Australia;

10. Fiona Stanley Hospital, 11 Robin Warren Dr, Murdoch, Western Australia, Australia;

11. Division of Internal Medicine, Medical School, Faculty of Health and Medical Sciences, The University of Western Australia;

12. School of Medicine, College of Health and Medicine, University of Tasmania, Hobart, Tasmania;

13. Western Australian Cancer Registry, Clinical Excellence Division, Department of Health, East Perth, Western Australia.

***Corresponding author:**

Ninh Thi Ha

Health Economics & Data Analytics, Curtin School of Population Health, Faculty of Health Sciences, Curtin University

GPO Box U1987, Perth, Western Australia, 6845

Email: Thi.Ha@curtin.edu.au; ninh.ha05@gmail.com

For peer review only

Phone: +61 08 9266 5134

Abstract (300 max)

Objective: To examine the use of CT, ED-presentation, and hospitalisation and in 12-month before and after a diagnosis of cancer.

Design: Population-based retrospective cohort study.

Setting: West Australian linked administrative records at individual level.

Participants: 104,009 adults newly diagnosed with cancer in 2004-2014

Main outcome measures: CT use, ED presentations, hospitalisations.

Results: As compared to the rates in the 12th month before diagnosis, the rate of CT scans started to increase from two months before diagnosis with an increase in both ED presentations and hospitalisation from one month before the diagnosis. These rates peaked in the month of diagnosis for CT scans (477 [95%CI 471-482] per 1000 patients), and for hospitalisations (910 [95%CI 902-919] per 1000 patients), and the month prior to diagnosis for ED (181 [95%CI 178-184] per 1000 patients) then rapidly reduced after diagnosis but remained high for the next 12 months. While the patterns of the health services used were similar between 2004 and 2014, the rate of the health services used during after diagnosis was higher in 2014 vs. 2004 except for CT use in patients with lymphohematopoietic cancer with a significant reduction.

Conclusion: Our results showed an increase in demand for health services from two months before diagnosis of cancer. Increasing use of health services during and post cancer diagnosis may warrant further investigation to identify factors driving this change.

Keywords: computed tomography, ED presentations, hospitalisation, cancer, health services

For peer review only

Strengths and limitations of the study:

- The present study examines (i) the patterns of hospital and ED services in conjunction with (ii) both pre and post diagnostic periods of CT use, which has not been evaluated to date in adult cancer patients.
- The cohort makes use of whole of population data which removes selection bias and minimises loss to follow up, while allowing for grounded and representative conclusions for the target population based on higher statistical precision.
- The present data does not include some important clinical data such as indications for CT scanning and stage of cancer at diagnosis. Some CT scans from secondary hospitals may be missing.

For peer review only

Background

Computed tomography (CT) is useful for screening for cancer; diagnosis of the presence of a tumour and staging of cancer; guiding where to perform a biopsy procedure, where to apply local treatment (e.g. radiofrequency ablation, implantation of radioactive seeds or cryotherapy); planning surgery, directing treatment strategies and assessing treatment response (1-4). Examples of the utility of CT include the National CT colonography trial, where CT colonography provided similar accuracy in the rate of identifying both large colorectal polyps and colorectal tumours in patients with an average risk of colorectal cancer as compared to the gold standard colonoscopy (1). Similarly in the National Lung Screening trial, compared to chest X-ray, screening by CT reduced the risk of mortality from lung cancer in heavy smokers by 20% (2). However, compared to standard X-ray (and non-ionising radiation modalities such as ultrasound and magnetic resonance imaging), CT scanning imparts a relatively high radiation exposure that has raised concerns about the risk of cancer, particularly in children, young adults and women (5-8). In addition to the radiation burden increased use of CT scanning has also been associated with increases in incidental findings and over-diagnosis (9, 10). There is therefore a trade-off between the benefits and risk of CT scanning which has led to calls to evaluate its use to ensure maintenance of high value care.

CT scanning has been steadily increasingly in most developed countries, particularly in cancer care (11-16). Studies in the UK (17) and Denmark (18) reported a significant increase in CT scanning from three months before a cancer diagnosis. A study by Purdie *et al* in New South Wales, Australia observed a similar pattern (19). However, this study included only a small number of participants (n=894) and was limited to the period before a cancer diagnosis. While patterns of visits/consultation by GPs or specialists before and after a cancer diagnosis have been examined previously (20, 21), patterns of ED presentations and hospitalisations in Australia remained unclear. Thus, in the present study, we examined changes in the use of CT,

For peer review only

ED presentations and hospitalisations in the 12-months before and after a diagnosis of cancer for adults diagnosed with cancer between 2004 and 2014 in Western Australia (WA) using linked administrative data.

Methods

Study design

This population-based retrospective cohort study follows the REporting of studies Conducted using Observational Routinely-collected health Data (RECORD) statement (22). Approval was granted by the Western Australia (WA) Department of Health Human Research Ethics Committee (HREC) (2011/97) and the Australian Institute of Health and Welfare (AIHW) HREC (EO2018/4/485), under a waiver of consent.

Data sources and linkage

This study is part of a larger project using person-level linked administrative data pertaining to a cohort comprising all Western Australians aged 18+ who had a separation (discharge) from any WA hospital for any non-pregnancy-related condition, or who presented for any reason to any public hospital emergency department (ED), or who had a CT scan undertaken in WA by a public or private provider between 1 January 2003 and 31 December 2015.

Using State-based data from the WA Data Linkage System (23) and Medicare Benefits Schedule (MBS) data provided by the AIHW, cross-jurisdictional (i.e. State – Commonwealth) privacy-preserving record linkage was undertaken by the Curtin University Centre for Data Linkage (24). The following person-level linked data were available:

1) WA Cancer registry (WACR) data containing all incident cancers in the cohort (irrespective of age at diagnosis) from 1972 onwards, including diagnosis date, basis of diagnosis, site code, morphology code, behaviour type, cancer type code using the International Classification of

For peer review only

Diseases, 10th revision, Australian Modification (25) and demographics (e.g., age at diagnosis, sex, and postcode).

2) WA Hospital morbidity data collection (HMDC) records from 2003-2015 for all discharges from any WA hospital (public or private) for all conditions excluding pregnancy. It included dates and mode of admission and discharge, hospital type (tertiary or secondary), diagnosis (principal and co-diagnoses) and procedure codes.

3) WA Emergency Department Data Collection (EDDC) records 2003-2015 for all WA public hospital ED presentations. It included presentation date and time, presentation type (emergency or planned), triage code, diagnosis code and major diagnostic group.

4) WA Picture Archiving and Communication System (PACS) records for all CT scans (excluding position emission tomography (PET)/CT) 2003-2015 undertaken in all WA public tertiary hospitals and selected public secondary hospitals (limited to hospitals who do not contract out radiology services) as an out-patient, ED patient or admitted patient. It included the scan date, the type of CT examination (e.g., non-contrast Head CT etc.), and referral source.

5) MBS data capturing all CT scans subsidised by the Federal Australian Government (i.e., those performed outside of hospital or in hospital for private patients), including the date and scan type, between 2003 – 2015.

6) WA Death Registrations records from 2003-2015 including the age and date of death.

Study population

The study population was limited to patients aged 18+ years who were diagnosed with a first invasive cancer between 1 January 2004 and 31 December 2014. We excluded individuals who had a diagnosis of cancer after death or those who died within 30 days of a diagnosis of cancer in the present analysis.

For peer review only

Patient and Public Involvement

Patients were not directly involved in the study design or conduct. Our consumer representative (Mr John Stubbs) was involved in the design of the grant used to fund this research and is an ongoing member of the research team providing ongoing input to analysis of the data, interpretation of the results and development of publications. The Western Australian Data Linkage Branch and the data custodians of the WA Emergency Department Data Collection and the Picture Archiving Communications System data provided data for this project.

Outcome measurement

The primary outcome was number of CT scans performed within 12 months prior and 12 months post cancer diagnosis. Multiple CT scans within the same day of the same anatomical area were counted as a single CT event (26). Similarly, we measured the number of ED presentations and hospitalisations within 12-months prior to and post cancer diagnosis. Numbers of CT scans, hospitalisations and ED presentations were captured monthly and annually for all cancer diagnosis in the study period from 2004 to 2014.

Covariates

Cancer types were grouped as either solid or all lymphohaematopoietic (ALH) to examine the effect of this broad classification on outcomes of interest. Demographic characteristics included sex, age group at cancer diagnosis, and Indigenous status (captured using Data Linkage WA's Derived Indigenous Flag) (27). Accessibility to services was measured using the accessibility/remoteness of index of Australia (ARIA) (28) based on postcode at the diagnosis date. Socio-economic status was classified using the socio-economic indexes for areas – index of relative socio-economic disadvantage (SEIFA-IRSD) based on postcode, for the census closest to diagnosis date categorised as quintiles (29). Secondary hospitals in the

For peer review only

suburbs of Bentley and Armadale did not contribute data on CT scans to PACS, as they contract out their radiology services to private providers, although data for CT scans from out-of-hospital and those attending tertiary hospital who live in these suburbs were provided. Hospital catchment area was stratified as 'Bentley and Armadale' and 'others' to adjust for any potential bias in the regression model. Comorbidity was determined using the Multipurpose Australian Comorbidity Scoring System (MACSS) (30)) capturing the number of 17 MACSS conditions recorded on any hospitalisation across the five years prior to cancer diagnosis. Referral sources of CT scans were grouped as ED physician, inpatient and out-of-hospital. Cancer diagnosis was also classified as either pathology or non-pathology-based diagnosis.

Statistical Analysis

Descriptive analyses were used to summarise the study population characteristics at the beginning of the study (2004), the end of the study (2014) and across the whole study period (2004-2014). After assessing the data for suitability (over-dispersion) and model fit (based on Akaike and Bayesian information criterion) multivariable negative binomial regression was performed to calculate the monthly rates of CT scanning, ED presentations, and hospitalisation 12 months prior to and 12 months post cancer diagnosis adjusted for all observed socio-demographic and clinical characteristics and year of diagnosis. Similarly, monthly rates of CT scanning, ED presentations and hospitalisation 12 months prior to and post cancer diagnosis were conducted for sub-cancer types. Multivariable negative binomial regression was also used to examine trends in total CT scanning use, ED presentations and hospitalisations in the year prior to and after diagnosis over the study period (2004 to 2014) adjusted for all observed characteristics and year of diagnosis. Analyses used Stata SE version 15 (31).

Results

Characteristics of the study population

For peer review only

A total of 104,009 individuals had a first diagnosis of cancer from 2004 to 2014 (Table 1). In Table 1, the majority of the population were male (56.2%), aged 46-65 years (40.5%), classified as living in an area of least socio-economic disadvantage (31.2%), lived in major cities (68.8%), and had at least one comorbidity (95.3%). In terms of cancer diagnosis, the most common type was solid cancer (91.2%) in which cancer of the male genital organs, digestive organs, melanoma and breast shared high proportions of 18.6%, 17.5%, 15.7% and 13.7% correspondingly. Up to 97% of cancer diagnoses were based on pathological methods. Both demographic and clinical characteristics were similar between the beginning (2004) and end of the study (2014).

For peer review only

1 **Table 1. Characteristics of the Study Population**

Characteristics		Year of cancer diagnosis 2004 n=8,081		2014 n = 10,884		2004-2014 (overall) n=104,009	
		n	%	*n*	%	*n*	%
**Sex**	Male	4,468	55.29%	5,977	54.92%	58,411	56.16%
	Female	3,613	44.71%	4,907	45.08%	45,598	43.84%
**Age group at diagnosis**	18-45 years	909	11.25%	1,106	10.16%	10,827	10.41%
	46-65 years	3,243	40.13%	4,283	39.35%	42,104	40.48%
	66-75 years	2,008	24.85%	2,980	27.38%	26,142	25.13%
	>75 years	1,921	23.77%	2,515	23.11%	24,936	23.97%
**Socio-economic status**†	Least disadvantage	2,419	29.93%	2,939	27.00%	32,464	31.21%
	Less disadvantage	1,224	15.15%	1,673	15.37%	15,295	14.71%
	Moderate disadvantage	1,939	23.99%	1,972	18.12%	21,954	21.11%
	High disadvantage	1,664	20.59%	2,941	27.02%	23,913	22.99%
	Highest disadvantage	764	9.45%	1,351	12.41%	9,893	9.51%
	Unknown	71	0.88%	8	0.07%	490	0.47%
**Accessibility to services**‡	Major cities	5,286	65.41%	7,902	72.60%	71,574	68.82%
	Inner regional	1,684	20.84%	1473	13.55%	17,646	16.97%
	Outer regional	751	9.29%	964	8.86%	9,623	9.25%
	Remote	98	1.21%	244	2.24%	1,454	1.40%
	Very remote	250	3.09%	#	#	3,535	3.40%
	Unknown#	12	0.15%	#	#	177	0.17%
**Bases of diagnosis**	Non-pathological	249	3.12%	360	3.35%	3,413	3.32%
	Pathological	7,738	96.88%	10,398	96.65%	99,536	96.68%
**Hospital catchment area**	Other	6,742	83.43%	9,142	83.99%	87,384	84.02%
	Bentley and Armadale	1,339	16.57%	1,742	16.01%	16,625	15.98%
**Number of comorbidities**§	None	746	9.23%	383	3.52%	4,854	4.67%
	1-2	2,502	30.96%	1,856	17.05%	22,136	21.28%

For peer review only

Characteristics		Year of cancer diagnosis: 2004 n=8,081		Year of cancer diagnosis: 2014 n = 10,884		2004-2014 (overall) n=104,009	
		n	%	*n*	%	*n*	%
	3-5	2,773	34.32%	3,830	35.19%	37,740	36.29%
	6+	2,060	25.49%	4,815	44.24%	39,279	37.77%
**Solid cancer**	Cancer of the male genital organs	1,436	17.77%	1,830	16.81%	19,353	18.61%
	Cancer of the digestive organs	1,438	17.79%	1,753	16.11%	18,222	17.52%
	Melanoma	1,280	15.84%	1,891	17.37%	16,292	15.66%
	Breast cancer	1,087	13.45%	1,630	14.98%	14,262	13.71%
	Cancer of the respiratory organs	679	8.40%	875	8.04%	8,381	8.06%
	Cancer of the urinary tract	322	3.98%	511	4.69%	4,541	4.37%
	Cancer of the female genital organs	348	4.31%	438	4.02%	4,130	3.97%
	Mouth and pharynx cancer	229	2.83%	322	2.96%	3,049	2.93%
	Thyroid cancer	148	1.83%	238	2.19%	2,147	2.06%
	Brain cancer	111	1.37%	158	1.45%	1,523	1.46%
	Cancer of the soft tissues	114	1.41%	153	1.41%	1,459	1.40%
	Cancer of unspecified sites	121	1.50%	112	1.03%	1,318	1.27%
	Bone cancer	9	0.11%	12	0.11%	157	0.15%
	All Solid	7,322	90.61%	9,923	91.17%	94,834	91.18%
**All haematopoietic cancer**§	Leukaemias and myelodysplasias	327	4.05%	330	3.03%	3,676	3.53%
	Other lymphomas	203	2.51%	292	2.68%	2,742	2.64%
	Other lymphoid cancers	187	2.31%	289	2.66%	2,280	2.19%
	Hodgkin's lymphoma	42	0.52%	50	0.46%	477	0.46%
	All lymphoid and haematopoietic cancer	759	9.39%	961	8.83%	9,175	8.82%

†Socio-economic status measured by SEIFA-IRSD: Socio-Economic Indexes for Areas, Index of Relative Socio-Economic Disadvantage.
‡Accessibility to services was measured using ARIA: Accessibility and Remoteness Index of Australia.
§ Comorbidity: Number of comorbid conditions reported in hospitalisation data in the 5 years prior to the date of cancer diagnosis
¶ED presentations: Emergency department presentations

For peer review only

[#]Where small cells are suppressed, random adjustments have been made to other cells within different levels of the same variable to prevent calculation of suppressed cell counts

Note. Indigenous status cannot be reported as per restrictions imposed by the human research ethics approval and can only be used for model adjustment purposes. 12 months after the diagnosis is inclusive of the date of the diagnosis.

For peer review only

Use of CT scanning 12-months before and after cancer diagnosis and trends over the study period

The adjusted rate of CT scanning 12 months before and after cancer diagnosis is presented in table 2. At 12 months prior to cancer diagnosis, the adjusted rate of CT scanning was 14 CT scans per 1000 patients (95%CI 13-15) and increased to 35 CT scans per 1000 patients (95%CI 33-36) at three months before diagnosis. The rate steeply increased to 294 CT scans per 1000 patients (95%CI 290-298) in the month before diagnosis and reached the highest rate of 477 CT scans per 1000 patient (95%CI 471-482) in the month of diagnosis. After cancer diagnosis, the rate of CT scanning dramatically reduced but remained elevated compared with the rate pre diagnosis (Further details of regression model output is presented in appendix A). Similar patterns were observed across sub-cancer types as shown in Appendix B.

For peer review only

Table 2. Adjusted rate of CT scanning, ED presentations and Hospitalisations in 12 months before and after cancer diagnosis

Month before and after cancer diagnosis	CT scanning		Hospitalisations		ED presentations	
	Rate/1000 patients	95%CI	Rate/1000 patients	95%CI	Rate/1000 patients	95%CI
-12	14	(13; 15)	48	(46; 50)	26	(25; 27)
-11	14	(13; 15)	51	(48; 53)	27	(25; 28)
-10	14	(13; 15)	51	(49; 53)	27	(26; 28)
-9	15	(14; 15)	52	(49; 54)	28	(26; 29)
-8	15	(15; 16)	52	(50; 55)	28	(27; 29)
-7	16	(15; 17)	56	(53; 58)	29	(28; 30)
-6	18	(17; 19)	56	(53; 58)	30	(29; 31)
-5	20	(19; 21)	58	(56; 61)	32	(31; 33)
-4	24	(23; 25)	64	(62; 67)	37	(35; 38)
-3	35	(33; 36)	73	(70; 75)	43	(42; 45)
-2	69	(67; 71)	94	(91; 96)	59	(58; 61)
-1	294	(290; 298)	371	(367; 375)	181	(178; 184)
1	477	(471; 482)	910	(902; 919)	125	(123; 128)
2	194	(190; 197)	624	(617; 631)	104	(102; 106)
3	150	(146; 153)	636	(629; 644)	94	(91; 96)
4	133	(130; 136)	587	(580; 595)	84	(82; 87)
5	120	(117; 123)	502	(496; 509)	75	(73; 77)
6	119	(116; 122)	431	(425; 438)	70	(68; 72)
7	119	(116; 122)	369	(363; 375)	63	(61; 65)
8	111	(108; 114)	312	(306; 318)	61	(59; 63)
9	100	(97; 103)	259	(254; 264)	57	(55; 58)
10	95	(92; 98)	236	(231; 241)	56	(54; 58)
11	93	(90; 96)	223	(218; 228)	53	(51; 55)
12	95	(92; 97)	214	(209; 219)	52	(50; 54)

CT: Computed tomography, ED: emergency department, CI: confidence interval.

Notes: full model with adjusted covariates is presented in appendix A.

For peer review only

Figure 1 demonstrates the pattern of CT scanning 12 months before and after cancer diagnosis by two main cancer groups for two time points 2004 and 2014 for simplicity. While overall the rate of CT scanning is higher in patients diagnosed with ALH cancer than solid cancer, the patterns of CT use observed across the period 12 months pre and post cancer diagnosis were similar for both groups. For both years a peak in CT use occurred in the month of diagnosis and the peak was more pronounced for ALH cancers than solid cancer.

Further details in Appendix A shows that patients with a higher number of comorbidities were more likely to have CT scanning with an RR 4.94 (95%CI 4.62-5.27) for those with at least six comorbidities vs those without any comorbidities. The rate of CT scanning stratified by gender, age, socio-economic, accessibility to services, hospital catchment regions, cancer diagnosis methods was slightly different as compared to the reference group in each category.

Figure 2- panel A presents trends in total CT scans used in 12 months before and after diagnosis by cancer groups over the study period from 2004 to 2014. For 12 months before diagnosis, the rate of CT used in ALH cancer was consistently higher than those of solid cancers diagnosed over the entire study period. However, the rate of scans 12 months after diagnosis of ALH cancer significantly reduced between 2004 and 2009, following which the difference in the magnitude of the rate of CT scanning across the two cancer types disappeared (Further details of regression model output is presented in Appendix C & D).

ED presentations and hospitalisation 12-month before and after cancer diagnosis and their trends over the study period

Similarly, to the pattern observed for CT scanning, as shown in Table 2 when adjusted for year of diagnosis, the rate of hospitalisation was highest in the month of diagnosis at 910 per 1,000 patients (95% CI 902-919) whereas for ED presentations the peak was in the month prior to diagnosis at 181 per 1,000 patients (95% CI 178-184). While the rate dropped in the months

For peer review only

after diagnosis for both ED presentations and hospitalisations, it remained consistently higher than the rate before diagnosis. (Further details of regression model output is presented in appendix A).

As shown in Figure 1- panel B and C, no significant difference was observed in the rate of ED presentations or hospitalisation 12 months before cancer diagnosis in 2004 compared with 2014. However, significantly increased rates of both ED presentations and hospitalisations were observed in 2014 compared with those in 2004 for the month of diagnosis and thereafter. Similar patterns were observed for both cancer groups.

Further details in appendix A show that patients with cancer diagnosis based on pathological methods were 34% less likely to have an ED presentation compared to those with cancer diagnosis based on non-pathological methods (RR 0.66 [95%CI 0.63-0.69]) whereas no significant difference was reported for hospitalisation rate. Increasing number of comorbidities was associated with substantially higher rates of both ED presentations and hospitalisations in which the RR between those with none vs those with at least six comorbidities was highest at 5.54 (95%CI 5.15-5.96) for ED presentations and 11.5 (95%CI 10.5-12.5) for hospitalisations. The rate of ED presentations also significantly increased with higher social disadvantage and with living in regional or remote areas. Small or no differences in hospitalisation and ED presentation rates were recorded in subgroups stratified by sex, age, cancer groups and hospital catchment region.

In Figure 2-panel B and C, over the entire study period the rate of both hospitalisation and ED presentations for those with ALH cancer were consistently higher than those with solid cancer both before and after diagnosis with little change in rate across the year of diagnosis (Further details of regression model output is presented in Appendix C & D).

Discussion

For peer review only

Our study found that the rate of CT scanning started to increase from two months before diagnosis while ED presentations and hospitalisation started to increase from one month before diagnosis. These rates peaked in the month of diagnosis then rapidly reduced within the first month after diagnosis but remained high for the next 12 months. Our results were consistent with those reported in previous studies that showed an initial increase in the use of CT scanning or 'diagnostic investigation' (e.g., x-ray, ultrasound, CT scan, MRI scan, angiography, endoscopies and biopsies) two months prior to cancer diagnosis (19, 21). Other studies have investigated use of CT scanning in specific cancer types with a UK study finding that rates of CT use started to increase five months before a diagnosis of bladder and kidney cancer (17), and a Danish study finding an increase CT use associated with abdominal cancers diagnosed from 2014 to 2018 (18). In comparison our study evaluated changes in use of CT scanning associated with cancer diagnosis more globally and compared across a longer time period (2004-2014). The 10 year time frame means that our study period would have included more substantial changes in health care policy, cancer treatments and imaging technology that may have contributed to changes in CT (32). While it was out of the scope of this study to evaluate the incremental impact of these changes, it is worth noting the extensive changes that have occurred over this time period that have cumulatively led to the changes in CT scanning observed.

In our study, although the rate of CT scanning was low at 4 months or more prior to the cancer diagnosis date, 30.4% of cancer patients had the first CT scan performed at 4 months or more before the diagnosis date. Evidence shows that out-of-hospital referrals are more likely to be the source of early diagnosis using CTs (33, 34), whereas those referred as in-patients and emergency patients would be more likely to have more advanced cancers. Due to limited access to general practitioners and specialists or difficulty in funding out-of-hospital co-payments, some Australian patients access primary care via EDs seeking diagnostic care (35). Moreover,

For peer review only

the likelihood of early CT use potentially could be driven by defensive diagnosis aimed to exclude later staging or advanced disease (whether it is the primary site or of metastatic nature) (36). This aligns with earlier evidence from the UK of insufficient CT referrals for brain tumours, where existing guidelines do not adequately identify clinical symptoms useful for the basis of CT imaging (37).

Our study is consistent with a study in NSW, Australia that reported a similar pattern on the increase in rate of ED presentation and hospitalisation before diagnosis that dramatically reduced after diagnosis, although the NSW study was limited to admissions from ED only (20). The observed increase in the use of CT scanning, ED presentations and hospitalisations in our study suggests a 'diagnostic time window' of two months whereas the increasing rate of general practice consultations have previously been found to start earlier, from three to twelve months before the diagnosis (21, 38, 39). This time disparity between patients who ultimately are diagnosed with cancer initially presenting to their doctors and CT use could reflect issues with access to CT or indicate that CT is predominantly used for staging and guiding treatment direction post initial diagnosis. It also reflects that there may be a more complex diagnostic process with many steps prior to CT use. Since our study was limited to patients with a diagnosis of cancer, we cannot comment on the use of CT for those who were ultimately found not to have cancer but where CT used to rule this out.

Over the whole 10-year study period, patients with an ALH cancer were more likely to have CT scans than those with a solid cancer. This may be explained by the principal role of CT scans in staging patients with ALH cancer, particularly lymphomas (40-42) while solid cancers can be diagnosed by different imaging (e.g. MRI, mammography, ultrasound, etc.), blood tests for biomarkers and biopsies that varies by cancer specific types. However, this hypothesis is not supported by our comparison of changes in CT scanning in 2004 compared with 2014, where we found that the rate of CT scanning reduced for patients with ALH cancer and

For peer review only

increased for those with solid cancers. The reduction of CT scanning for patients with ALH cancer may be explained by the uptake of PET/CT scanning – a powerful tool for oncology which became available in Western Australia in early 2002 (43). However, CT/PET scans are not captured by our data as it is limited to stand alone CT scanning, therefore we cannot determine to what extent substitution between CT and PET/CT has taken place.

Also, we observed a higher rate of ED presentation in cancer patients with higher disadvantage socioeconomic status and higher number of comorbidities that was in line with results from previous studies and reports (44-46). In contrast to our study, a NSW report recorded a lower rate of ED presentations in those living regional/remote areas as compared to those living in major cities (46). The differences may be attributable to major factors that were not adjusted in our analysis such as location of hospitals, number of GP visits, stage at cancer diagnosis and other residual confounding.

Strengths and limitations

This study included whole WA population incident cancer data linked to administrative records of health service use, which allows us to make grounded and representative conclusions for the target population based on higher statistical precision, avoidance of selection bias and negligible loss to follow up. Yet the study has several limitations. Firstly, lack of information on the indications for CT scans prevented us from determining if the CT scans were referred for cancer diagnosis or for other health issues. Therefore, our findings have been reported in terms of use of CT within 12 months before and after a diagnosis of cancer and not in terms resulting from the cancer diagnosis per se. Secondly, CT scans undertaken at two secondary hospitals who contract out their radiology services are not included in the data, hence, the rate of CT use may be underestimated. However, since only a small proportion of the population reside in the two suburbs in question, and we adjusted for place of residence in the analysis

For peer review only

(which was found to be non-significant), the missing data are likely to have minimal impact on the result. Although the data is several years old, to our knowledge this is one of the first studies fully examining the use of CT scans and health care services used around the diagnosis window at the population-based level. As such it provides valuable information for planning health care resources and to identify areas for further investigation of factors driving the growth of use of health services. The results will also provide valuable baseline data for future studies evaluating the impact of international campaigns (i.e., Choosing Wisely) in improving appropriate use in diagnostic testing such as CT scanning.

Conclusion

Our results showed an increase in demand for health services from three months before diagnosis of cancer. While the pattern of use before and after diagnosis remained unchanged for CT, ED and hospitalisations over the study period, differences in the magnitude of use were observed. From diagnosis onwards, the magnitude of the rate of ED presentations and hospitalisations was higher in 2014 compared to the baseline year of 2004 for both cancer types. In a comparison following diagnosis the rate of CT use was significantly higher in 2014 compared to the baseline year 2004 for solid cancer, however it was lower in ALH cancers. The persisting high use of CT during post cancer diagnosis and increased CT use observed in solid cancers may warrant a further investigation of factors driving this change and whether there is any potential over-testing in this group.

For peer review only

Funding

This study was funded with an Australian National Health and Medical Research Council grant, project grant APP1144573. The study funders had no role in the study design, conduct, manuscript writing or decision to submit for publication.

Competing interests

The authors have no conflicts of interest to declare. The institutions of RM, NH, POL, DY, CW, MB and DM received grant funding from the National Medical Research Council of Australia for investigator-initiated research. The funding agreement ensured author independence in designing the study, interpreting the data, writing, and publishing the report.

Author contributions

NTH, SK, MB, JD, DM, DY, PO, RT, & RM contributed to the study design and concept. RM, DY, and NTH contributed to the acquisition of data. RM, SK, DY, CH, MB, JD, DM, PO, CW, DM2, TC and NTH contributed to the analysis and interpretation of the data. NTH, CH, DY, SK, and RM contributed to drafting of the manuscript. RM, JD, DM, PO, DY, CW, DM2, RT and NTH contributed to critical revision of the manuscript for important intellectual content. RM and NTH contributed to statistical expertise. RM, MB, JD, DM, PO secured funding for the study.

Data sharing statement

The data that support the findings of this study are available from the relevant data custodians of the study datasets. Restrictions by the data custodians mean that the data are not publicly available or able to be provided by the authors. Researchers wishing to access the datasets used in this study should refer to the Western Australian Data Linkage Unit and the Australian Institute of Health and Welfare.

For peer review only

Ethics approval and consent to participate

Ethics approval was provided by the Curtin University Human Research Ethics Committee (HRE2017-0822), the Australian Institute of Health and Welfare Human Research Ethics Committee (EO2018/4/485), and the Western Australian Department of Health Human Research Ethics Committee (2011/97).

Patient consent

Not applicable

Acknowledgment

We thank the National Health and Medical Research Council for supporting this work. We acknowledge the Western Australian Data Linkage Unit and custodians of the Hospital Morbidity Data Collection, the WA Cancer Registry, the Emergency Department Data Collection, the WA Death registry and the WA Picture Archiving and Communications System, as well as the individuals whose data enabled this study.

For peer review only

References

1. Johnson CD, Chen M-H, Toledano AY, Heiken JP, Dachman A, Kuo MD, et al. Accuracy of CT colonography for detection of large adenomas and cancers. New England Journal of Medicine. 2008;359(12):1207-17.
2. Team NLSTR. Reduced lung-cancer mortality with low-dose computed tomographic screening. New England Journal of Medicine. 2011;365(5):395-409.
3. Malietzis G, Aziz O, Bagnall N, Johns N, Fearon K, Jenkins J. The role of body composition evaluation by computerized tomography in determining colorectal cancer treatment outcomes: a systematic review. European Journal of Surgical Oncology (EJSO). 2015;41(2):186-96.
4. DeWitt J, Devereaux BM, Lehman GA, Sherman S, Imperiale TF. Comparison of endoscopic ultrasound and computed tomography for the preoperative evaluation of pancreatic cancer: a systematic review. Clinical Gastroenterology and Hepatology. 2006;4(6):717-25.
5. De González AB, Mahesh M, Kim K-P, Bhargavan M, Lewis R, Mettler F, et al. Projected cancer risks from computed tomographic scans performed in the United States in 2007. Archives of internal medicine. 2009;169(22):2071-7.
6. Smith-Bindman R, Lipson J, Marcus R, Kim K-P, Mahesh M, Gould R, et al. Radiation dose associated with common computed tomography examinations and the associated lifetime attributable risk of cancer. Archives of internal medicine. 2009;169(22):2078-86.
7. Pearce MS, Salotti JA, Little MP, McHugh K, Lee C, Kim KP, et al. Radiation exposure from CT scans in childhood and subsequent risk of leukaemia and brain tumours: a retrospective cohort study. The Lancet. 2012;380(9840):499-505.
8. Council NR. Health risks from exposure to low levels of ionizing radiation: BEIR VII phase 2. 2006.
9. McGinnis JM, Stuckhardt L, Saunders R, Smith M. Best care at lower cost: the path to continuously learning health care in America. 2013.
10. Smith-Bindman R, Kwan ML, Marlow EC, Theis MK, Bolch W, Cheng SY, et al. Trends in use of medical imaging in US health care systems and in Ontario, Canada, 2000-2016. Jama. 2019;322(9):843-56.
11. Hess EP, Haas LR, Shah ND, Stroebel RJ, Denham CR, Swensen SJ. Trends in computed tomography utilization rates. Journal of patient safety. 2014;10(1):52-8.
12. Smith-Bindman R, Miglioretti DL, Larson EB. Rising use of diagnostic medical imaging in a large integrated health system. Health affairs. 2008;27(6):1491-502.
13. Pearce MS, Salotti JA, McHugh K, Metcalf W, Kim KP, Craft AW, et al. CT scans in young people in Northern England: trends and patterns 1993–2002. Pediatric radiology. 2011;41(7):832-8.
14. Smith-Bindman R, Miglioretti DL, Johnson E, Lee C, Feigelson HS, Flynn M, et al. Use of diagnostic imaging studies and associated radiation exposure for patients enrolled in large integrated health care systems, 1996-2010. Jama. 2012;307(22):2400-9.
15. Dinan MA, Curtis LH, Hammill BG, Patz EF, Abernethy AP, Shea AM, et al. Changes in the use and costs of diagnostic imaging among Medicare beneficiaries with cancer, 1999-2006. Jama. 2010;303(16):1625-31.
16. Maxwell S, Ha NT, Bulsara MK, Doust J, Mcrobbie D, O'Leary P, et al. Increasing use of CT requested by emergency department physicians in tertiary hospitals in Western Australia 2003–2015: an analysis of linked administrative data. BMJ open. 2021;11(3):e043315.
17. Zhou Y, Abel GA, Hamilton W, Singh H, Walter FM, Lyratzopoulos G. Imaging activity possibly signalling missed diagnostic opportunities in bladder and kidney cancer: a longitudinal data-linkage study using primary care electronic health records. Cancer Epidemiology. 2020;66:101703.
18. Jessen NH, Jensen H, Falborg AZ, Glerup H, Gronbaek H, Vedsted P. Abdominal investigations in the year preceding a diagnosis of abdominal cancer: a register-based cohort study in Denmark. Cancer epidemiology. 2021;72:101926.

For peer review only

19. Purdie S, Creighton N, White KM, Baker D, Ewald D, Lee CK, et al. Pathways to diagnosis of non-small cell lung cancer: a descriptive cohort study. NPJ primary care respiratory medicine. 2019;29(1):1-6.
20. Morrell S, Young J, Roder D. The burden of cancer on primary and secondary health care services before and after cancer diagnosis in New South Wales, Australia. BMC health services research. 2019;19(1):1-8.
21. Christensen KG, Fenger-Grøn M, Flarup KR, Vedsted P. Use of general practice, diagnostic investigations and hospital services before and after cancer diagnosis-a population-based nationwide registry study of 127,000 incident adult cancer patients. BMC health services research. 2012;12(1):1-8.
22. Benchimol EI, Smeeth L, Guttmann A, Harron K, Moher D, Petersen I, et al. The REporting of studies Conducted using Observational Routinely-collected health Data (RECORD) Statement. PLOS Medicine. 2015;12(10):e1001885.
23. Holman CD, Bass AJ, Rouse IL, Hobbs MS. Population-based linkage of health records in Western Australia: development of a health services research linked database. Australian and New Zealand journal of public health. 1999;23(5):453-9.
24. Randall SM, Ferrante AM, Boyd JH, Bauer JK, Semmens JB. Privacy-preserving record linkage on large real world datasets. J Biomed Inform. 2014;50:205-12.
25. World Health Organization. ICD-10 : international statistical classification of diseases and related health problems : tenth revision. 2nd ed ed. Geneva: World Health Organization; 2004.
26. Smith-Bindman R, Miglioretti DL, Larson EB. Rising use of diagnostic medical imaging in a large integrated health system. Health Aff (Millwood). 2008;27(6):1491-502.
27. Christensen D, Davis G, Draper G, Mitrou F, McKeown S, Lawrence D, et al. Evidence for the use of an algorithm in resolving inconsistent and missing Indigenous status in administrative data collections. Australian Journal of Social Issues. 2014;49(4):423-43.
28. Australian Bureau of Statistics. 1270.0.55.005 - Australian Statistical Geography Standard (ASGS): Volume 5 - Remoteness Structure, July 2016 2018 [updated 16/03/2018. Available from: https://www.abs.gov.au/ausstats/abs@.nsf/mf/1270.0.55.005?OpenDocument.
29. Australian Bureau of Statistics. Technical Paper - Socio-economic Indexes for Areas (SEIFA) ABS catalogue number: 2033.0.55.001. Commonwealth of Australia; 2016.
30. Holman CD, Preen DB, Baynham NJ, Finn JC, Semmens JB. A multipurpose comorbidity scoring system performed better than the Charlson index. Journal of clinical epidemiology. 2005;58(10):1006-14.
31. StataCorp. Stata Statistical Software: Release 15. College Station, TX: StataCorp LLC; 2017.
32. Wright CM, Bulsara MK, Norman R, Moorin RE. Increase in computed tomography in Australia driven mainly by practice change: a decomposition analysis. Health Policy. 2017;121(7):823-9.
33. Møller M, Juvik B, Olesen SC, Sandstrøm H, Laxafoss E, Reuter SB, et al. Diagnostic property of direct referral from general practitioners to contrast-enhanced thoracoabdominal CT in patients with serious but non-specific symptoms or signs of cancer: a retrospective cohort study on cancer prevalence after 12 months. BMJ Open. 2019;9(12):e032019.
34. Guldbrandt LM. The effect of direct referral for fast CT scan in early lung cancer detection in general practice. A clinical, cluster-randomised trial. Dan Med J. 2015;62(3).
35. Lowthian JA, Curtis AJ, Cameron PA, Stoelwinder JU, Cooke MW, McNeil JJ. Systematic review of trends in emergency department attendances: an Australian perspective. Emergency medicine journal : EMJ. 2011;28(5):373-7.
36. Mohr P, Eggermont AMM, Hauschild A, Buzaid A. Staging of cutaneous melanoma. Ann Oncol. 2009;20 Suppl 6(Suppl 6):vi14-vi21.
37. Zienius K, Chak-Lam I, Park J, Ozawa M, Hamilton W, Weller D, et al. Direct access CT for suspicion of brain tumour: an analysis of referral pathways in a population-based patient group. BMC Family Practice. 2019;20(1):118.

For peer review only

38. Hansen PL, Hjertholm P, Vedsted P. Increased diagnostic activity in general practice during the year preceding colorectal cancer diagnosis. International Journal of Cancer. 2015;137(3):615-24.
39. Guldbrandt LM, Møller H, Jakobsen E, Vedsted P. General practice consultations, diagnostic investigations, and prescriptions in the year preceding a lung cancer diagnosis. Cancer medicine. 2017;6(1):79-88.
40. Armitage JO, Gascoyne RD, Lunning MA, Cavalli F. Non-hodgkin lymphoma. The lancet. 2017;390(10091):298-310.
41. Connors JM. State-of-the-art therapeutics: Hodgkin's lymphoma. Journal of clinical oncology. 2005;23(26):6400-8.
42. Vinnicombe SJ, Reznek RH. Computerised tomography in the staging of Hodgkin's disease and non-Hodgkin's lymphoma. European journal of nuclear medicine and molecular imaging. 2003;30(1):S42-S55.
43. Lau WFE, Binns DS, Ware RE, Ramdave S, Pitman AG, Hicks RJ. Clinical experience with the first combined positron emission tomography/computed tomography scanner in Australia. Medical Journal of Australia. 2005;182(4):172-6.
44. Wong TH, Lau ZY, Ong WS, Tan KB, Wong YJ, Farid M, et al. Cancer patients as frequent attenders in emergency departments: a national cohort study. Cancer medicine. 2018;7(9):4434-46.
45. Unwin M, Crisp E, Stankovich J, McCann D, Kinsman L. Socioeconomic disadvantage as a driver of non-urgent emergency department presentations: a retrospective data analysis. PLoS One. 2020;15(4):e0231429.
46. Bureau of Health Information. The Insights Series: Emergency department utilisation by people with cancer Sydney (NSW); BHI; 2014.

For peer review only

List of tables, Figures and Appendix

Tables

Table 1. Characteristics of the Study Population

Table 2. Adjusted rate of CT scanning, ED presentations and Hospitalisations in 12 months before and after cancer diagnosis

Figures

Figure 1. Adjusted rate of CT scanning, ED presentations and hospitalisation in months before and after diagnosis by cancer groups in 2004 and 2014

Figure 2. Adjusted rate of total CT scans, ED presentations and hospitalisations in a year before and after cancer diagnosis over study period by cancer groups

Appendices

Appendix A. Multivariable negative binomial regression of monthly use of CT scanning, ED presentation, and hospitalisations within 12 Months before and after Cancer Diagnosis over the study period.

Appendix B. Trends in use of CT scanning, ED presentation, and hospitalisations in diagnosis window across sub-cancer types in 2004 and 2014

Appendix C. Multivariable negative binomial regression of total use of CT scanning, ED presentation, and hospitalisations in a year before diagnosis with cancer over the study period

Appendix D. Multivariable negative binomial regression of total use of CT scanning, ED presentation, and hospitalisations in a year after diagnosis with cancer over the study period

For peer review only

For peer review only

For peer review only

30

1

For peer review only

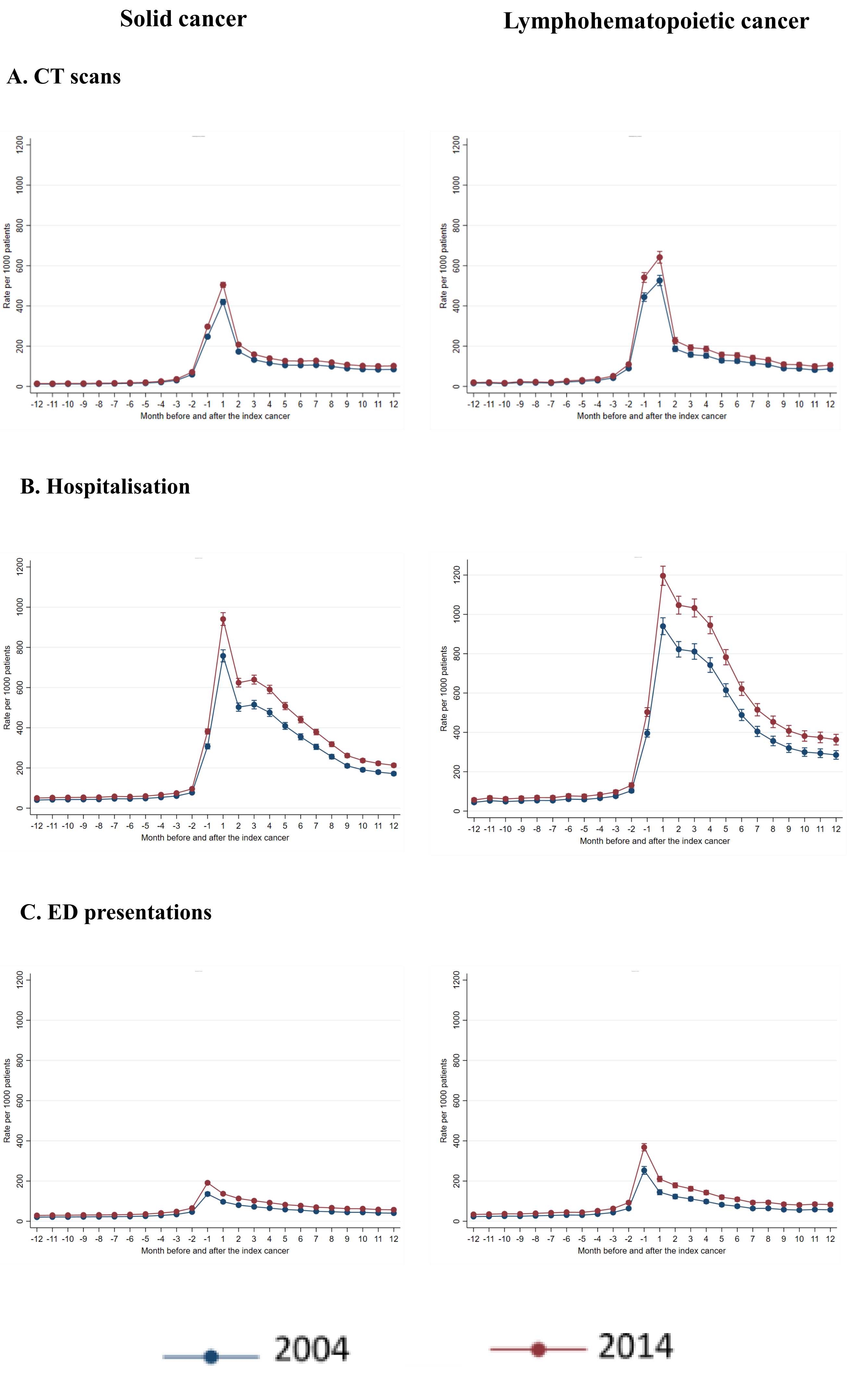

Figure 1. Adjusted rate of CT scanning, hospitalisation and ED presentations in months before and after diagnosis by cancer groups in 2004 and 2014

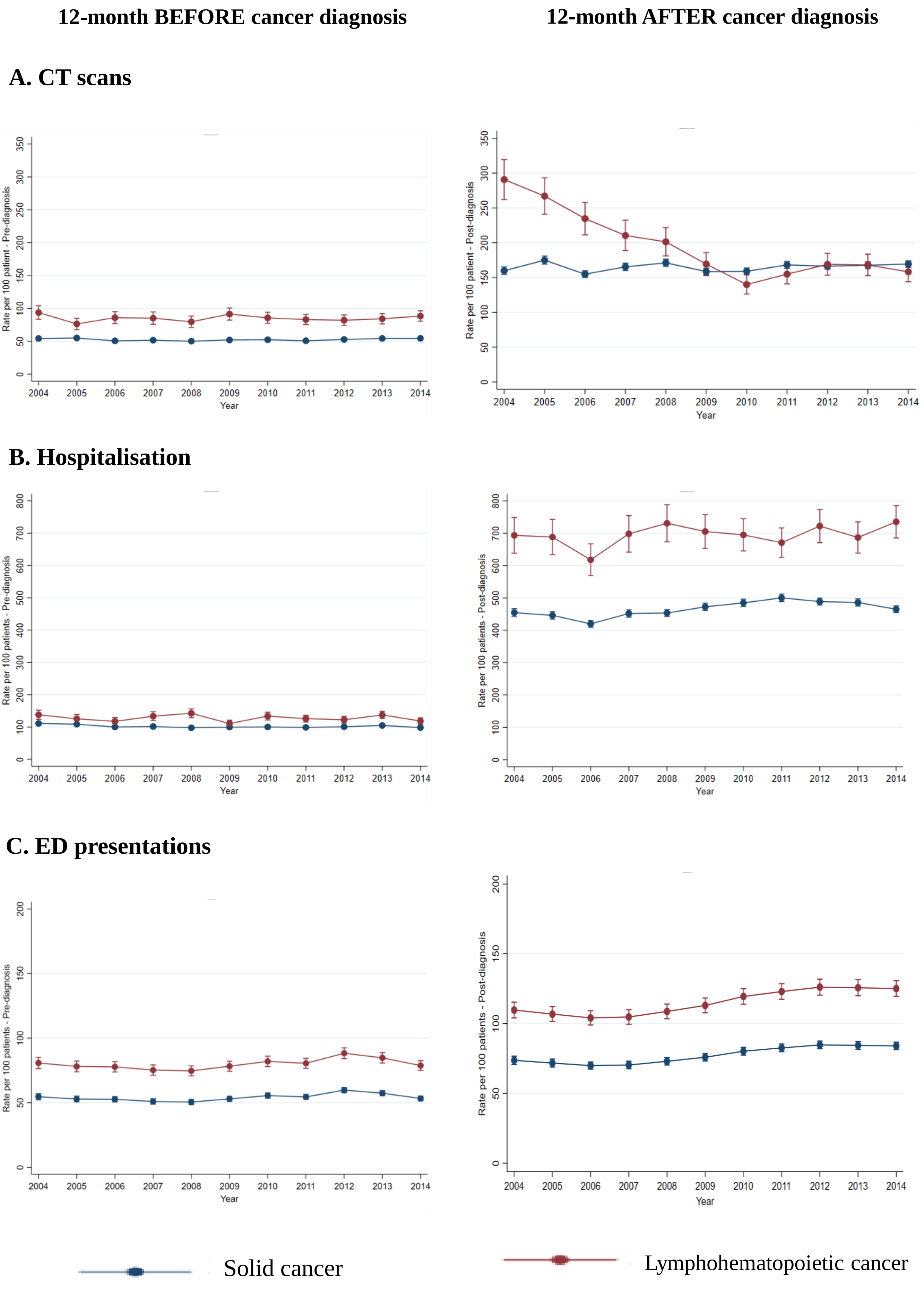

Figure 2. Adjusted rate of total CT scans, hospitalisations and ED presentations in a year before and after cancer diagnosis over study period by cancer groups

Appendix

The use of computed tomography, ED presentation and hospitalisations 12-months before and after a diagnosis of cancer in Western Australia: A population-based retrospective cohort study

Author information

Ninh T. Ha[1], PhD, thi.ha@curtin.edu.au

Sviatlana Kamarova[1], PhD, sviatlana.kamarova@curtin.edu.au

David Youens[1], david.youens@curtin.edu.au

Chau Ho[1,] chau.ho@curtin.edu.au

Max Bulsara [2,3], PhD, max.bulsara@nd.edu.au

Jenny Doust[4], BMBS, PhD, j.doust@uq.edu.au

Donald McRobbie[5], PhD, donald.mcrobbie@adelaide.edu.au

Peter O'Leary [1,6,7], PhD, peter.oleary@health.wa.gov.au

Cameron Wright[1,8,9,10], MD, cameron.wright@curtin.edu.au

Richard Trevithick[11], MD, richard.trevithick@health.wa.gov.au

Rachael Moorin,[1,3] PhD, R.Moorin@curtin.edu.au

Affiliations

1. Health Economics and Data Analytics, Curtin School of Population Health, Faculty of Health Sciences, Curtin University, Western Australia;

2. Institute for Health Research, University of Notre Dame, Western Australia;

For peer review only

3. Centre for Health Services Research, School of Population and Global Health, The University of Western Australia;

4. Centre for Longitudinal and Life Course Research, Faculty of Medicine, University of Queensland;

5. School of Physical Sciences, University of Adelaide;

6. Obstetrics and Gynaecology Medical School, Faculty of Health and Medical Sciences, The University of Western Australia, Perth, Western Australia, Australia;

7. PathWest Laboratory Medicine, QE2 Medical Centre, Nedlands, Western Australia, Australia;

8. Fiona Stanley Hospital, 11 Robin Warren Dr, Murdoch, Western Australia, Australia;

9. Division of Internal Medicine, Medical School, Faculty of Health and Medical Sciences, The University of Western Australia;

10. School of Medicine, College of Health and Medicine, University of Tasmania, Hobart, Tasmania;

11. Western Australian Cancer Registry, Clinical Excellence Division, Department of Health, East Perth, Western Australia.

***Corresponding author:**

Ninh Thi Ha

Health Economics & Data Analytics, Curtin School of Population Health, Faculty of Health Sciences, Curtin University

GPO Box U1987, Perth, Western Australia, 6845

Email: Thi.Ha@curtin.edu.au; ninh.ha05@gmail.com

For peer review only

Phone: +61 08 9266 5134

For peer review only

Appendix A. Multivariable negative binomial regression of monthly use of CT scanning, ED presentation, and hospitalisations within 12 Months before and after Cancer Diagnosis over the study period.

	CT scan		Hospitalisations		ED presentations	
	RR	95%CI	RR	95%CI	RR	95%CI
**Month pre& post cancer diagnosis**						
-12	1	(1; 1)	1	(1; 1)	1	(1; 1)
-11	1	(0.92; 1.09)	1.04*	(1.00; 1.08)	1.03	(0.97; 1.10)
-10	1.04	(0.96; 1.14)	1.05**	(1.01; 1.10)	1.04	(0.98; 1.10)
-9	1.04	(0.95; 1.13)	1.07**	(1.02; 1.11)	1.07*	(1.01; 1.14)
-8	1.12**	(1.03; 1.21)	1.07***	(1.03; 1.12)	1.09**	(1.02; 1.15)
-7	1.17***	(1.08; 1.28)	1.16***	(1.11; 1.21)	1.12***	(1.05; 1.19)
-6	1.27***	(1.17; 1.37)	1.14***	(1.09; 1.19)	1.16***	(1.09; 1.23)
-5	1.40***	(1.29; 1.52)	1.19***	(1.14; 1.25)	1.24***	(1.17; 1.31)
-4	1.75***	(1.62; 1.89)	1.32***	(1.27; 1.38)	1.41***	(1.33; 1.50)
-3	2.52***	(2.34; 2.71)	1.49***	(1.43; 1.55)	1.66***	(1.57; 1.76)
-2	4.94***	(4.61; 5.28)	1.91***	(1.82; 1.99)	2.26***	(2.14; 2.39)
-1	20.6***	(19.3; 21.9)	7.57***	(7.22; 7.94)	6.62***	(6.30; 6.95)
1	34.9***	(32.8; 37.2)	18.7***	(17.8; 19.6)	4.74***	(4.51; 4.98)
2	14.4***	(13.5; 15.4)	12.4***	(11.8; 13.0)	3.92***	(3.72; 4.12)
3	11.0***	(10.3; 11.8)	12.7***	(12.1; 13.3)	3.52***	(3.35; 3.71)
4	9.67***	(9.05; 10.3)	11.7***	(11.2; 12.3)	3.19***	(3.03; 3.36)
5	8.79***	(8.22; 9.39)	10.1***	(9.61; 10.6)	2.85***	(2.70; 3.01)
6	8.75***	(8.19; 9.36)	8.75***	(8.33; 9.18)	2.68***	(2.54; 2.83)
7	8.85***	(8.28; 9.46)	7.52***	(7.16; 7.89)	2.41***	(2.28; 2.55)
8	8.26***	(7.73; 8.83)	6.31***	(6.01; 6.63)	2.31***	(2.19; 2.45)
9	7.48***	(6.99; 8.01)	5.19***	(4.95; 5.45)	2.17***	(2.05; 2.30)
10	7.10***	(6.63; 7.59)	4.70***	(4.48; 4.94)	2.16***	(2.04; 2.29)
11	6.99***	(6.53; 7.48)	4.42***	(4.21; 4.65)	2.01***	(1.90; 2.13)
12	7.07***	(6.61; 7.57)	4.23***	(4.03; 4.44)	1.97***	(1.86; 2.09)
**Cancer group**						
Solid cancer	1	(1; 1)	1	(1; 1)	1	(1; 1)
ALH cancer[†]	1.25*	(1.02; 1.53)	0.99	(0.84; 1.17)	1.02	(0.87; 1.20)
**Interaction term of months # cancer groups**						
-12 # solid cancer	1	(1; 1)	1	(1; 1)	1	(1; 1)
-12 # ALH	1	(1; 1)	1	(1; 1)	1	(1; 1)
-11 # solid cancer	1	(1; 1)	1	(1; 1)	1	(1; 1)
-11 # ALH	1.02	(0.77; 1.34)	1.14*	(1.02; 1.29)	1	(0.82; 1.22)
-10 # solid cancer	1	(1; 1)	1	(1; 1)	1	(1; 1)
-10 # ALH	0.85	(0.64; 1.11)	1.04	(0.92; 1.16)	1.05	(0.87; 1.27)
-9 # solid cancer	1	(1; 1)	1	(1; 1)	1	(1; 1)
-9 # ALH	1.14	(0.88; 1.48)	1.09	(0.97; 1.22)	1	(0.83; 1.21)
-8 # solid cancer	1	(1; 1)	1	(1; 1)	1	(1; 1)
-8 # ALH	1.02	(0.78; 1.32)	1.13*	(1.00; 1.27)	1.07	(0.89; 1.28)
-7 # solid cancer	1	(1; 1)	1	(1; 1)	1	(1; 1)
-7 # ALH	0.88	(0.67; 1.14)	1.04	(0.92; 1.18)	1.11	(0.91; 1.35)

For peer review only

	CT scan		Hospitalisations		ED presentations	
	RR	95%CI	RR	95%CI	RR	95%CI
-6 # solid cancer	1	(1; 1)	1	(1; 1)	1	(1; 1)
-6 # ALH	1.08	(0.84; 1.38)	1.20**	(1.06; 1.35)	1.14	(0.95; 1.38)
-5 # solid cancer	1	(1; 1)	1	(1; 1)	1	(1; 1)
-5 # ALH	1.11	(0.87; 1.43)	1.11	(0.98; 1.26)	1.05	(0.87; 1.28)
-4 # solid cancer	1	(1; 1)	1	(1; 1)	1	(1; 1)
-4 # ALH	1.04	(0.82; 1.33)	1.12	(0.98; 1.27)	1.08	(0.90; 1.31)
-3 # solid cancer	1	(1; 1)	1	(1; 1)	1	(1; 1)
-3 # ALH	1.02	(0.81; 1.30)	1.15*	(1.01; 1.31)	1.12	(0.94; 1.33)
-2 # solid cancer	1	(1; 1)	1	(1; 1)	1	(1; 1)
-2 # ALH	1.11	(0.89; 1.38)	1.22**	(1.06; 1.40)	1.21*	(1.02; 1.43)
-1 # solid cancer	1	(1; 1)	1	(1; 1)	1	(1; 1)
-1 # ALH	1.31**	(1.07; 1.61)	1.17*	(1.00; 1.38)	1.63***	(1.39; 1.92)
1 # solid cancer	1	(1; 1)	1	(1; 1)	1	(1; 1)
1 # ALH	0.92	(0.74; 1.13)	1.13	(0.96; 1.34)	1.30**	(1.11; 1.54)
2 # solid cancer	1	(1; 1)	1	(1; 1)	1	(1; 1)
2 # ALH	0.79*	(0.64; 0.97)	1.49***	(1.26; 1.76)	1.34***	(1.13; 1.59)
3 # solid cancer	1	(1; 1)	1	(1; 1)	1	(1; 1)
3 # ALH	0.87	(0.70; 1.08)	1.44***	(1.22; 1.70)	1.35***	(1.14; 1.60)
4 # solid cancer	1	(1; 1)	1	(1; 1)	1	(1; 1)
4 # ALH	0.96	(0.77; 1.19)	1.42***	(1.21; 1.68)	1.31**	(1.11; 1.56)
5 # solid cancer	1	(1; 1)	1	(1; 1)	1	(1; 1)
5 # ALH	0.89	(0.72; 1.11)	1.37***	(1.16; 1.62)	1.23*	(1.04; 1.47)
6 # solid cancer	1	(1; 1)	1	(1; 1)	1	(1; 1)
6 # ALH	0.88	(0.70; 1.09)	1.26**	(1.06; 1.48)	1.19*	(1.00; 1.42)
7 # solid cancer	1	(1; 1)	1	(1; 1)	1	(1; 1)
7 # ALH	0.80*	(0.64; 0.99)	1.21*	(1.02; 1.43)	1.13	(0.95; 1.35)
8 # solid cancer	1	(1; 1)	1	(1; 1)	1	(1; 1)
8 # ALH	0.79*	(0.63; 0.99)	1.27**	(1.07; 1.50)	1.19	(0.99; 1.42)
9 # solid cancer	1	(1; 1)	1	(1; 1)	1	(1; 1)
9 # ALH	0.73**	(0.58; 0.91)	1.39***	(1.17; 1.64)	1.14	(0.95; 1.37)
10 # solid cancer	1	(1; 1)	1	(1; 1)	1	(1; 1)
10 # ALH	0.76*	(0.60; 0.95)	1.43***	(1.21; 1.70)	1.1	(0.92; 1.33)
11 # solid cancer	1	(1; 1)	1	(1; 1)	1	(1; 1)
11 # ALH	0.71**	(0.57; 0.90)	1.49***	(1.26; 1.77)	1.24*	(1.03; 1.49)
12 # solid cancer	1	(1; 1)	1	(1; 1)	1	(1; 1)
12 # ALH	0.75*	(0.59; 0.94)	1.52***	(1.28; 1.80)	1.23*	(1.03; 1.49)
**Year of cancer diagnosis**						
2004	1	(1; 1)	1	(1; 1)	1	(1; 1)
2005	1.05*	(1.00; 1.09)	0.98	(0.93; 1.04)	0.96	(0.89; 1.04)
2006	0.94**	(0.90; 0.98)	0.91**	(0.86; 0.96)	0.94	(0.87; 1.02)
2007	0.96	(0.93; 1.00)	0.98	(0.92; 1.03)	0.93	(0.86; 1.00)
2008	0.97	(0.93; 1.00)	0.97	(0.91; 1.02)	0.94	(0.87; 1.01)
2009	0.92***	(0.88; 0.95)	0.99	(0.94; 1.05)	0.98	(0.91; 1.06)
2010	0.91***	(0.88; 0.95)	1.02	(0.96; 1.07)	1.03	(0.95; 1.11)

For peer review only

	CT scan		Hospitalisations		ED presentations	
	RR	95%CI	RR	95%CI	RR	95%CI
2011	0.94**	(0.90; 0.97)	1.02	(0.97; 1.07)	1.04	(0.96; 1.12)
2012	0.95**	(0.92; 0.99)	1.02	(0.97; 1.08)	1.09*	(1.01; 1.18)
2013	0.96*	(0.93; 1.00)	1.03	(0.97; 1.09)	1.07	(0.99; 1.16)
2014	0.96	(0.93; 1.00)	0.97	(0.92; 1.03)	1.04	(0.96; 1.12)
**Sex**						
Male	1	(1; 1)	1	(1; 1)	1	(1; 1)
Female	1	(0.99; 1.02)	1.13***	(1.11; 1.16)	0.98*	(0.96; 1.00)
**Age- group at diagnosis**						
18-45 years	1	(1; 1)	1	(1; 1)	1	(1; 1)
46-65 years	1.02	(0.99; 1.05)	0.96**	(0.93; 0.99)	0.76***	(0.73; 0.79)
66-75 years	0.96**	(0.93; 0.99)	0.84***	(0.81; 0.87)	0.73***	(0.70; 0.76)
>75 years	0.74***	(0.72; 0.77)	0.66***	(0.63; 0.68)	0.84***	(0.80; 0.87)
**Socio-economic status**[‡]						
Least disadvantage	1	(1; 1)	1	(1; 1)	1	(1; 1)
Less disadvantage	1.08***	(1.05; 1.11)	1.01	(0.98; 1.05)	1.30***	(1.26; 1.35)
Moderate disadvantage	1.07***	(1.05; 1.10)	1.01	(0.98; 1.04)	1.45***	(1.41; 1.49)
High disadvantage	1.12***	(1.10; 1.15)	1.01	(0.98; 1.05)	1.65***	(1.60; 1.69)
Highest disadvantage	1.12***	(1.08; 1.15)	0.99	(0.94; 1.03)	1.75***	(1.67; 1.84)
Unknown	1.1	(0.97; 1.26)	1.06	(0.81; 1.40)	1.12	(0.94; 1.33)
**Accessibility to services**[§]						
Major cities	1	(1; 1)	1	(1; 1)	1	(1; 1)
Inner regional	0.87***	(0.85; 0.89)	0.96**	(0.93; 0.99)	1.19***	(1.15; 1.22)
Outer regional	0.84***	(0.81; 0.86)	0.91***	(0.87; 0.94)	1.52***	(1.47; 1.58)
Remote	0.84***	(0.79; 0.91)	0.83***	(0.78; 0.88)	1.69***	(1.58; 1.81)
Very remote	0.87***	(0.83; 0.91)	0.83***	(0.78; 0.89)	2.33***	(2.21; 2.47)
Unknown	0.96	(0.75; 1.23)	0.67**	(0.50; 0.90)	1.03	(0.76; 1.39)
**Comorbidities**[¶]						
0	1	(1; 1)	1	(1; 1)	1	(1; 1)
1-2	1.87***	(1.75; 2.00)	3.74***	(3.44; 4.06)	1.19***	(1.11; 1.29)
3-5	3.00***	(2.81; 3.21)	6.58***	(6.06; 7.15)	2.23***	(2.07; 2.40)
6+	4.94***	(4.62; 5.27)	11.5***	(10.5; 12.5)	5.54***	(5.15; 5.96)
**Basis of diagnosis**						
Non-pathological	1	(1; 1)	1	(1; 1)	1	(1; 1)
Pathological	0.95*	(0.91; 0.99)	1.08	(0.96; 1.20)	0.66***	(0.63; 0.69)
**Hospital catchment region**						
Other	1	(1; 1)	1	(1; 1)	1	(1; 1)
Bentley and Armadale	1.04***	(1.02; 1.07)	1.01	(0.98; 1.05)	0.99	(0.95; 1.03)

* p <0.05, ** p <0.01, *** p <0.001

CT: Computed tomography, ED: emergency department, IRR: incidence rate ratio, ALH: All haematopoietic cancer.

For peer review only

RRs were also adjusted for interactions between time in months before and after cancer diagnosis and cancer groups; time in years of cancer diagnosis cohort and cancer groups; and Indigenous status. Please note Indigenous status cannot be reported as per restrictions imposed by the human research ethics approval and can only be used for model adjustment purposes. Month post diagnosis = 1 includes the day diagnosed.

For peer review only

Appendix C. Multivariable negative binomial regression of total use of CT scanning, ED presentation, and hospitalisations in a year before diagnosis with cancer over the study period

	CT scan		Hospitalisations		ED presentations	
	RR	95%CI	RR	95%CI	RR	95%CI
**Cancer group**						
Solid cancer	1	(1; 1)	1	(1; 1)	1	(1; 1)
ALH cancer	1.56***	(1.39; 1.75)	1.07	(0.96; 1.19)	1.31***	(1.14; 1.50)
**Year of cancer diagnosis**						
2004	1	(1; 1)	1	(1; 1)	1	(1; 1)
2005	1.01	(0.96; 1.07)	0.98	(0.93; 1.03)	0.97	(0.91; 1.04)
2006	0.93*	(0.88; 0.99)	0.90***	(0.86; 0.95)	0.96	(0.90; 1.02)
2007	0.95	(0.90; 1.01)	0.91***	(0.87; 0.96)	0.93*	(0.88; 1.00)
2008	0.92**	(0.88; 0.98)	0.88***	(0.84; 0.92)	0.93*	(0.87; 0.99)
2009	0.96	(0.91; 1.01)	0.90***	(0.86; 0.94)	0.97	(0.92; 1.04)
2010	0.97	(0.92; 1.02)	0.90***	(0.86; 0.94)	1.02	(0.96; 1.09)
2011	0.94*	(0.89; 0.99)	0.89***	(0.85; 0.93)	1.00	(0.94; 1.06)
2012	0.97	(0.92; 1.03)	0.91***	(0.87; 0.95)	1.10**	(1.04; 1.16)
2013	1.00	(0.95; 1.06)	0.94*	(0.90; 0.99)	1.05	(0.99; 1.11)
2014	1.00	(0.95; 1.06)	0.88***	(0.85; 0.92)	0.97	(0.91; 1.03)
**Interaction term of cancer diagnosis year and cancer type**						
2004 # solid cancer	1	(1; 1)	1	(1; 1)	1	(1; 1)
2004 # ALH	1	(1; 1)	1	(1; 1)	1	(1; 1)
2005 # solid cancer	1	(1; 1)	1	(1; 1)	1	(1; 1)
2005 # ALH	0.80*	(0.68; 0.95)	0.93	(0.80; 1.08)	0.93	(0.77; 1.13)
2006 # solid cancer	1	(1; 1)	1	(1; 1)	1	(1; 1)
2006 # ALH	0.98	(0.83; 1.16)	0.94	(0.81; 1.10)	1.03	(0.85; 1.25)
2007 # solid cancer	1	(1; 1)	1	(1; 1)	1	(1; 1)
2007 # ALH	0.95	(0.81; 1.12)	1.06	(0.91; 1.23)	0.98	(0.81; 1.19)
2008 # solid cancer	1	(1; 1)	1	(1; 1)	1	(1; 1)
2008 # ALH	0.92	(0.78; 1.08)	1.18*	(1.01; 1.36)	0.95	(0.79; 1.15)
2009 # solid cancer	1	(1; 1)	1	(1; 1)	1	(1; 1)
2009 # ALH	1.02	(0.87; 1.19)	0.90	(0.77; 1.04)	0.95	(0.79; 1.14)
2010 # solid cancer	1	(1; 1)	1	(1; 1)	1	(1; 1)
2010 # ALH	0.95	(0.81; 1.11)	1.08	(0.93; 1.24)	0.94	(0.78; 1.12)
2011 # solid cancer	1	(1; 1)	1	(1; 1)	1	(1; 1)
2011 # ALH	0.95	(0.81; 1.10)	1.03	(0.89; 1.18)	0.97	(0.82; 1.16)
2012 # solid cancer	1	(1; 1)	1	(1; 1)	1	(1; 1)
2012 # ALH	0.90	(0.77; 1.05)	0.98	(0.85; 1.13)	0.96	(0.80; 1.14)
2013 # solid cancer	1	(1; 1)	1	(1; 1)	1	(1; 1)
2013 # ALH	0.90	(0.77; 1.05)	1.06	(0.92; 1.21)	1.02	(0.86; 1.22)
2014 # solid cancer	1	(1; 1)	1	(1; 1)	1	(1; 1)
2014 # ALH	0.94	(0.81; 1.09)	0.97	(0.85; 1.12)	1.07	(0.90; 1.27)
**Sex**						
Male	1	(1; 1)	1	(1; 1)	1	(1; 1)
Female	0.94***	(0.92; 0.96)	0.78***	(0.77; 0.79)	0.92***	(0.90; 0.94)

For peer review only

	CT scan		Hospitalisations		ED presentations	
	RR	95%CI	RR	95%CI	RR	95%CI
**Age- group at diagnosis**						
18-45 years	1	(1; 1)	1	(1; 1)	1	(1; 1)
46-65 years	1.00	(0.96; 1.04)	1.05**	(1.01; 1.09)	0.70***	(0.68; 0.73)
66-75 years	1.07**	(1.02; 1.11)	1.25***	(1.21; 1.30)	0.68***	(0.65; 0.71)
>75 years	0.94**	(0.91; 0.98)	1.38***	(1.34; 1.44)	0.86***	(0.82; 0.89)
**Socio-economic status**						
Least disadvantage	1	(1; 1)	1	(1; 1)	1	(1; 1)
Less disadvantage	1.07***	(1.03; 1.10)	1.03*	(1.01; 1.06)	1.28***	(1.23; 1.33)
Moderate disadvantage	1.09***	(1.06; 1.12)	1.04**	(1.01; 1.07)	1.43***	(1.38; 1.48)
High disadvantage	1.15***	(1.11; 1.18)	1.04***	(1.02; 1.07)	1.60***	(1.55; 1.66)
Highest disadvantage	1.15***	(1.10; 1.19)	1.01	(0.98; 1.04)	1.72***	(1.65; 1.79)
Unknown	1.22*	(1.01; 1.47)	1.53***	(1.31; 1.77)	0.91	(0.72; 1.14)
**Accessibility to services**						
Major cities	1	(1; 1)	1	(1; 1)	1	(1; 1)
Inner regional	0.84***	(0.82; 0.87)	0.97*	(0.95; 1.00)	1.12***	(1.09; 1.16)
Outer regional	0.81***	(0.77; 0.84)	0.92***	(0.89; 0.96)	1.46***	(1.40; 1.52)
Remote	0.75***	(0.69; 0.83)	0.84***	(0.77; 0.91)	1.69***	(1.55; 1.84)
Very remote	0.78***	(0.74; 0.84)	0.85***	(0.81; 0.90)	2.49***	(2.35; 2.63)
Unknown	0.86	(0.62; 1.19)	0.41***	(0.30; 0.58)	1.10	(0.74; 1.64)
**Number of comorbidities**						
0	1	(1; 1)	1	(1; 1)	1	(1; 1)
1-2	2.28***	(2.08; 2.49)	180.9***	(90.4; 362.2)	1.20***	(1.10; 1.31)
3-5	3.93***	(3.60; 4.29)	430.7***	(215.2; 861.7)	2.62***	(2.41; 2.85)
6+	6.76***	(6.20; 7.37)	1026.5***	(513.0; 2053.8)	6.74***	(6.21; 7.32)
**Basis of diagnosis**						
Non-pathological	1	(1; 1)	1	(1; 1)	1	(1; 1)
Pathological	0.96	(0.91; 1.01)	0.95*	(0.91; 0.99)	0.65***	(0.62; 0.68)
**Hospital catchment region**						
Other	1	(1; 1)	1	(1; 1)	1	(1; 1)
Bentley and Armadale	1.03*	(1.00; 1.06)	0.99	(0.97; 1.02)	0.99	(0.96; 1.02)

* *p* <0.05, ** *p* <0.01, *** *p* <0.001

CT: Computed tomography, ED: emergency department, RR: incidence rate ratio, ALH: All haematopoietic cancer.

RRs were also adjusted for interactions between time in months before and after cancer diagnosis and cancer groups; time in years of cancer diagnosis cohort and cancer groups; and Indigenous status. Please note Indigenous status cannot be reported as per restrictions imposed by the human research ethics approval and can only be used for model adjustment purposes. Month post diagnosis = 1 includes the day diagnosed..

For peer review only

Appendix D. Multivariable negative binomial regression of total use of CT scanning, ED presentation, and hospitalisations in a year after diagnosis with cancer over the study period

	CT scan		Hospitalisations		ED presentations	
	RR	95%CI	RR	95%CI	RR	95%CI
**Cancer group**						
Solid cancer	1	(1; 1)	1	(1; 1)	1	(1; 1)
ALH cancer	1.70***	(1.53; 1.89)	1.43***	(1.31; 1.55)	1.31***	(1.15; 1.49)
**Year of cancer diagnosis**						
2004	1	(1; 1)	1	(1; 1)	1	(1; 1)
2005	1.09***	(1.04; 1.15)	0.98	(0.95; 1.02)	0.97	(0.91; 1.03)
2006	0.97	(0.93; 1.01)	0.92***	(0.89; 0.96)	0.95	(0.90; 1.01)
2007	1.03	(0.99; 1.08)	0.99	(0.96; 1.03)	0.95	(0.89; 1.01)
2008	1.07**	(1.02; 1.12)	1.00	(0.96; 1.03)	0.99	(0.94; 1.05)
2009	0.99	(0.95; 1.04)	1.04*	(1.00; 1.08)	1.02	(0.97; 1.08)
2010	0.99	(0.95; 1.04)	1.07***	(1.03; 1.10)	1.08**	(1.02; 1.14)
2011	1.05*	(1.01; 1.10)	1.10***	(1.06; 1.14)	1.12***	(1.06; 1.19)
2012	1.04	(1.00; 1.09)	1.08***	(1.04; 1.11)	1.16***	(1.10; 1.22)
2013	1.05*	(1.00; 1.10)	1.07***	(1.03; 1.11)	1.14***	(1.08; 1.21)
2014	1.06**	(1.01; 1.11)	1.02	(0.99; 1.06)	1.14***	(1.08; 1.20)
**Interaction term of cancer diagnosis year and cancer type**						
2004 # solid cancer	1	(1; 1)	1	(1; 1)	1	(1; 1)
2004 # ALH	1	(1; 1)	1	(1; 1)	1	(1; 1)
2005 # solid cancer	1	(1; 1)	1	(1; 1)	1	(1; 1)
2005 # ALH	0.84*	(0.72; 0.97)	1.01	(0.90; 1.14)	1.04	(0.87; 1.25)
2006 # solid cancer	1	(1; 1)	1	(1; 1)	1	(1; 1)
2006 # ALH	0.83*	(0.72; 0.96)	0.96	(0.86; 1.09)	0.93	(0.78; 1.12)
2007 # solid cancer	1	(1; 1)	1	(1; 1)	1	(1; 1)
2007 # ALH	0.70***	(0.60; 0.81)	1.01	(0.90; 1.14)	1.06	(0.89; 1.28)
2008 # solid cancer	1	(1; 1)	1	(1; 1)	1	(1; 1)
2008 # ALH	0.65***	(0.56; 0.75)	1.06	(0.94; 1.19)	0.99	(0.82; 1.18)
2009 # solid cancer	1	(1; 1)	1	(1; 1)	1	(1; 1)
2009 # ALH	0.59***	(0.51; 0.68)	0.98	(0.87; 1.10)	1.07	(0.90; 1.28)
2010 # solid cancer	1	(1; 1)	1	(1; 1)	1	(1; 1)
2010 # ALH	0.48***	(0.42; 0.56)	0.94	(0.84; 1.05)	1.09	(0.92; 1.29)
2011 # solid cancer	1	(1; 1)	1	(1; 1)	1	(1; 1)
2011 # ALH	0.51***	(0.44; 0.58)	0.88*	(0.79; 0.98)	0.99	(0.84; 1.17)
2012 # solid cancer	1	(1; 1)	1	(1; 1)	1	(1; 1)
2012 # ALH	0.56***	(0.48; 0.64)	0.97	(0.87; 1.08)	0.92	(0.78; 1.09)
2013 # solid cancer	1	(1; 1)	1	(1; 1)	1	(1; 1)
2013 # ALH	0.55***	(0.48; 0.63)	0.93	(0.83; 1.04)	1.03	(0.87; 1.21)
2014 # solid cancer	1	(1; 1)	1	(1; 1)	1	(1; 1)
2014 # ALH	0.51***	(0.45; 0.59)	1.04	(0.93; 1.16)	1.03	(0.87; 1.21)
**Sex**						
Male	1	(1; 1)	1	(1; 1)	1	(1; 1)

For peer review only

	CT scan		Hospitalisations		ED presentations	
	RR	95%CI	RR	95%CI	RR	95%CI
Female	1.04***	(1.03; 1.06)	1.24***	(1.22; 1.26)	1.03**	(1.01; 1.06)
**Age- group at diagnosis**						
18-45 years	1	(1; 1)	1	(1; 1)	1	(1; 1)
46-65 years	0.98	(0.95; 1.01)	0.94***	(0.91; 0.96)	0.77***	(0.74; 0.80)
66-75 years	0.85***	(0.83; 0.88)	0.75***	(0.73; 0.76)	0.73***	(0.70; 0.76)
>75 years	0.59***	(0.57; 0.61)	0.47***	(0.46; 0.49)	0.74***	(0.71; 0.77)
**Socio-economic status**						
Least disadvantage	1	(1; 1)	1	(1; 1)	1	(1; 1)
Less disadvantage	1.07***	(1.04; 1.10)	1.00	(0.98; 1.03)	1.32***	(1.28; 1.37)
Moderate disadvantage	1.05***	(1.02; 1.08)	1.00	(0.98; 1.03)	1.49***	(1.45; 1.54)
High disadvantage	1.08***	(1.05; 1.11)	1.00	(0.98; 1.02)	1.69***	(1.63; 1.74)
Highest disadvantage	1.07***	(1.04; 1.11)	0.97*	(0.94; 0.99)	1.76***	(1.69; 1.83)
Unknown	1.04	(0.89; 1.22)	0.93	(0.83; 1.06)	1.32**	(1.09; 1.60)
**Accessibility to services**						
Major cities	1	(1; 1)	1	(1; 1)	1	(1; 1)
Inner regional	0.87***	(0.85; 0.90)	0.95***	(0.93; 0.97)	1.23***	(1.19; 1.27)
Outer regional	0.85***	(0.82; 0.88)	0.89***	(0.87; 0.92)	1.59***	(1.53; 1.65)
Remote	0.87***	(0.80; 0.94)	0.83***	(0.78; 0.88)	1.74***	(1.60; 1.88)
Very remote	0.92**	(0.88; 0.97)	0.83***	(0.79; 0.86)	2.33***	(2.20; 2.46)
Unknown	1.03	(0.80; 1.34)	0.77*	(0.62; 0.95)	0.95	(0.68; 1.33)
**Number of comorbidities**						
0	1	(1; 1)	1	(1; 1)	1	(1; 1)
1-2	1.81***	(1.72; 1.91)	3.54***	(3.39; 3.70)	1.18***	(1.10; 1.26)
3-5	2.77***	(2.63; 2.92)	5.96***	(5.71; 6.23)	2.01***	(1.89; 2.15)
6+	4.03***	(3.82; 4.24)	8.29***	(7.94; 8.66)	4.55***	(4.26; 4.86)
**Basis of diagnosis**						
Non-pathological	1	(1; 1)	1	(1; 1)	1	(1; 1)
Pathological	1.29***	(1.22; 1.35)	1.84***	(1.77; 1.92)	0.89***	(0.84; 0.94)
**Hospital catchment region**						
Other	1	(1; 1)	1	(1; 1)	1	(1; 1)
Bentley and Armadale	1.03*	(1.00; 1.06)	1.00	(0.98; 1.02)	0.97	(0.94; 1.00)

* p <0.05, ** p <0.01, *** p <0.001

CT: Computed tomography, ED: emergency department, RR: incidence rate ratio· ALH: All haematopoietic cancer.

RRs were also adjusted for interactions between time in months before and after cancer diagnosis and cancer groups; time in years of cancer diagnosis cohort and cancer groups; and Indigenous status. Please note Indigenous status cannot be reported as per restrictions imposed by the human research ethics approval and can only be used for model adjustment purposes. Month post diagnosis = 1 includes the day diagnosed

For peer review only

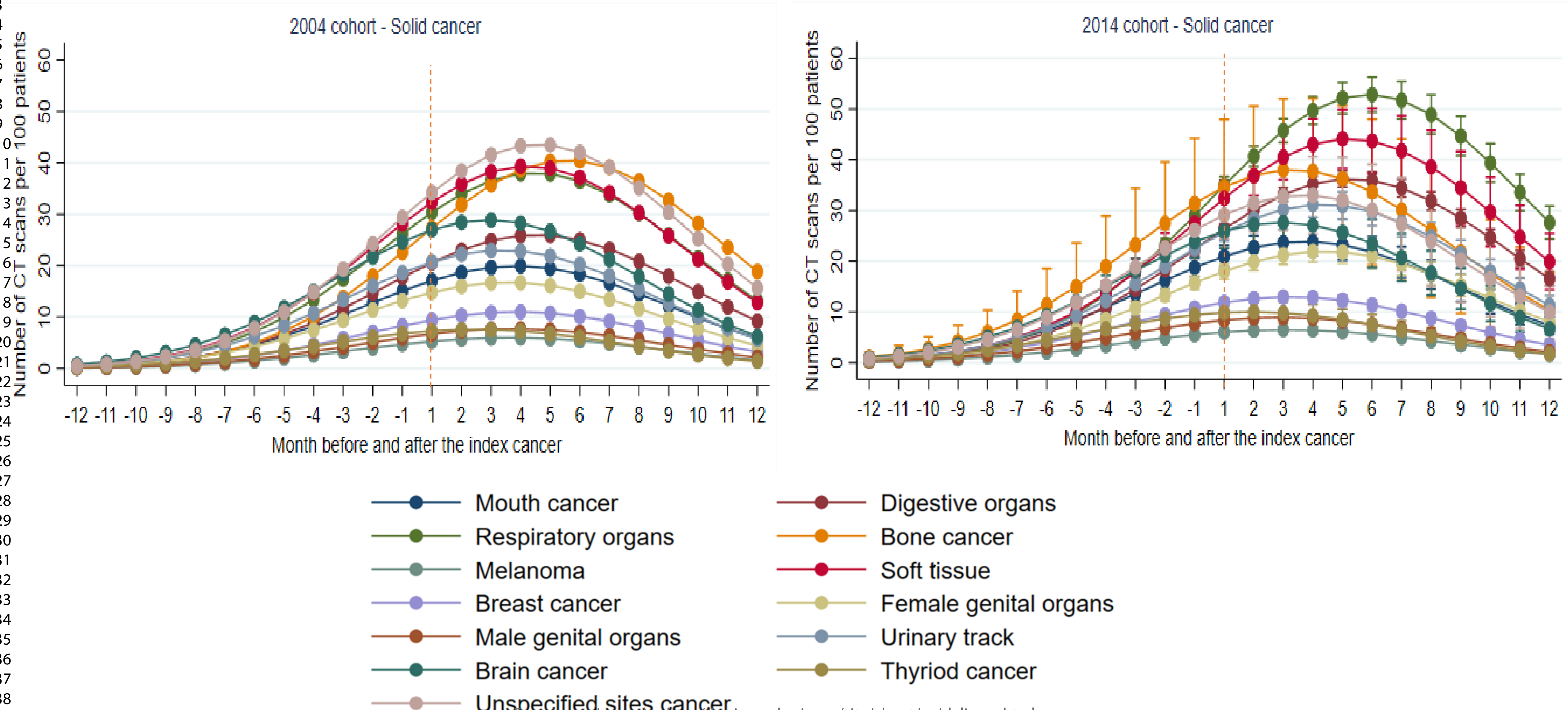

For peer review only - http://bmjopen.bmj.com/site/about/guidelines.xhtml

Appendix B1A. Trend in CT use around the cancer diagnosis window for solid cancer (12 months pre and post the index diagnosis)

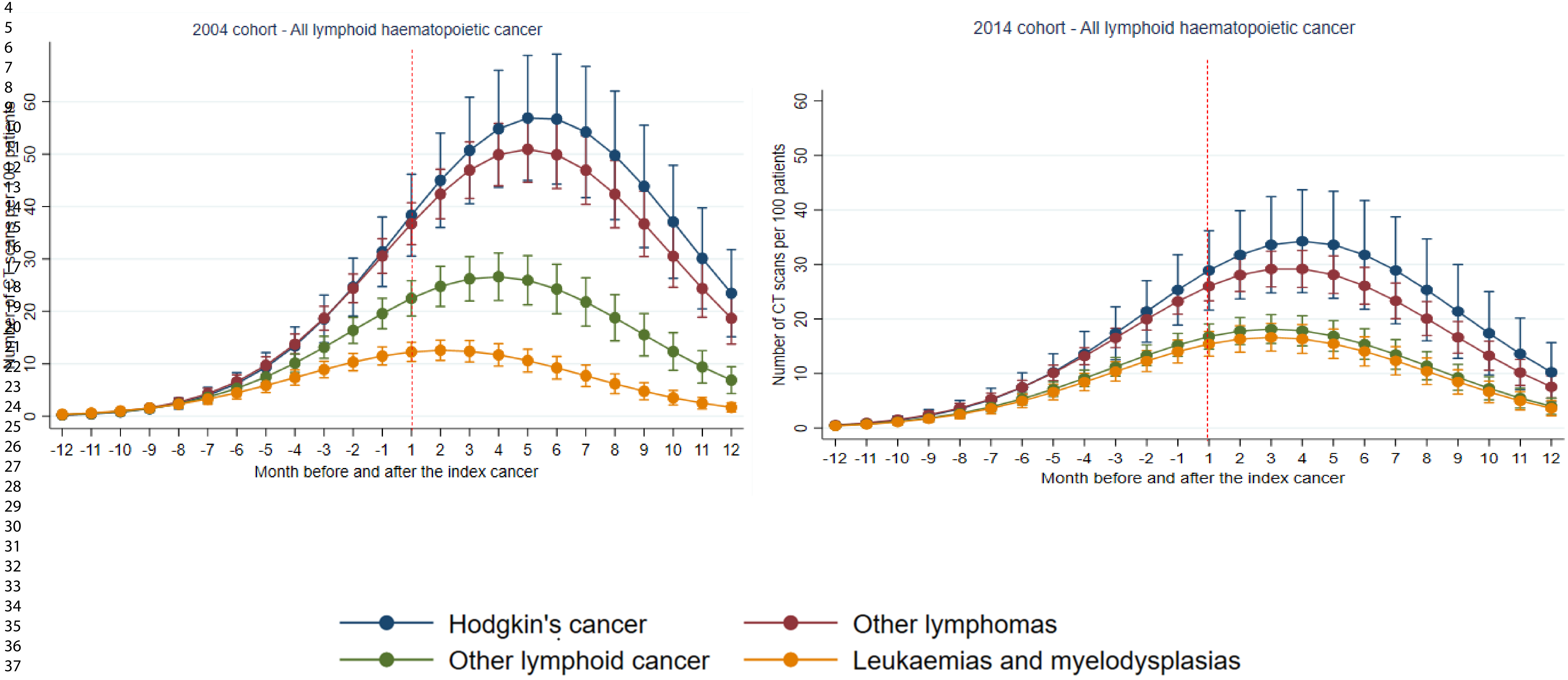

Appendix B1B. Trend in CT use around the cancer diagnosis window for ALH cancer (12 months pre and post the index diagnosis)

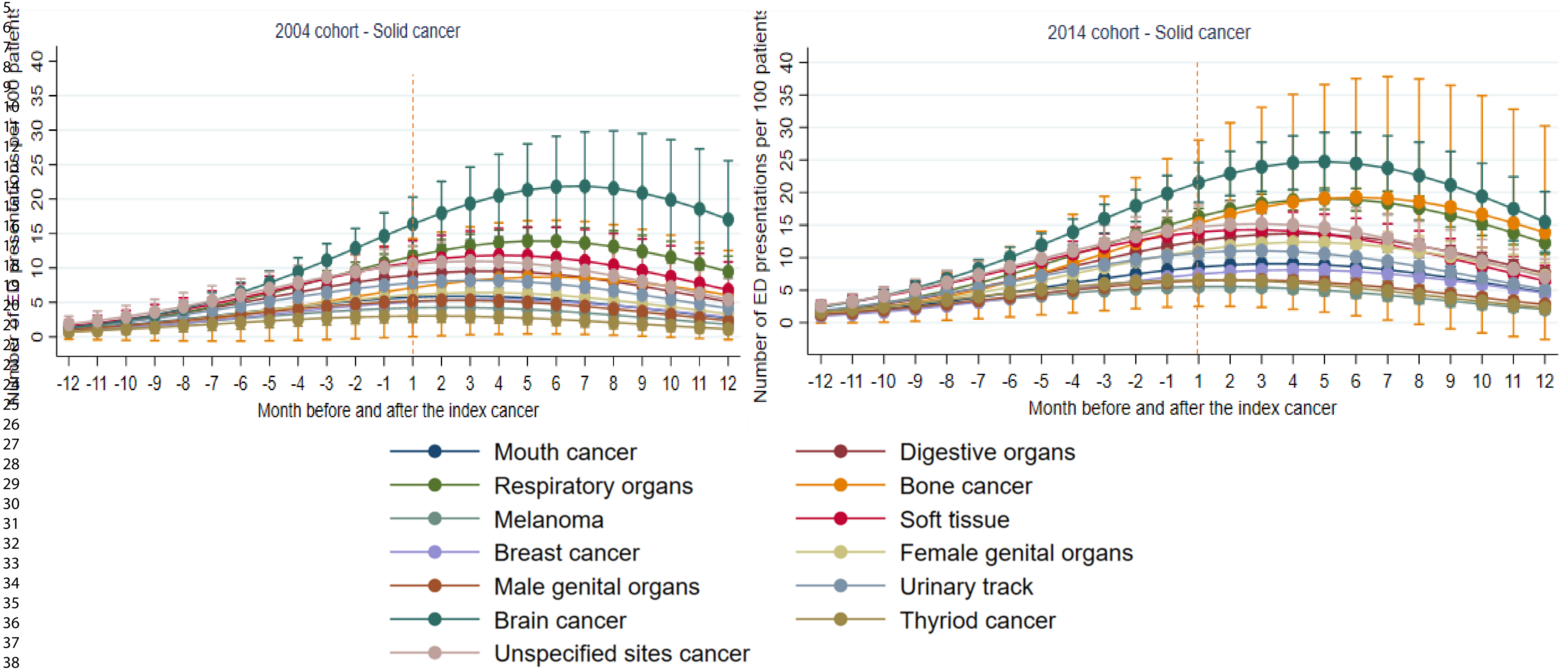

Appendix B2A. Trend in ED presentations around the cancer diagnosis window for solid cancer (12 months pre and post the index diagnosis)

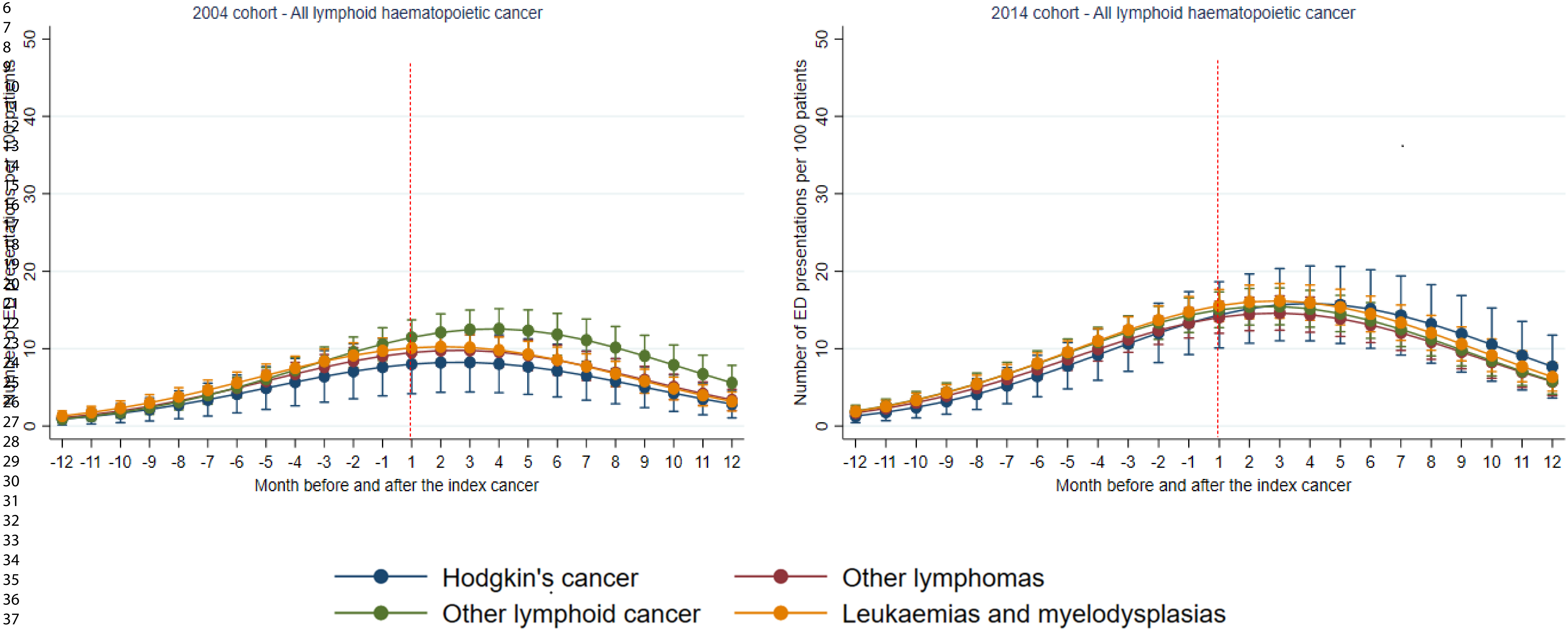

For peer review only - http://bmjopen.bmj.com/site/about/guidelines.xhtml

Appendix B2B. Trend in ED presentations around the cancer diagnosis window for ALH cancer (12 months pre and post the index diagnosis)

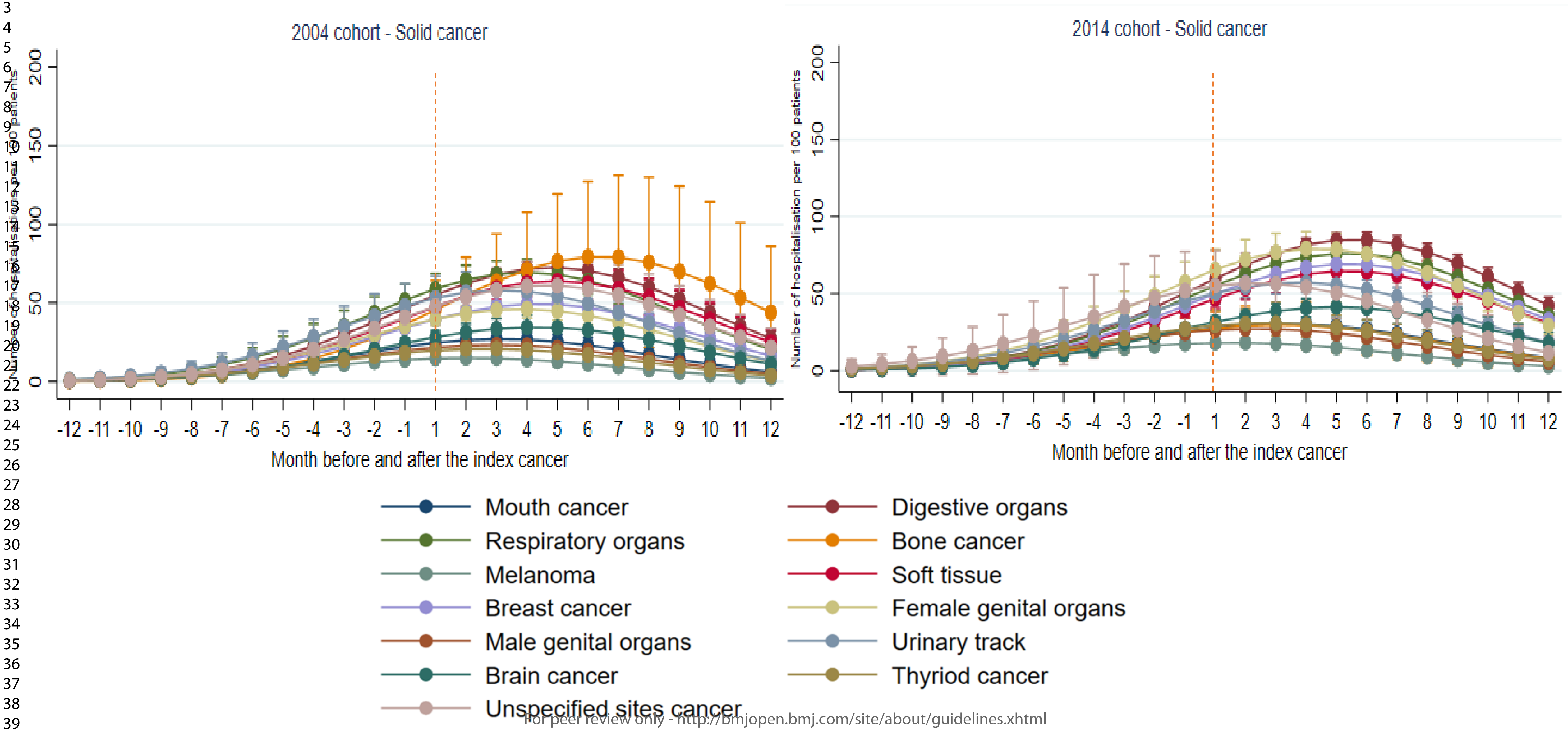

For peer review only - http://bmjopen.bmj.com/site/about/guidelines.xhtml

Appendix B3A. Trend in hospitalisation around the cancer diagnosis window for solid cancer (12 months pre and post the index diagnosis)

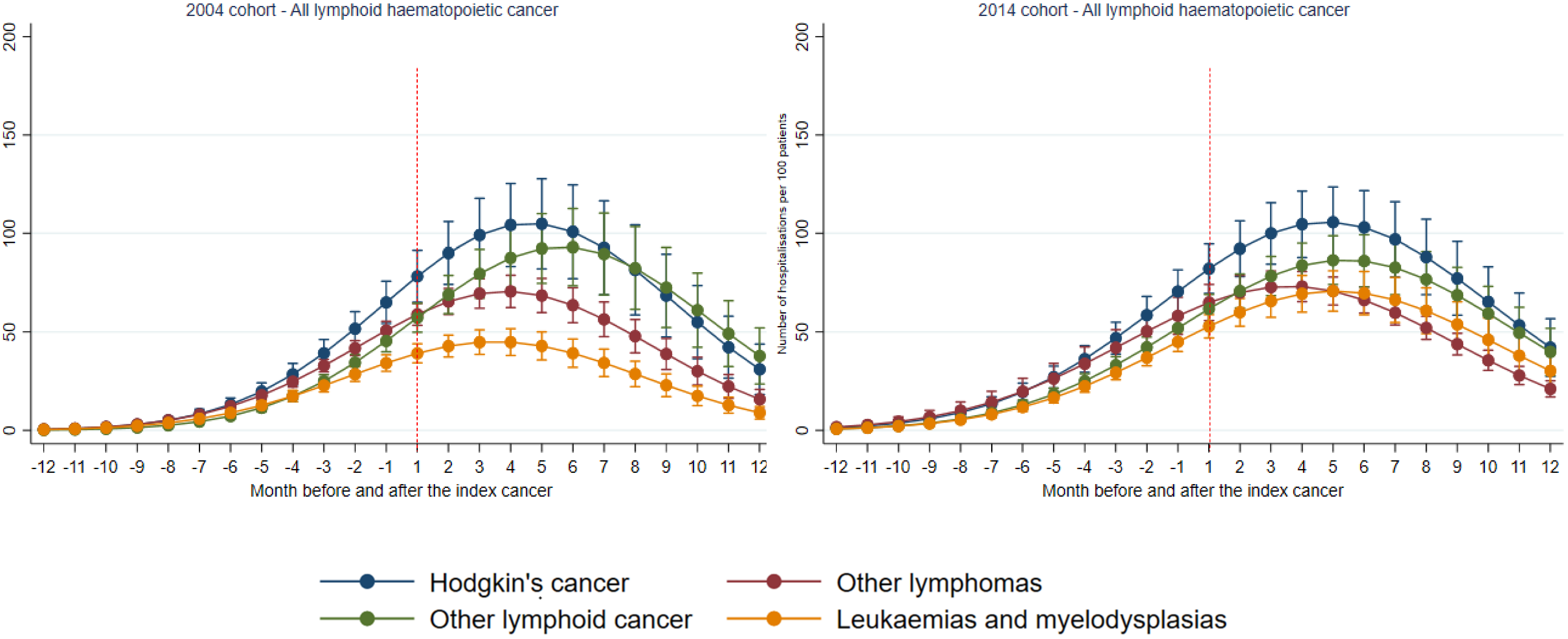

For peer review only - http://bmjopen.bmj.com/site/about/guidelines.xhtml

Appendix B3B. Trend in hospitalisation around the cancer diagnosis window for ALH cancer (12 months pre and post the index diagnosis)

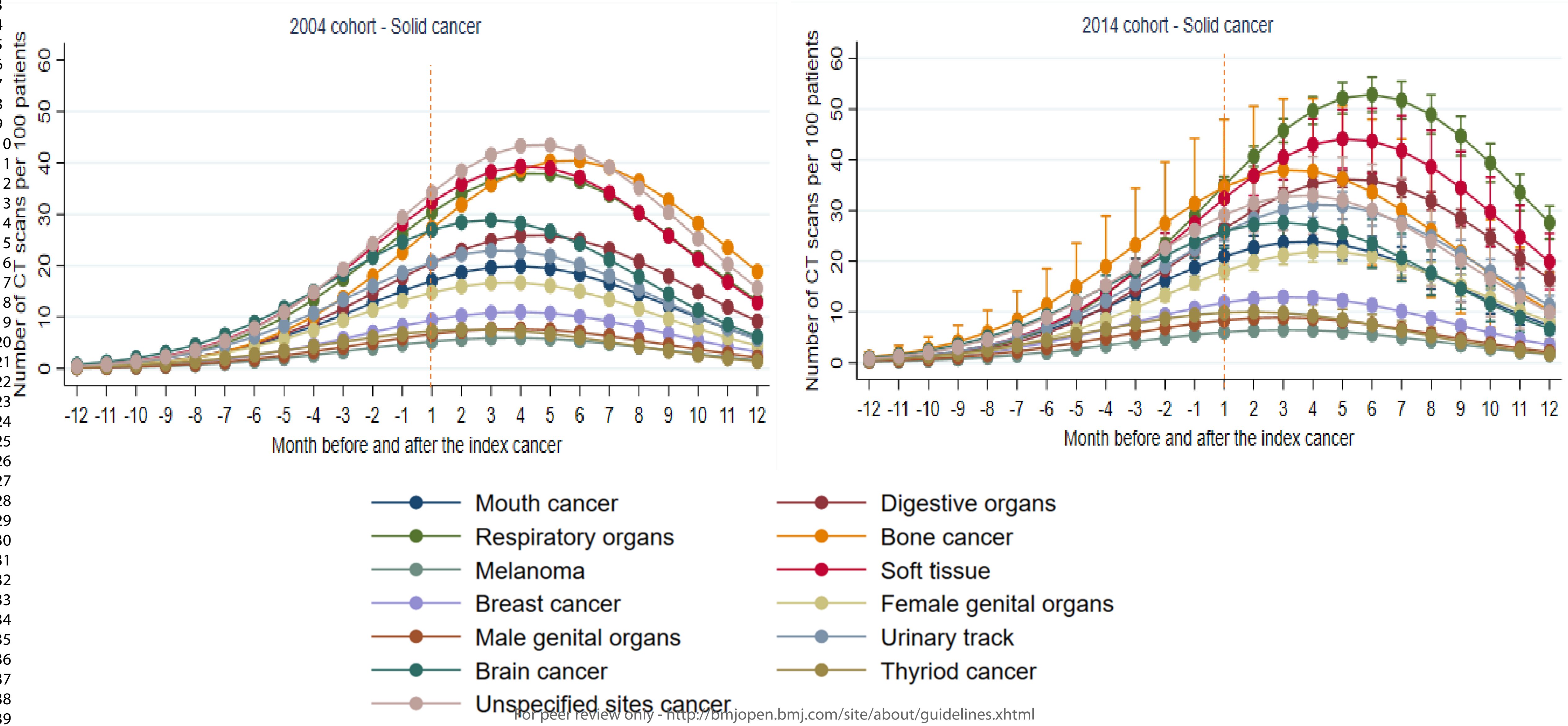

For peer review only - http://bmjopen.bmj.com/site/about/guidelines.xhtml

Appendix D1A. Trend in CT use around the cancer diagnosis window for solid cancer (12 months pre and post the index diagnosis)

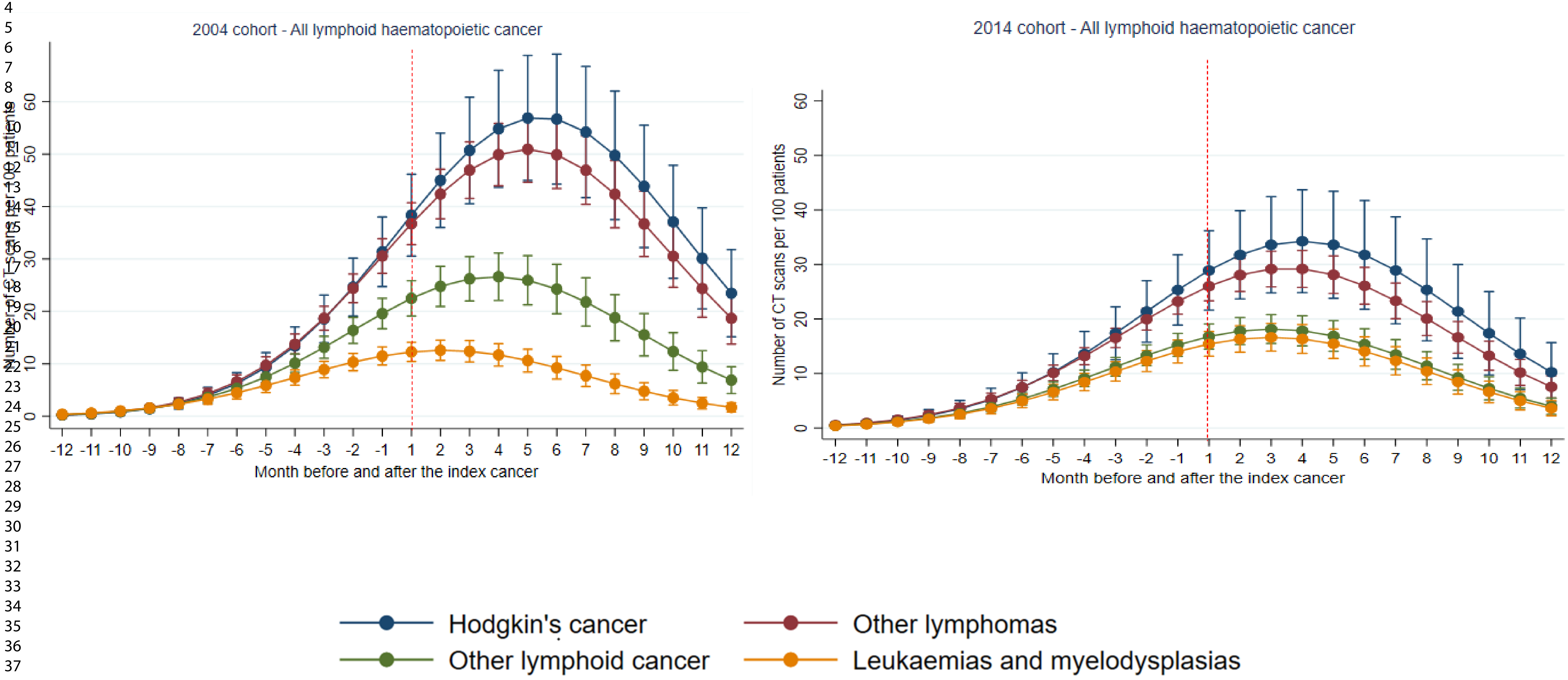

Appendix D1B. Trend in CT use around the cancer diagnosis window for ALH cancer (12 months pre and post the index diagnosis)

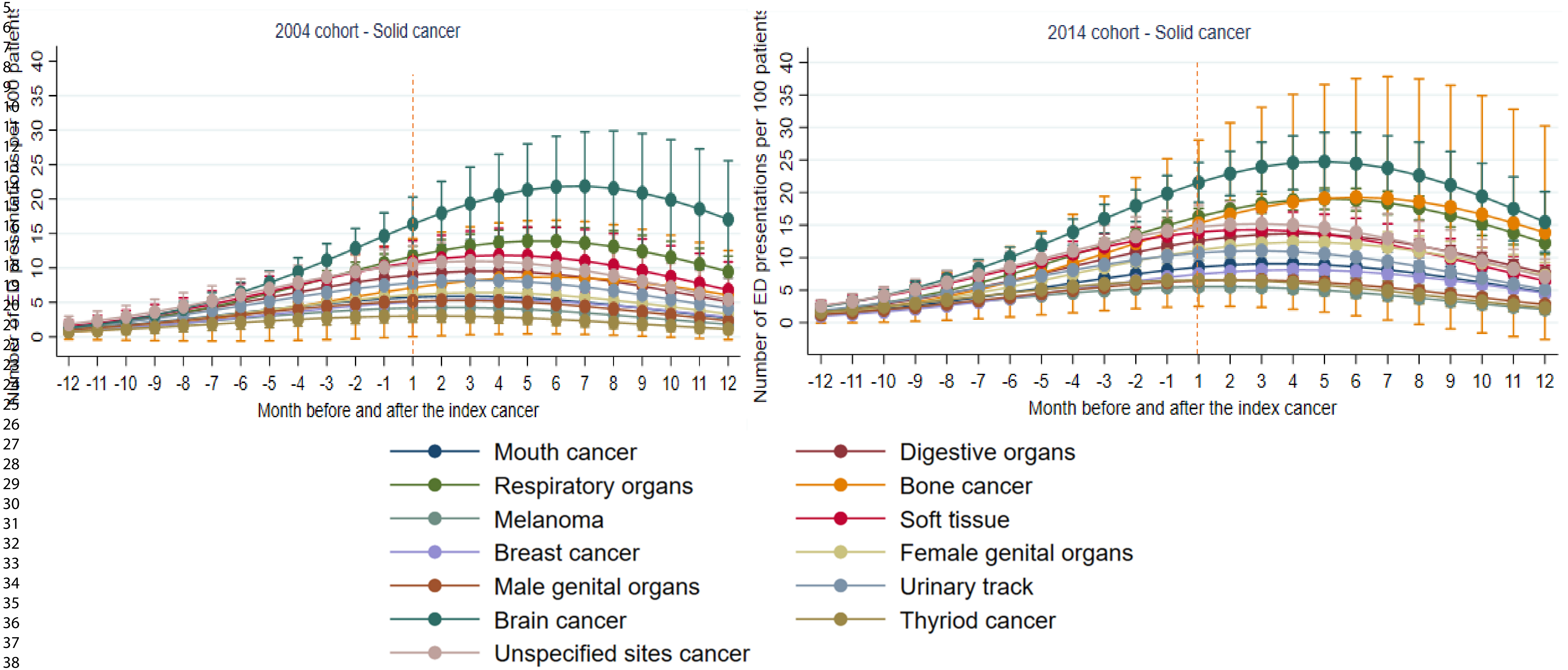

For peer review only - http://bmjopen.bmj.com/site/about/guidelines.xhtml

Appendix D2A. Trend in ED presentations around the cancer diagnosis window for solid cancer (12 months pre and post the index diagnosis)

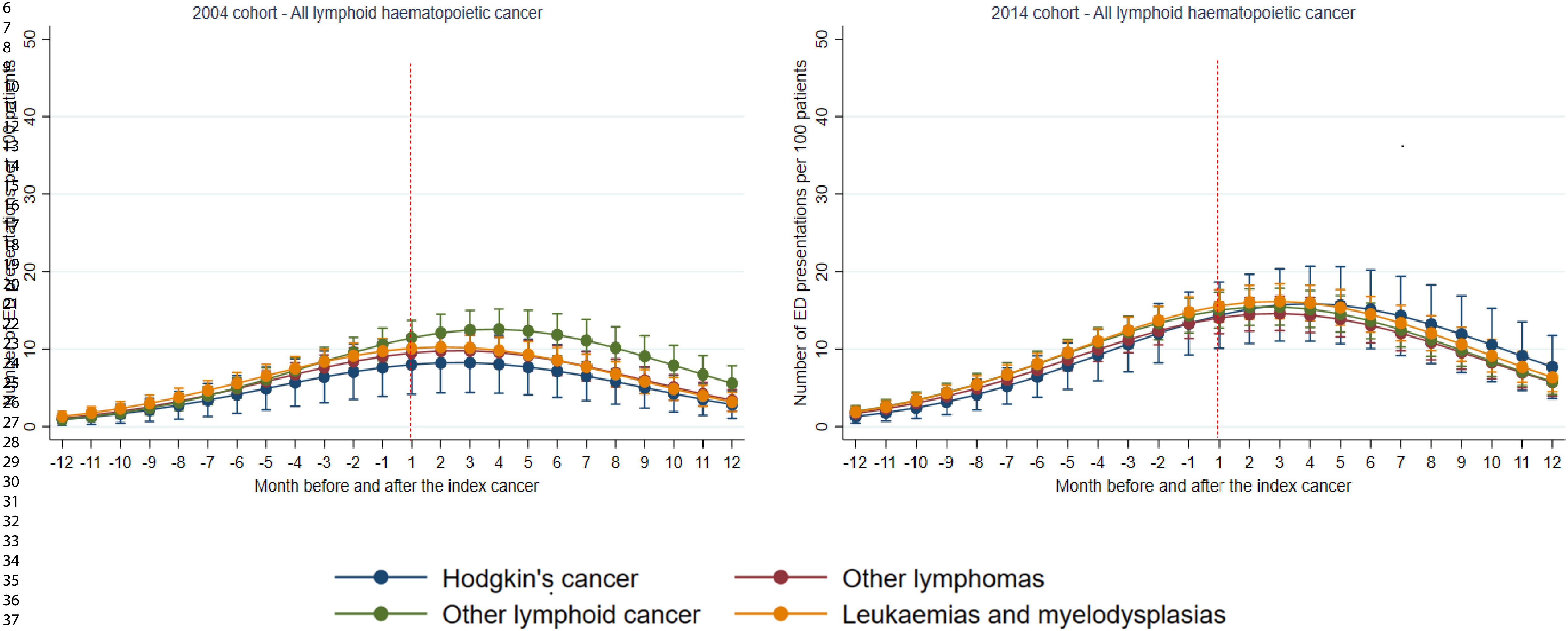

For peer review only - http://bmjopen.bmj.com/site/about/guidelines.xhtml

Appendix D2B. Trend in ED presentations around the cancer diagnosis window for ALH cancer (12 months pre and post the index diagnosis)

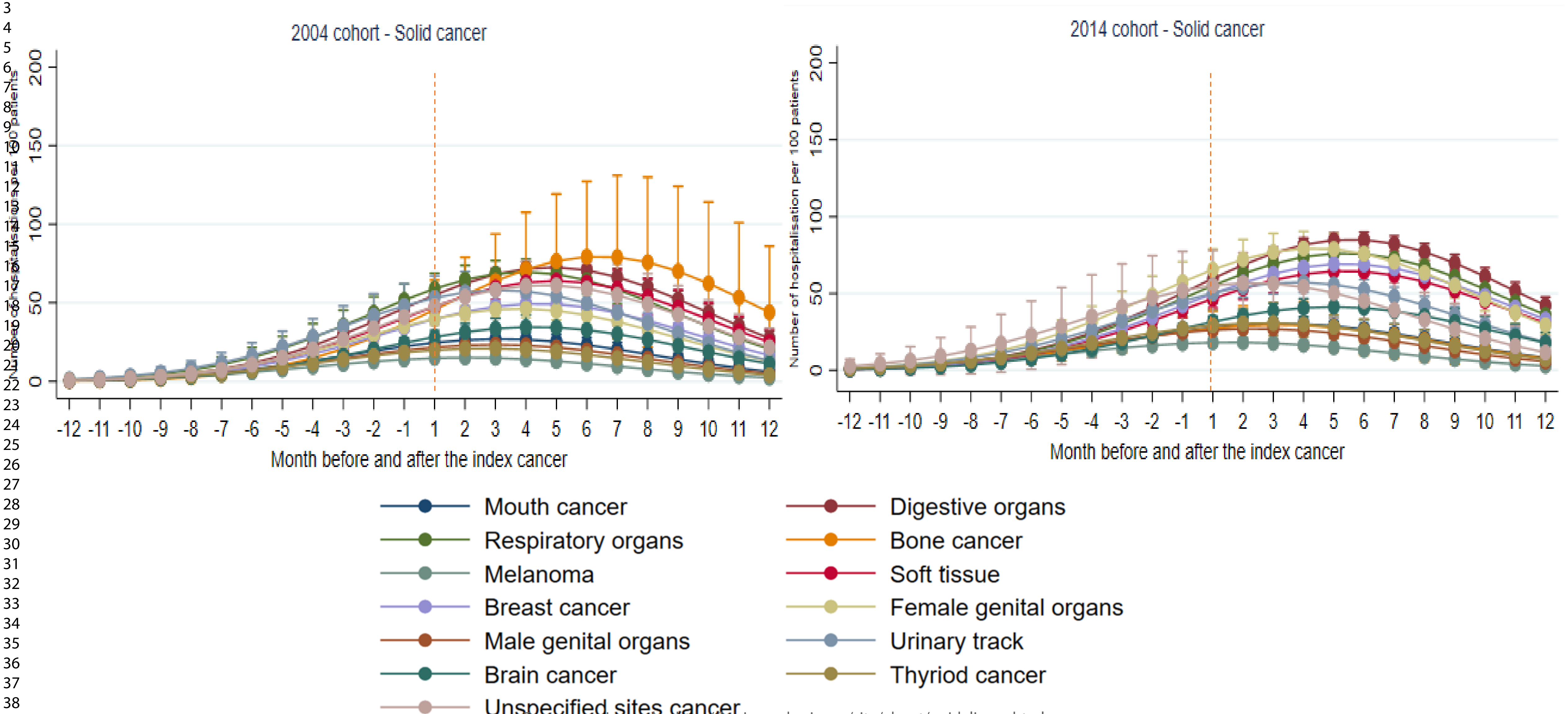

For peer review only - http://bmjopen.bmj.com/site/about/guidelines.xhtml

Appendix D3A. Trend in hospitalisation around the cancer diagnosis window for solid cancer (12 months pre and post the index diagnosis)

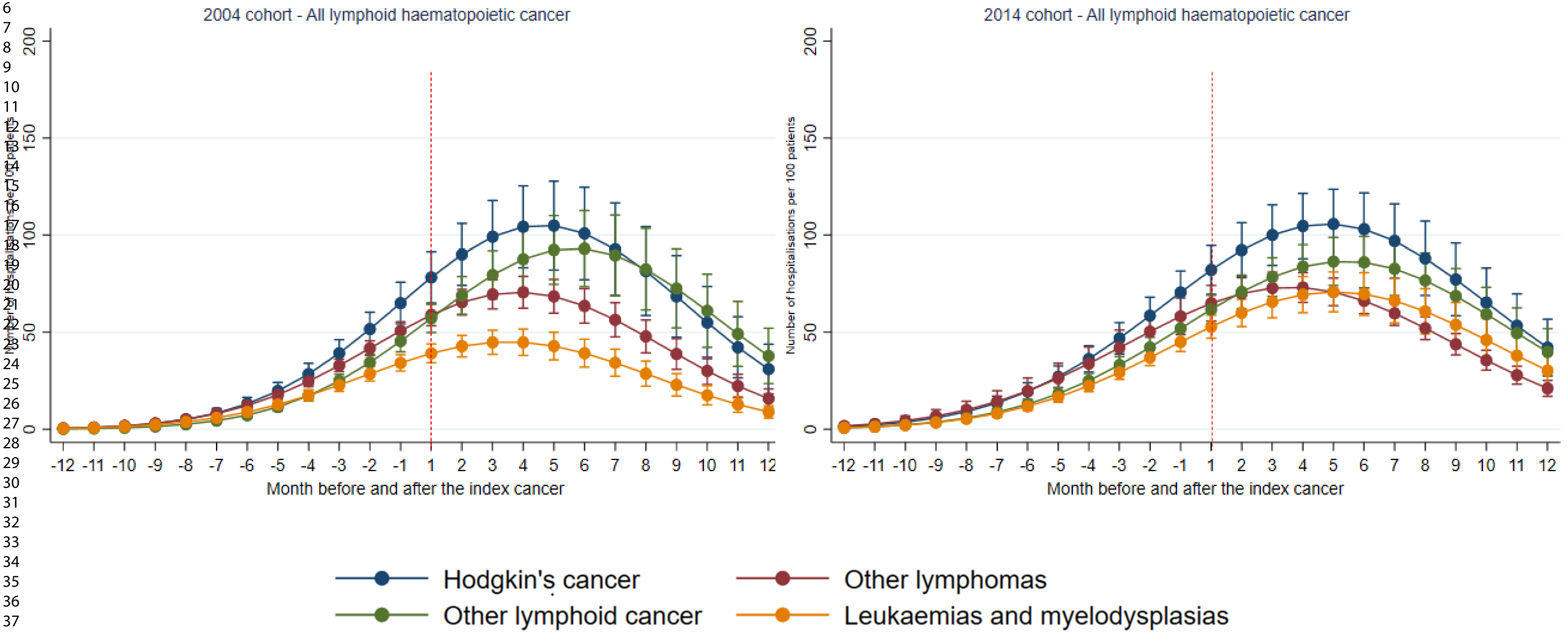

For peer review only - http://bmjopen.bmj.com/site/about/guidelines.xhtml

Appendix D3B. Trend in hospitalisation around the cancer diagnosis window for ALH cancer (12 months pre and post the index diagnosis)

The RECORD statement – checklist of items, extended from the STROBE statement, that should be reported in observational studies using routinely collected health data.

	Item No.	STROBE items	Location in manuscript where items are reported	RECORD items	Location in manuscript where items are reported
**Title and abstract**					
	1	(a) Indicate the study's design with a commonly used term in the title or the abstract (b) Provide in the abstract an informative and balanced summary of what was done and what was found		RECORD 1.1: The type of data used should be specified in the title or abstract. When possible, the name of the databases used should be included.<br><br>RECORD 1.2: If applicable, the geographic region and timeframe within which the study took place should be reported in the title or abstract.<br><br>RECORD 1.3: If linkage between databases was conducted for the study, this should be clearly stated in the title or abstract.	Title, abstract (pp. 1, 3)
**Introduction**					
Background rationale	2	Explain the scientific background and rationale for the investigation being reported			Throughout background (pp. 5-6)
Objectives	3	State specific objectives, including any prespecified hypotheses			Background, the sentence starting "In the present study, we examined …" (p.5-6)
**Methods**					
Study Design	4	Present key elements of study design early in the paper			Methods, the paragraph starting with "This population-based

For peer review only

					retrospective cohort study …" (p. 6)
Setting	5	Describe the setting, locations, and relevant dates, including periods of recruitment, exposure, follow-up, and data collection			Methods section and subheading "Data source" (p. 6)
Participants	6	*(a) Cohort study* - Give the eligibility criteria, and the sources and methods of selection of participants. Describe methods of follow-up<br>*Case-control study* - Give the eligibility criteria, and the sources and methods of case ascertainment and control selection. Give the rationale for the choice of cases and controls<br>*Cross-sectional study* - Give the eligibility criteria, and the sources and methods of selection of participants<br><br>*(b) Cohort study* - For matched studies, give matching criteria and number of exposed and unexposed<br>*Case-control study* - For matched studies, give matching criteria and the number of controls per case		RECORD 6.1: The methods of study population selection (such as codes or algorithms used to identify subjects) should be listed in detail. If this is not possible, an explanation should be provided.<br><br>RECORD 6.2: Any validation studies of the codes or algorithms used to select the population should be referenced. If validation was conducted for this study and not published elsewhere, detailed methods and results should be provided.<br><br>RECORD 6.3: If the study involved linkage of databases, consider use of a flow diagram or other graphical display to demonstrate the data linkage process, including the number of individuals with linked data at each stage.	Methods subheading "Study population" (p.7)<br><br>No validation study to report<br><br>Described in the "Method" section. (pp.6-7)
Variables	7	Clearly define all outcomes, exposures, predictors, potential confounders, and effect modifiers. Give diagnostic criteria, if applicable.		RECORD 7.1: A complete list of codes and algorithms used to classify exposures, outcomes, confounders, and effect modifiers should be provided. If these cannot be reported, an explanation should be provided.	Methods subheadings "Use of CT", "Hospitalisations and ED Presentations"

For peer review only - http://bmjopen.bmj.com/site/about/guidelines.xhtml

					and "Covariates" (p. 8).
Data sources/ measurement	8	For each variable of interest, give sources of data and details of methods of assessment (measurement). Describe comparability of assessment methods if there is more than one group			Methods subheadings "Outcome measures", and "Covariates" (p. 8).
Bias	9	Describe any efforts to address potential sources of bias			Methods subheadings "Covariates" and "Analysis" (pp. 8-9)
Study size	10	Explain how the study size was arrived at			Study population (page 7)
Quantitative variables	11	Explain how quantitative variables were handled in the analyses. If applicable, describe which groupings were chosen, and why			Methods subheading "Outcome measures", "Covariates" (p. 8) and Table 1.
Statistical methods	12	(a) Describe all statistical methods, including those used to control for confounding<br>(b) Describe any methods used to examine subgroups and interactions<br>(c) Explain how missing data were addressed<br>(d) *Cohort study* - If applicable, explain how loss to follow-up was addressed<br>*Case-control study* - If applicable, explain how matching of cases and controls was addressed			Methods subheading "Analysis" (p.9)

For peer review only

		Cross-sectional study - If applicable, describe analytical methods taking account of sampling strategy<br>(e) Describe any sensitivity analyses			
Data access and cleaning methods		..		RECORD 12.1: Authors should describe the extent to which the investigators had access to the database population used to create the study population.<br><br>RECORD 12.2: Authors should provide information on the data cleaning methods used in the study.	
Linkage		..		RECORD 12.3: State whether the study included person-level, institutional-level, or other data linkage across two or more databases. The methods of linkage and methods of linkage quality evaluation should be provided.	Linkage described in "Methods" section (p.6)
**Results**					
Participants	13	(a) Report the numbers of individuals at each stage of the study (*e.g.*, numbers potentially eligible, examined for eligibility, confirmed eligible, included in the study, completing follow-up, and analysed)<br>(b) Give reasons for non-participation at each stage.<br>(c) Consider use of a flow diagram		RECORD 13.1: Describe in detail the selection of the persons included in the study (*i.e.,* study population selection) including filtering based on data quality, data availability and linkage. The selection of included persons can be described in the text and/or by means of the study flow diagram.	Results (p.10)
Descriptive data	14	(a) Give characteristics of study participants (*e.g.*, demographic, clinical, social) and information			Participants described in results section

For peer review only - http://bmjopen.bmj.com/site/about/guidelines.xhtml

		on exposures and potential confounders<br>(b) Indicate the number of participants with missing data for each variable of interest<br>(c) *Cohort study* - summarise follow-up time (*e.g.*, average and total amount)			(p.10) and Table 1.
Outcome data	15	*Cohort study* - Report numbers of outcome events or summary measures over time<br>*Case-control study* - Report numbers in each exposure category, or summary measures of exposure<br>*Cross-sectional study* - Report numbers of outcome events or summary measures			Tables 1.
Main results	16	(a) Give unadjusted estimates and, if applicable, confounder-adjusted estimates and their precision (e.g., 95% confidence interval). Make clear which confounders were adjusted for and why they were included<br>(b) Report category boundaries when continuous variables were categorized<br>(c) If relevant, consider translating estimates of relative risk into absolute risk for a meaningful time period			Figures 1-2, Table 2 and Appendices A-C.
Other analyses	17	Report other analyses done—e.g., analyses of subgroups and interactions, and sensitivity analyses			Appendix D
**Discussion**					

For peer review only

Key results	18	Summarise key results with reference to study objectives			Throughout discussion and the main conclusions are in section "Conclusion" (pp.21)
Limitations	19	Discuss limitations of the study, taking into account sources of potential bias or imprecision. Discuss both direction and magnitude of any potential bias		RECORD 19.1: Discuss the implications of using data that were not created or collected to answer the specific research question(s). Include discussion of misclassification bias, unmeasured confounding, missing data, and changing eligibility over time, as they pertain to the study being reported.	Discussed in a sub-section "Strengths and limitations" (p.20)
Interpretation	20	Give a cautious overall interpretation of results considering objectives, limitations, multiplicity of analyses, results from similar studies, and other relevant evidence			Throughout discussion
Generalisability	21	Discuss the generalisability (external validity) of the study results		all, or who presented to any public hospital emergency department (ED), or who had a CT scan undertaken in WA by a public or private provider between 1 January 2003 and 31 December 2015	In Method, we state that contains Western Australian data for instance "…Western Australians aged 18+ who had a separation (discharge) from any WA hospital for any non-pregnancy-related condition …" (p. 6), and throughout

For peer review only

					Discussion (p. 17) with more specific statements in limitation sub-section (pp. 20).
**Other Information**					
Funding	22	Give the source of funding and the role of the funders for the present study and, if applicable, for the original study on which the present article is based			Funding heading (p.22)
Accessibility of protocol, raw data, and programming code		..		RECORD 22.1: Authors should provide information on how to access any supplemental information such as the study protocol, raw data, or programming code.	The data that support the findings of this study are available from the relevant data custodians of the study datasets. Restrictions by the data custodians mean that the data are not publicly available or able to be provided by the authors. Researchers wishing to access the datasets used in this study should refer to the Western Australian Data Linkage Unit and the Australian Institute of Health

For peer review only

					and Welfare. (p. 22).

*Reference: Benchimol EI, Smeeth L, Guttmann A, Harron K, Moher D, Petersen I, Sørensen HT, von Elm E, Langan SM, the RECORD Working Committee. The REporting of studies Conducted using Observational Routinely-collected health Data (RECORD) Statement. *PLoS Medicine* 2015; in press.

*Checklist is protected under Creative Commons Attribution (CC BY) license.

For peer review only
